# The mechanism of action of a novel neuroprotective low molecular weight dextran sulphate: New platform therapy for neurodegenerative diseases like Amyotrophic Lateral Sclerosis

**DOI:** 10.3389/fphar.2022.983853

**Published:** 2022-08-30

**Authors:** Ann Logan, Antonio Belli, Valentina Di Pietro, Barbara Tavazzi, Giacomo Lazzarino, Renata Mangione, Giuseppe Lazzarino, Inés Morano, Omar Qureshi, Lars Bruce, Nicholas M. Barnes, Zsuzsanna Nagy

**Affiliations:** ^1^ Department of Biomedical Sciences, University of Warwick, Coventry, United Kingdom; ^2^ Axolotl Consulting Ltd., Droitwich, United Kingdom; ^3^ College of Medical and Dental Sciences, University of Birmingham, Birmingham, United Kingdom; ^4^ UniCamillus-Saint Camillus International University of Health and Medical Sciences, Rome, Italy; ^5^ Department of Basic Biotechnological Sciences, Intensive and Perioperative Clinics, Catholic University of Rome, Rome, Italy; ^6^ Department of Biomedical and Biotechnological Sciences, Division of Medical Biochemistry, University of Catania, Catania, Italy; ^7^ Celentyx Ltd., Birmingham, United Kingdom; ^8^ Tkomed AB, Viken, Sweden

**Keywords:** amyotrophic lateral sclerosis, low molecular weight-dextran sulphate, heparin-binding growth factors, glutamate, metabolism, inflammation, neurodegeneration, traumatic brain injury

## Abstract

**Background:** Acute and chronic neurodegenerative diseases represent an immense socioeconomic burden that drives the need for new disease modifying drugs. Common pathogenic mechanisms in these diseases are evident, suggesting that a platform neuroprotective therapy may offer effective treatments. Here we present evidence for the mode of pharmacological action of a novel neuroprotective low molecular weight dextran sulphate drug called ILB^®^. The working hypothesis was that ILB^®^ acts via the activation of heparin-binding growth factors (HBGF).

**Methods:** Pre-clinical and clinical (healthy people and patients with ALS) *in vitro* and *in vivo* studies evaluated the mode of action of ILB^®^. *In vitro* binding studies, functional assays and gene expression analyses were followed by the assessment of the drug effects in an animal model of severe traumatic brain injury (sTBI) using gene expression studies followed by functional analysis. Clinical data, to assess the hypothesized mode of action, are also presented from early phase clinical trials.

**Results:** ILB^®^ lengthened APTT time, acted as a competitive inhibitor for HGF-Glypican-3 binding, effected pulse release of heparin-binding growth factors (HBGF) into the circulation and modulated growth factor signaling pathways. Gene expression analysis demonstrated substantial similarities in the functional dysregulation induced by sTBI and various human neurodegenerative conditions and supported a cascading effect of ILB^®^ on growth factor activation, followed by gene expression changes with profound beneficial effect on molecular and cellular functions affected by these diseases. The transcriptional signature of ILB^®^ relevant to cell survival, inflammation, glutamate signaling, metabolism and synaptogenesis, are consistent with the activation of neuroprotective growth factors as was the ability of ILB^®^ to elevate circulating levels of HGF in animal models and humans.

**Conclusion:** ILB^®^ releases, redistributes and modulates the bioactivity of HBGF that target disease compromised nervous tissues to initiate a cascade of transcriptional, metabolic and immunological effects that control glutamate toxicity, normalize tissue bioenergetics, and resolve inflammation to improve tissue function. This unique mechanism of action mobilizes and modulates naturally occurring tissue repair mechanisms to restore cellular homeostasis and function. The identified pharmacological impact of ILB^®^ supports the potential to treat various acute and chronic neurodegenerative disease, including sTBI and ALS.

## Introduction

Neurodegeneration is responsible for many acute and chronic progressive diseases that affect the nervous system. The permanent loss of function experienced by affected individuals results from a combination of neuronal and glial damage. The burden of these neurological diseases is increasing inexorably, in part due to ageing populations. In 2017 in the WHO European region alone the total number of disability adjusted life years attributable to neurological disorders was more than 41 million with nearly 2 million deaths (Deuschl et al., 2020). While the last 4 decades have brought enormous progress in understanding risk factors, mechanisms of disease progression, genetic influences and creating preclinical disease models, the pathogenesis of these diseases is still far from elucidated. The numerous clinical trial failures of the past 2 decades in neurodegenerative diseases, particularly those involving patients with Alzheimer’s disease (AD) and amyotrophic lateral sclerosis (ALS), are partly due to the chosen therapeutic targets (often end-stage pathologies or single etiological factors thought to contribute to the disease), to the use of inadequate preclinical models (transgenic animals) or to deficient clinical trial design (relatively late-stage disease, heterogenous patient populations without adequate stratification) (Imbimbo and Watling, 2021; Sever et al., 2022). The upshot of these failures is a growing understanding of the need to identify different targets and therapeutic strategies and to improve clinical trial design.

We have reported recently the successful outcome of an open phase II clinical trial in ALS using a novel low molecular weight dextran sulphate (ILB^®^) that displays potential to treat a range of neurodegenerative diseases in addition to ALS (Logan et al., 2022). The ILB^®^ induced APTT changes and HGF release in this study, lead us to hypothesize that ILB^®^ interacts with heparin-binding proteins and growth factors initiating a downstream cascade that may be responsible for its neuroprotective properties. In this paper we report a detailed evaluation of the mode of action of this drug.

We present preclinical and clinical studies demonstrating that this novel low molecular weight dextran sulphate (LMW-DS), called ILB^®^, interacts with heparin-binding growth factors (HBGF) and acts to mobilize and modulate the activity of endogenous neurotrophic and myotrophic growth factors responsible for normal cell function, survival and repair. Furthermore, we validate a rodent model of neurodegeneration to establish its relevance to chronic human neurodegenerative diseases. Using this model we demonstrate the ability of ILB^®^ to restore to homeostasis the dysregulated neurotransmitter, metabolic and immune processes directly relevant to the pathogenesis of neurodegenerative diseases, including ALS.

## Materials and methods

### Description of the medicinal product ILB^®^

**FIGURE 1:**
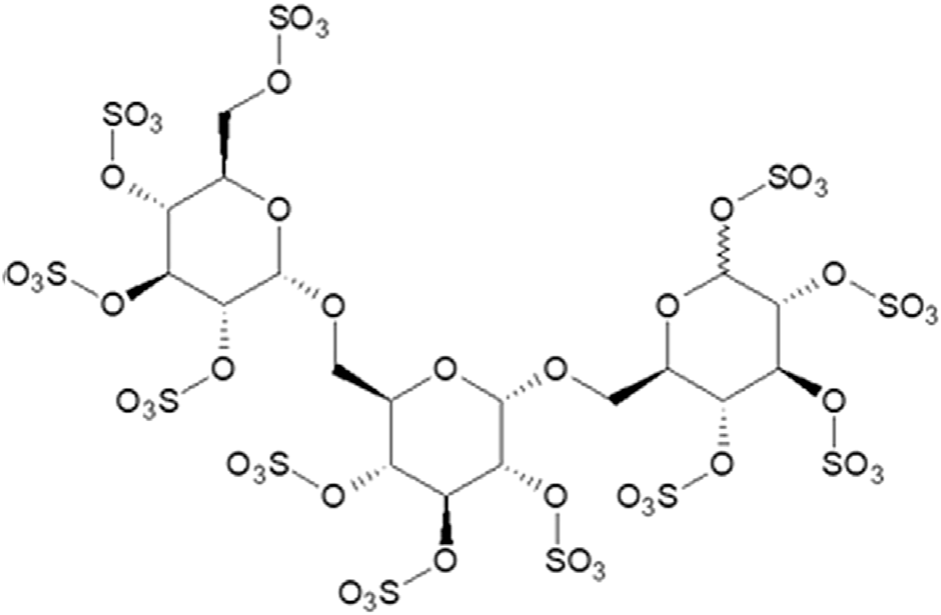
ILB^®^ structure.

ILB^®^ is the sodium salt of LMW-DS containing 16–19% sulphur with an average Mn of 5 kDa and contains molecules spanning approximately 2–8 kDa (average Mn for the ILB^®^ batch used in all preclinical and clinical studies described: Mn = 2241Da) (Figure 1). It is supplied as the sodium salt which is a white to off-white powder freely soluble in water and salt solutions (100 mg/ml). The powder shows excellent long-term stability at room temperature when stored in air-tight containers. The stability of the powder form of ILB^®^ is optimal at pH 5.5–8 at room temperature when stored in air-tight containers. The drug product is prepared in two strengths for pre-clinical and clinical studies, 20 mg/ml and 100 mg/ml, dissolved in 0.9% NaCl in water for injection. The solution is stored at 4°C.

### *Ex vivo* competitive ELISA assay: ILB^®^ effect on HGF and Glypican-3 binding

We designed an ELISA to study the ability of ILB^®^ to inhibit the interaction between HGF and Glypican-3 (a cell surface heparan sulphate proteoglycan) (Gao et al., 2015). The bound Glypican-3 (biotinylated) in the system is measured. The amount of Glypican signal lost (relative to the expected binding) is a measure of the ability of ILB^®^ to mobilize HGF from its binding to Glypican-3.

Briefly, the ELISA plate was coated with HGF (0.2 μg/ml, 100 µl/well, R&D Systems, Abingdon, United Kingdom) overnight at 4°C. Glypican-3 (R&D Systems) binding was quantified after 30 min over a range of concentrations in the absence (vehicle) or presence of a range of concentrations of ILB^®^. After three washes with PBS, streptavidin-HRP (according to manufacturer’s recommendations for the batch used, R&D Systems) was added to the wells for 1 h to label the bound Glypican-3. After a further 3 washes, the remaining HRP activity was quantified using an HRP substrate (DY999, R&D Systems) at 450 and 540 nm, with Glypican-3 levels determined by reference to a standard calibration curve. All conditions were assayed in triplicate.

### *Ex vivo* ATPP and anti-FXII assay

To demonstrate the potential effect of ILB^®^ on blood coagulation, ATPP and anti-FXII assays were performed using different ILB^®^ concentrations added to human healthy serum. The ATPP and antiFXII assays were carried out by the Medicinsk Service, Klinisk kemioch Farmakologi (Malmo, Sweden) clinical laboratory using clinical diagnostic protocols. All assays were carried out in duplicate.

### Evaluation of ILB^®^ effect on growth factor signalling *in vitro*

HEK-Blue TGFβ reporter cells (human embryonic kidney cells stably transfected with human TGFβRI and the TGFβ signaling proteins, Smad3 and Smad4, along with Smad3/4-binding elements (SBE)-inducible secreted embryonic alkaline phosphatase (SEAP) reporter were used for this assay with transgene expression maintained using the expression selection antibiotics, blasticidin, hygromycin B and zeocin (Invivogen, Hong Kong). Different concentrations of ILB^®^, Low Molecular Weight Heparin (third International Standard; NIBSC, Potters Bar, United Kingdom, 11/176) or vehicles 1 and 2 (saline solution and water, respectively) with recombinant human TGFβ (Invitrogen, Oxford, United Kingdom) or vehicle (0.1% PBS) were incubated at room temperature in flat 96-well plates for an hour. The HEK-Blue TGFβ reporter cells were then added (50,000 cells/well) and incubated for 24 h at 37°C following manufacturer instructions. Subsequently, cell culture supernatants were collected and incubated with QUANTI-Blue™ Solution (Invivogen) for 60 min at 37°C before the optical density was measured at 655 nm using a microplate reader (iMark; Biorad, Watford, United Kingdom).

### *Ex vivo* immunological analysis of ILB^®^ effects

Peripheral blood mononuclear cells (PBMC) were isolated from 10 healthy human donors through Ficoll-Paque PLUS (GE Healthcare, Chalfont Saint Giles, United Kingdom; 11778538) density centrifugation. Monocytes were purified by negative selection using the EasySep™ human monocyte enrichment kit (StemCell Technologies, Cambridge, United Kingdom). Monocytes were cultured in the absence (unstimulated PBS vehicle) or presence of stimulation (0.01 ng/ml LPS) in the absence (vehicle) or presence of ILB^®^ (60–600 μg/ml, concentrations spanning the ILB^®^ blood concentrations achieved in human subjects in EudraCT No 2011-004111-23 and above), low molecular weight heparin (2.0–20 μg/ml; equivalent to 0.406, 1.218 and 4.06 units/ml (1.218 units/ml being an approximate therapeutic concentration); Sigma Aldrich, Poole, United Kingdom) or dexamethasone (3.0 µM; Sigma Aldrich) for 24 h at 37°C, 5% CO_2_. Following centrifugation, cell culture supernatants were removed and stored at −20°C before analysis. IL-6 levels were quantified in the supernatant by ELISA (R&D Systems) according to the manufacturer’s instructions. The Mann Whitney *U* test was used to analyze the HEK cell growth factor signaling and the PBMC immunological data and a *p* value of <0.05 was considered as significant.

### *In vitro* transcriptional effects of ILB^®^

Human Schwann-like cells (CRL-2884) and SHSY5Y neuroblastoma cells (CRL-2266) were purchased from ATTC (Manassas, Virginia, United States). Schwann cells were grown in high-glucose DMEM supplemented with 10% of FCS and incubated at 37°C with 5% CO_2_ for 48 h before drug treatment. SHSY5Y cells were grown in DMEM:F12 medium supplemented with 10% FCS and 1% GlutaMAX™-I Supplement (all from ThermoFisher Scientific, Loughborough, United Kingdom). Cells were incubated at 37°C with 5% CO_2_ for 48 h before drug treatment. After the initial incubation time, some of the cultures were treated with ILB^®^ (0.01, 0.1, 1.0 and 10 μg/ml), while others received vehicle only. The chosen ILB^®^ concentrations encompass the ILB^®^ concentrations achieved with therapeutic doses of ILB^®^ (maximum concentration of ILB^®^ in blood after the therapeutic dose of 1 mg/kg is ∼5 μg/ml in EudraCT No 2017-005065-47). One set of samples for each cell type was collected at this point to represent the ‘Day 0’ control. Following another 24 h, the cells were collected into RNAlater^®^ Solution (Invitrogen Life Technologies, Loughborough, United Kingdom) and left at room temperature for 24 h. Cells were then moved into the refrigerator at -4°C for another 2 weeks before processing. RNA extraction and array analysis was performed by SourceBioscience (Nottingham, United Kingdom) using Agilent SurePrint Microarrays (Agilent Technologies United Kingdom, Cheadle, United Kingdom). The expression data were downloaded into separate files for each cell line. The “Background corrected” expression data from the arrays were extracted and log2 transformed. To reduce the false discovery 1rate, the signals that were below “expression level” (set at 5 for the log2 transformed expression values) were removed.

Based on the expression pattern of the Control probes on each array Median centering was carried out for all arrays before analysis. Data were grouped by cell type and each cell type analyzed in the following algorithms:

1. Comparison of day (d)0 control to d2 control samples—expression changes seen in the cells in normal cultures.
2. Comparison of d2 control to d2 treated samples—differential expression induced by the dug in the culture.

A preliminary analysis was executed to screen out genes that were not differentially expressed between any combination of the three datasets. Simple, non-stringent ANOVA (*p* < 0.05) was used to look for patterns of expression. Probes with no changes across the three datasets were eliminated. The remaining probe sets were analyzed for fold change and significance using Volcano plots. More than 10% change in the expression of a probe (FC=>1.1 or FC=<0.90) was regarded as significant in the first instance to allow the detection of expression patterns.

### Rodent model of neurodegeneration (severe traumatic brain injury)

The rat model of severe traumatic brain injury (sTBI) that induces diffuse axonal injury and progressive neurodegeneration (Foda and Marmarou, 1994) was approved by the Ethical Committee of the Catholic University of the Sacred Heart of Rome, Italy (approval 1F295.52, released on 10/20/2017), and by the Ethical Committee of the Italian Ministry of Health (approval No. 78/2018-PR, released on 02/05/2018). Briefly, male Wistar rats of 300–350 g body weight were fed a standard laboratory diet and water *ad libitum* in a controlled environment. Prior to surgery animals received 35 mg/kg body weight ketamine and 0.25 mg/kg body weight midazolam by intramuscular injection. Diffuse sTBI was induced according to the “weight drop” impact acceleration model of diffuse TBI devised by Marmarou et al. (1994). Briefly, rats were placed prone on a bed of specific polyurethane foam inserted in a special container; this foam dissipates the potential energy deriving from the mechanical forces and prevents any rebound of the animal after the impact that could produce spinal damages. A 450 g weight was dropped from a 2 m height onto the rat head that was protected by a helmet (metal disk previously fixed on the skull using dental cement) in order to uniformly distribute the mechanical force to the brain. Animals with skull fracture, seizures, nasal bleeding or that did not survive the impact (mortality rate of 7.7%) were excluded from the study. ILB^®^ was administered subcutaneously 30 min following the sTBI (Table 1), while animals were still under anesthesia. The ILB^®^ doses used encompass the human therapeutic dose of 1 mg/kg (used in EudraCT No 2017-005065-47) following simple allometric scaling. All animals surviving the impact survived for up to 7 days post-surgery required for the observational period. Sham operated animals (HC group) received anesthesia only. The sTBI group received no treatment following the head injury. At 7 days after sTBI induction, an *in vivo* craniectomy was performed in all animals during anesthesia. The rat skull was carefully removed, the brain was exposed, sharply cut along the sagittal fissure and the two hemispheres were separated. The hemispheres dedicated to biochemical analyses were freeze-clamped by aluminum tongues pre-cooled in liquid nitrogen and then immersed in liquid nitrogen. The freeze-clamping procedure was introduced to accelerate freezing of the tissue, thus minimizing potential metabolite loss. The remaining hemispheres, dedicated to gene expression analyses, were placed in 5–10 volumes of RNAlater^®^ Solution (Invitrogen Life Technologies, Loughborough, United Kingdom), an RNA stabilization solution that stabilises and protects RNA from degradation. Brain samples were stored at 4°C overnight to allow the solution to completely penetrate tissue. These samples were then prepared for gene expression analysis.

**TABLE 1:** Design of the sTBI study.

Group	Abbreviation	Treatment
1	HC	Anesthesia only (no sTBI)
2	sTBI	sTBI (as described above)
3	sTBI + ILB^®^ 1.0 mg/kg	sTBI followed by 1.0 mg/kg ILB^®^ (one injection)
4	sTBI + ILB^®^ 5.0 mg/kg	sTBI followed by 5.0 mg/kg ILB^®^ (one injection)
5	sTBI + ILB^®^ 15.0 mg/kg	sTBI followed by 15.0 mg/kg ILB^®^ (one injection)

### Gene expression analysis

Brain tissue in RNAlater^®^ solution were shipped to SourceBioscience within 2 weeks for RNA extraction and array analysis. The RNA extraction and gene expression analysis was performed by SourceBioscience using Agilent arrays. The expression data received from SourceBioscience were downloaded into separate files for each batch of samples. The background corrected signal was log2 transformed for all samples for statistical analysis. Based on the expression pattern of the control probes on each array, data was normalized to the β-actin probe (A_32_P137939; ACTB) for all arrays before analysis. Very low expression values (log_2_ <3.5), showing large within array variability, were removed from the analysis.

In the first instance a direct comparison was made between brain transcriptomics data gathered from the rat sTBI model and from sham operated animals to determine the impact of the injury at the gene expression level. The effect of ILB^®^ was determined from the comparison of gene expression in sTBI animals with and without treatment. The expression data were analysed using the MetaboAnalyst 5.0 program (MetaboAnalyst, CA, United States, Xia and Wishart, 2016) to identify differentially regulated genes. A *p* value < 0.05 and expression change >10% was regarded as significant. The differentially regulated genes and the logFC (log of fold change) were uploaded to the Ingenuity pathway analysis platform (QIAGEN IPA, QIAGEN Inc., https://digitalinsights.qiagen.com/IPA) for further analysis. Based on the up and downregulation of the genes, the Ingenuity platform assigns activation/inhibition z-scores (*p* value) to different molecular pathways affected. It also identifies the activation/inhibition pattern of upstream regulators to these pathways. These upstream regulators can either represent biological molecules functionally affected by the treatment (growth factors, cytokines, receptors, etc.) or can identify drugs with known mechanism of action that could bring about the effects seen in the system. Additionally, the Ingenuity knowledge base was used to analyse the impact of gene expression changes seen in human neurodegenerative conditions such as ALS and AD on molecular pathways significantly affected by the disease process for comparison with the impact of sTBI on the brain.

### Pharmacokinetic studies in rodents

ILB^®^ was administered intravenously or subcutaneously to mice (DBA/2JRccHsd, Harlan, Holland) and rats (Sprague-Dawley, Harlan, Holland) (studies carried out by Redoxis AB, Lund, Sweden). Serum HGF levels were measured by ELISA assay (R&D Systems, Abingdon, United Kingdom) according to the manufacturer’s instructions at different time points following drug administration.

### Clinical studies in healthy volunteers and patients with ALS

The clinical studies were conducted at the Sahlgrenska University Hospital in compliance with the defined protocol, the regulatory requirements, Good Clinical Practice (GCP) and the ethical principles of the Declaration of Helsinki and was approved by the Medical Products Agency (EudraCT No 2011-004111-23 and EudraCT No 2017-005065-47). All subjects received written and verbal information concerning the study prior to the start of any study-related procedures and gave written informed consent to their participation.

The study group in EudraCT No 2011-004111-23 comprised 15 male subjects, 18–50 years of age, healthy according to their medical history, including normal bleeding, diathesis, anamnesis and with no clinically relevant observations for vital signs or deviations in physical examination and laboratory tests. Blood was collected from each subject in sodium citrate blood collection tubes at different time points before and after a single infusion of between 3 and 15 mg/kg ILB^®^ (n = 3/dose) and stored at −80°C until analysis.

The study group in EudraCT No 2017-005065-47, comprised 13 patients with ALS (see detailed description in Logan et al., 2022). Blood was collected from each subject in sodium citrate blood collection tubes at different time points before and after a subcutaneous injection of ILB^®^ on the first and last episode of the study and stored at −80°C until analysis.

### Measurement of serum growth factors in human participants

Serum HGF and BDNF measurements were made with commercial HGF and BDNF ELISA kits, both from R&D Systems, according to the manufacturer’s instructions by the Clinical Chemistry Laboratory at the Sahlgrenska University Hospital (Gothenburg, Sweden).

## Results

### ILB^®^ competes with the interaction between Glypican-3 and HGF

**FIGURE 2:**
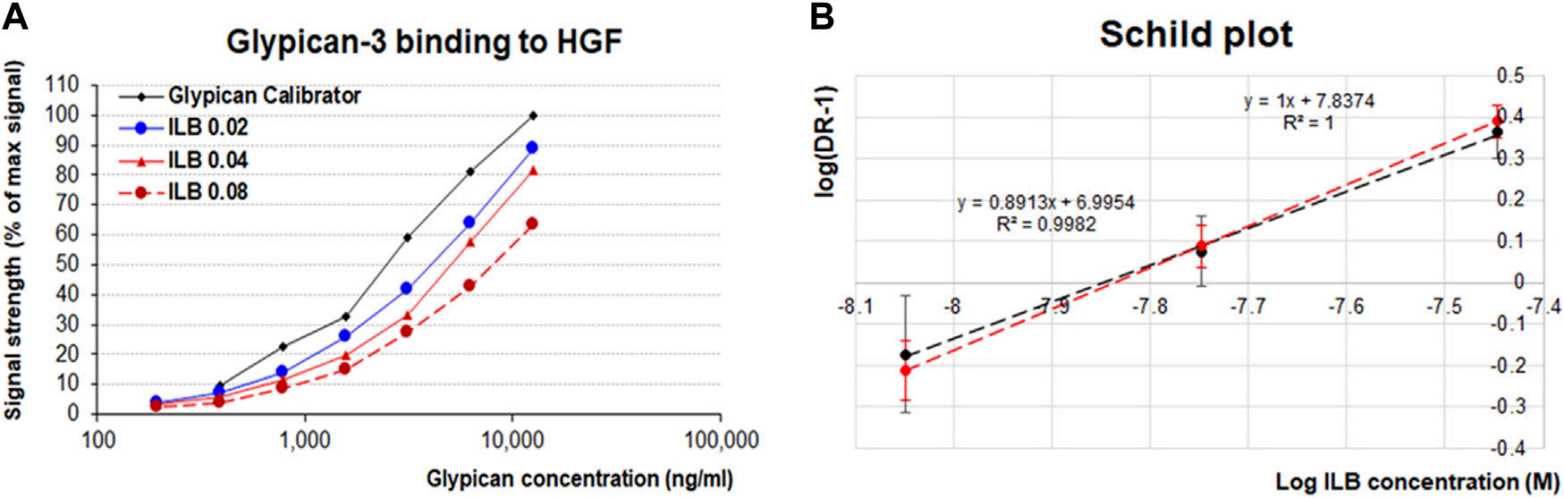
The effect of ILB^®^ on Glypican-3:HGF binding. **(A)** Concentration-effect curves of Glypican-3 binding to HGF in the absence or presence of various concentrations of ILB^®^ (µg/ml). **(B)**. Regression analysis of Log (DR-1) values for different ILB^®^ concentrations results in a linear regression with a slope not significantly different from 1 (with the given variability of the assay—see in Supplementary Material S1).

The ELISA assay was designed to measure the binding of Glypican-3 to HGF *ex vivo*. The assay has been validated using stringent quality control (Supplementary Material S1) to allow the accurate quantification of binding. The addition of increasing concentrations of ILB^®^ to the binding assay elicited a shift to the right in the Glypican-3-HGF concentration-dependent binding curve (Figure 2A). The quantification of Glypican-3 binding allows the dose ratio (DR) calculation of the Glypican-3 concentration required to elicit a 50% signal in the assay (EC_50_) in the absence or presence of ILB^®^. The DRs from the experiments with increasing ILB^®^ concentrations (average Mn = 2241Da for the ILB^®^ batch used for all experiments) allowed construction of a Schild plot (Figure 2B). The arising affinity of ILB^®^ in these experiments was revealed as a pA_2_ = 7.84 with a slope around unity indicative of a simple competitive effect of ILB^®^ to inhibit the interaction between Glypican-3 and HGF.

### ILB^®^ has mild anticoagulant activity

**FIGURE 3:**
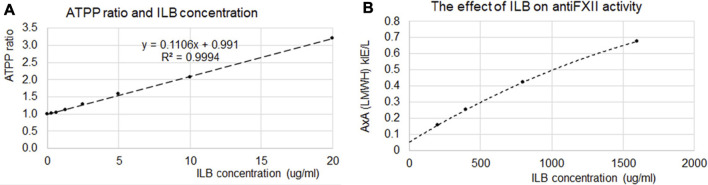
Anticoagulant effect of ILB^®^ measured by APTT and anti-FXII activity. **(A)** At relatively low concentrations ILB^®^ increases the Activated Partial Thromboplastin Time (APTT), yet **(B)** higher ILB^®^ concentrations are required to impact anti-FXII activity.

The heparin-like structure of ILB^®^ warranted analysis of the potential anticoagulant action. *Ex vivo* analysis using normal human serum spiked with variable amounts of ILB^®^ demonstrated that the drug had a modest effect on Activated Partial Thromboplastin Time (APTT) while higher ILB^®^ concentrations were required to modify anti-FXII activity (Figure 3).

### ILB^®^ modifies intracellular signaling of heparin-binding growth factors

**FIGURE 4:**
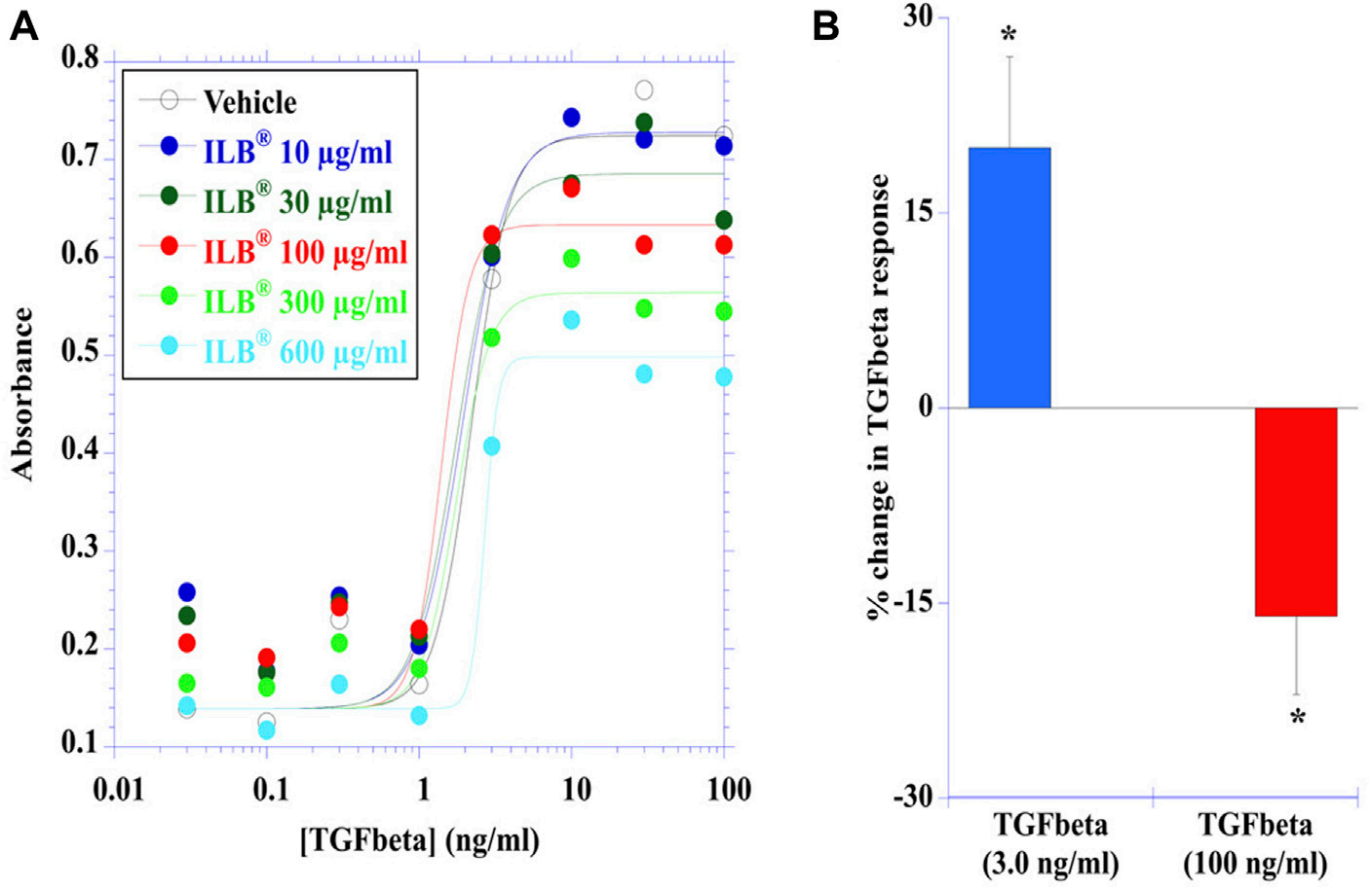
ILB^®^ impacts the intracellular signalling of heparin-binding growth factors. **(A)** Concentration-dependent TGFß-induced responses in human embryonic kidney cells (HEK-Blue™) stably transfected with human TGFßR1, Smad3 and Smad4 genes, along with Smad3/4-binding elements (SBE)-inducible secreted embryonic alkaline phosphatase (SEAP) reporter gene were differentially modulated by ILB^®^ in a concentration-dependant manner over a range of concentrations (10–600 μg/ml). The data presented was pooled from 7 independent experiments. **(B)** The potentiation of the TGFß response (illustrated at 3.0 ng/ml) by ILB^®^ at 10 μg/ml (*p < 0.05; mean + SEM, n = 7) and the inhibition of the TGFß (100 ng/ml) response by ILB^®^ at 600 μg/ml (*p < 0.05; mean - SEM, n = 7).

TGFβ-induced a concentration-dependent stimulation of HEK-Blue TGFβ reporter cells evident by activation of the TGFβ/Smad signaling pathway leading to the formation of a Smad3/Smad4 heterocomplex that entered the cell nucleus binding to SBE sites to induce the production of SEAP as quantified with QUANTI-Blue™ Solution. Effective concentrations of TGFβ were in the low ng/ml concentration range (Figure 4). ILB^®^ altered the TGFβ-induced response in a concentration-dependent manner (Figure 4A); Figure 4B shows that at the lower concentrations of ILB^®^ tested there was an increase in the TGFβ-induced response (3.0 ng/ml TGFβ response potentiated by 10 μg/ml ILB^®^ by 20 ± 7%, mean ± SEM, n = 7, *p* < 0.05, Mann Whitney *U* test), yet at higher concentrations of ILB^®^ inhibition of the TGFβ-induced response was revealed (100 ng/ml TGFβ was inhibited by 600 μg/ml ILB^®^ by 16 ± 6%, mean ± SEM, n = 7; *p* < 0.05, Mann Whitney *U* test).

In the same seven independent experiments, low molecular weight (LMW) heparin was investigated for a side-by-side evaluation in comparison with ILB^®^. LMW heparin also altered the TGFβ-induced response in a concentration-dependent manner (data not shown), although the effects were not as clear cut as those with ILB^®^. Thus, LMW heparin at the lower concentrations tested tended overall to increase the TGFβ-induced response to sub-maximal concentrations of TGFβ (*p* < 0.05; Mann Whitney *U* test). At the higher concentrations of TGFβ tested with higher concentrations of LMW heparin, the inhibition of the TGFβ-induced response did not reach statistical significance.

### ILB^®^ is an immunomodulator, suppressing inflammatory cytokines

**FIGURE 5:**
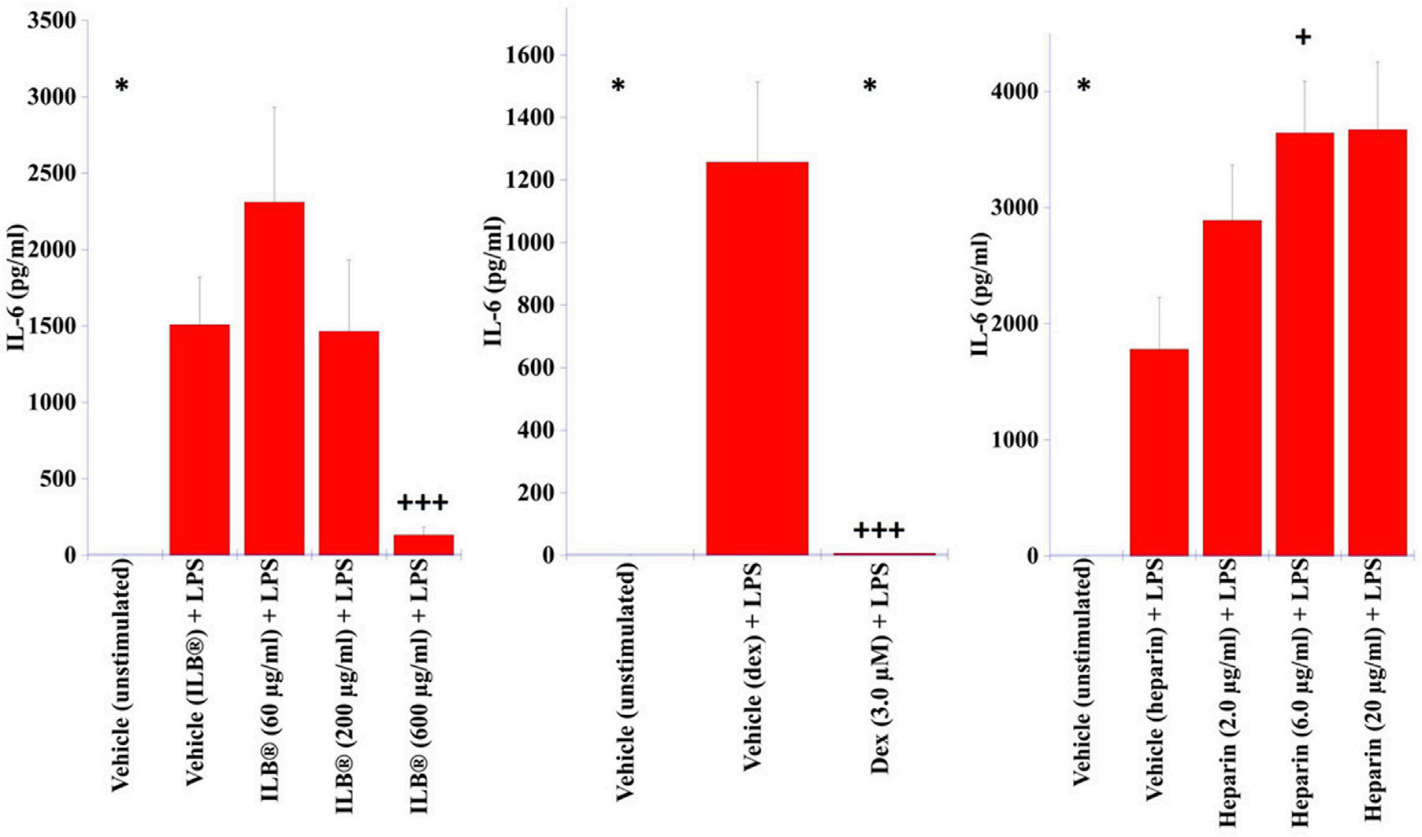
Impact of ILB^®^ upon the secretion of IL-6 from human monocytes. Monocytes purified from human PBMCs were cultured in the absence of stimulation (media) or stimulated with LPS (0.01 ng/ml) in the absence (Vehicle) or presence of either ILB^®^, dexamethasone or heparin for 24 h. Levels of IL-6 were quantified in the cell culture supernatant by ELISA. Data presented as mean + SEM, n = 10. * indicates IL-6 levels below the limit of detection from monocytes from at least one donor (5.0 pg/ml). +*p* < 0.05, +++*p* < 0.001 Significant difference to stimulation (Mann Whitney *U* Test).

Stimulation of human purified monocytes with LPS (0.01 ng/ml) for 24 h increased secretion of IL-6 that was prevented by co-application of ILB^®^ or dexamethasone but not heparin (Figure 5).

### ILB^®^ modulates gene transcription in cultured human neuronal and glial cells

Functional analysis of the therapeutically relevant concentrations of ILB^®^ revealed the induction of profound expression changes in pivotal growth signaling pathways affecting molecular and cellular functions such as cell survival, cell differentiation, cell division and microtubule dynamics (Supplementary Tables S1–S4). Upstream regulator analysis indicates that the changes induced by ILB^®^ were the result of enhanced activation of growth factors such as HGF, BDNF and VEGF, among others (Supplementary Tables S5, S6). Naturally in *in vitro* experiments the growth factor signaling that can be activated depends on the growth factors present in the system. The dose-response analysis in both cell types indicated that, while there was a dose dependent strengthening of the signal up to 1 μg/ml ILB^®^, increasing the ILB^®^ concentration to 10 μg/ml did not lead to further increase in the response.

### ILB^®^ induces profound transcriptional changes in the brain after sTBI that impact pathways also compromised in other neurodegenerative conditions

In the first instance we found it was important to confirm that the sTBI model indeed affects the same molecular pathways as chronic neurodegenerative conditions, such as ALS and AD. The genes differentially deregulated in ALS and AD were downloaded from the Ingenuity knowledge base (a curated data repository based on published literature; QIAGEN Inc., https://digitalinsights.qiagen.com/IPA). The genes significantly deregulated by sTBI affect the function of large canonical pathways regulating axonal guidance, neuroinflammation, LTP, glutamate signaling and the signaling pathway of several growth factors and cytokines (full set of data in Supplementary Tables S7, S8). The cellular functions affected by sTBI include neuronal death, neurotransmission, neuronal development, vasculogenesis and microtubule dynamics, among others (full set of data in Supplementary Table S9). The diseases associated with the differential gene expression seen in sTBI include ALS, cerebrovascular dysfunction, cognitive impairment, mood disorders and movement disorders (full set of data in Supplementary Tables S9, S10).

**FIGURE 6:**
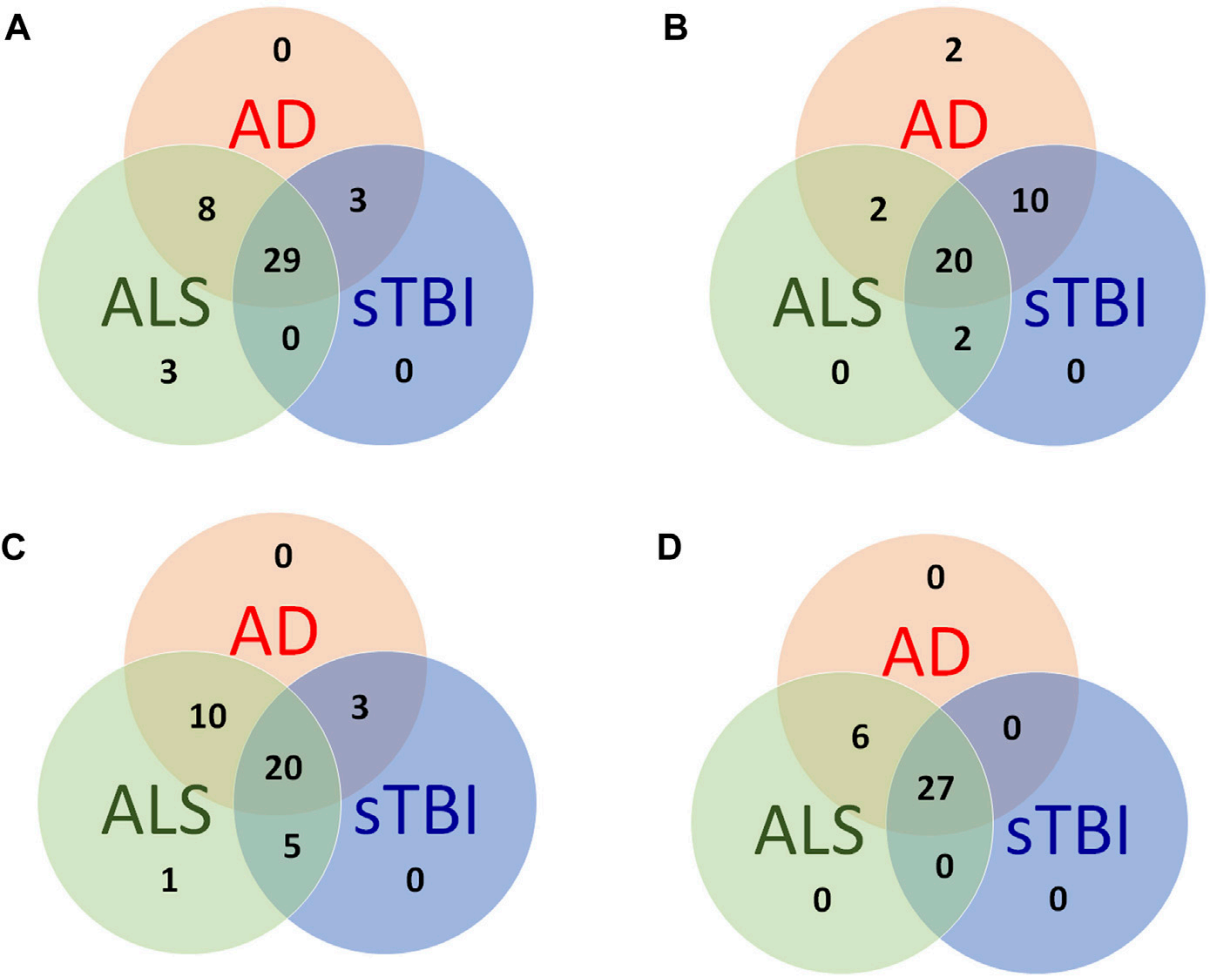
Similarities and differences between the functional signature of human neurodegenerative conditions and the sTBI model. **(A)** Overlap of the top 20 significantly affected canonical pathways. **(B)** Overlap of the top 20 significantly affected functions. **(C)** Overlap of the top 20 significantly affected diseases. **(D)** Overlap of the top 20 upstream regulator growth factors and cytokines. The complete dataset from the brain transcriptome analysis is available on application to the authors.

The comparison of these pathways and functions confirmed that the molecular and functional pathways affected by sTBI resemble those affected in neurodegenerative conditions such as ALS and AD (Figures 6A–C). The deep understanding of molecular interactions afforded by such analyses allows the prediction of upstream regulators that could be responsible for gene expression changes. As such the methodology is used for target identification, but also allows the comparison of potential therapeutic targets between human diseases conditions and animal models.

The effects of ILB^®^ on gene expression was investigated using the same methodology and approach as described above. The activation z scores calculated by IPA (Qiagen Ingenuity pathway analysis; QIAGEN Inc., https://digitalinsights.qiagen.com/IPA), when available, allow the visualization of the functional consequences of gene expression changes in the system. The comparison of activation z scores of different pathways and molecular functions indicate that ILB^®^ reverses the deregulation of molecular pathways and functions in the sTBI model (Table 4 and Table 5; detailed data in Supplementary Tables S11, S12). Furthermore, this effect of ILB^®^ is associated with significant changes in the activation status of growth factors such as VEGF, BDNF, HGF, FGF and EGF (Supplementary Table S13). It is also clear, especially from the growth factor activation patterns, that the effect of ILB^®^ does not follow a linear dose-response curve. Most effects appear strongly at 1 mg/kg and reach their maximum effect at 5 mg/kg. Increasing the dose to 15 mg/kg does not increase the effect of the drug.

In summary, the gene expression changes seen in the animal model of sTBI indicate that the administration of ILB^®^ after sTBI reverses many of the adverse effects of sTBI on functional molecular pathways by modulating the activity of growth factors and cytokines. Of particular relevance to many neurodegenerative diseases, these pathways include glutamate signaling, mitochondrial function and inflammation as shown in Supplementary Figures S1–S3.

### ILB^®^ evokes the pulse release of heparin-binding growth factors into the circulation of rodents

**FIGURE 7:**
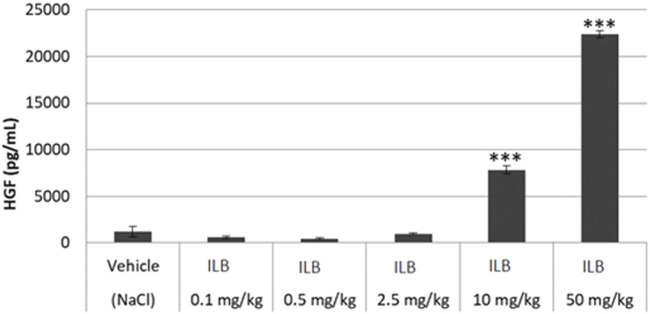
The HGF concentration in mouse peripheral blood measured 30 min after ILB^®^ administration. Mean ± SEM is shown as well as statistically significant data compared to vehicle (student t-test, **p < 0.01, ***p < 0.001).

**FIGURE 8:**
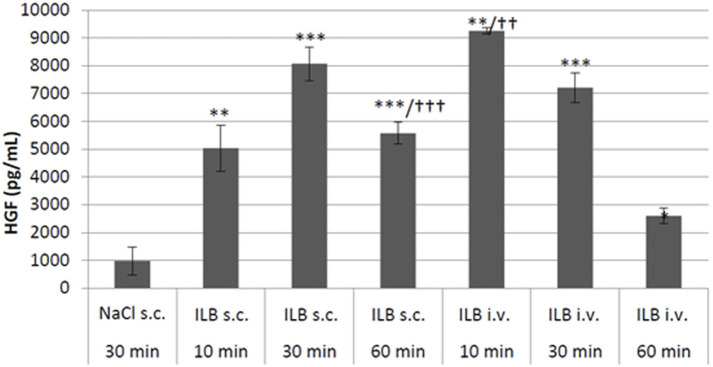
The HGF concentration was measured in mouse peripheral blood at 10, 30 and 60 min after either subcutaneous (s.c.) or intravenous (i.v.) administration of 10 mg/kg ILB^®^. Mean ± SEM is shown as well as statistically significant increases compared to vehicle (student t-test, *p < 0.05, **p < 0.01, ***p < 0.001) and significant differences between the two routes of administration (student t-test, ^††^ *p* < 0.01, ^†††^ *p* < 0.001).

To confirm the contention that ILB^®^ leads to the release of heparin binding growth factors into the circulation from the endothelial bed, HGF was measured in the plasma of mice after ILB^®^ subcutaneous (s.c.) or intravenous (i.v.) administration. The administration of ILB^®^ increased the plasma level of HGF in a dose-dependent fashion after administration of 0.1–50 mg/kg ILB^®^ (Figure 7). Increased HGF is also evident in rats after ILB^®^ administration (data not shown). When 10 mg/kg ILB^®^ was administered s. c. or i. v. and blood collected after 10, 30 and 60 min, the highest levels of HGF were detected after 10 min for i. v. Whereas for s. c. injection the peak level occurred at 30 min (Figure 8). Raised levels of HGF were detected at all post-administration time-points measured with levels significantly increased compared to untreated animals.

### ILB^®^ evokes the pulse release of heparin-binding growth factors into the circulation of humans

In clinical studies with ILB^®^ (EudraCT No 2011-004111-23) infusion of 3 or 6 mg/kg in healthy human volunteers induced a rapid bolus release of physiologically relevant titers of heparin-binding growth factors, including HGF and BDNF, into the circulation allowing redistribution of these tissue repair signals (Table 2).

**TABLE 2:** HGF and BDNF levels in the blood plasma of healthy humans measured by ELISA before and after infusion of ILB^®.^

Growth factor	Baseline plasma level (pg/ml)	Max plasma level (pg/ml)	Time to max (mins)
HGF (6 mg/kg ILB^®^)	1,167 ± 503	50,333 ± 8,083	60
BDNF (3 mg/kg ILB^®^)	266 ± 155	828 ± 409	180

**FIGURE 9:**
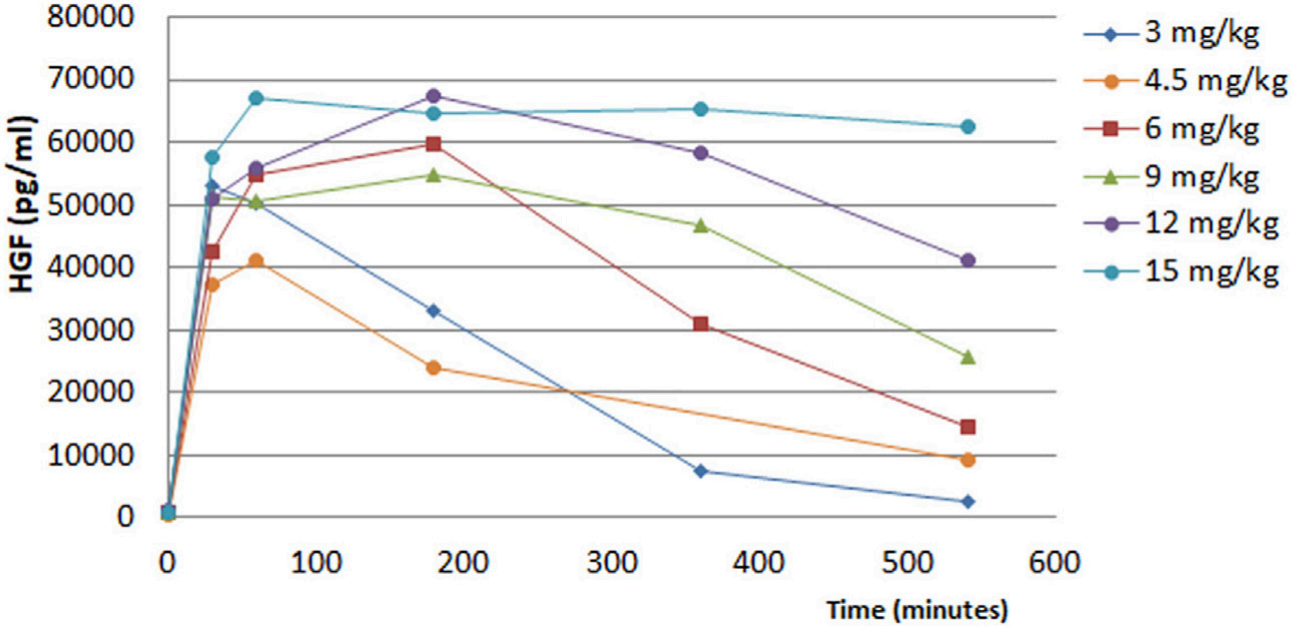
Time course of HGF release into the plasma of healthy humans after ILB^®^ administration.

The pharmacokinetics (PK) of the HGF release from endothelial cells in humans was seen to be similar to that seen in animals. Following i. v. injection of ILB^®^, release of HGF from endothelial cells was rapid and ILB^®^ dose-dependent (Figure 9). The rapid rise of HGF levels after the bolus injection of ILB^®^ was apparent within 30 min. The subsequent decline of plasma HGF mirrored the decline of ILB^®^ concentration in the plasma. This dose-dependent HGF release had a ceiling effect, with its maximum showing variability between individuals.

**FIGURE 10:**
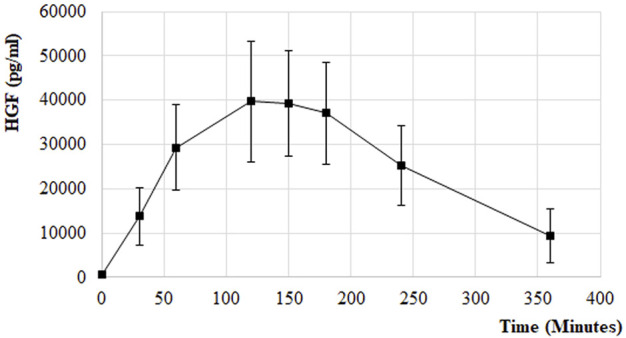
HGF release following s. c. injection of 1 mg/kg ILB^®^ in patients with ALS. PK of HGF release in response to ILB^®^ administration. Error bars represent the standard deviation.

**FIGURE 11:**
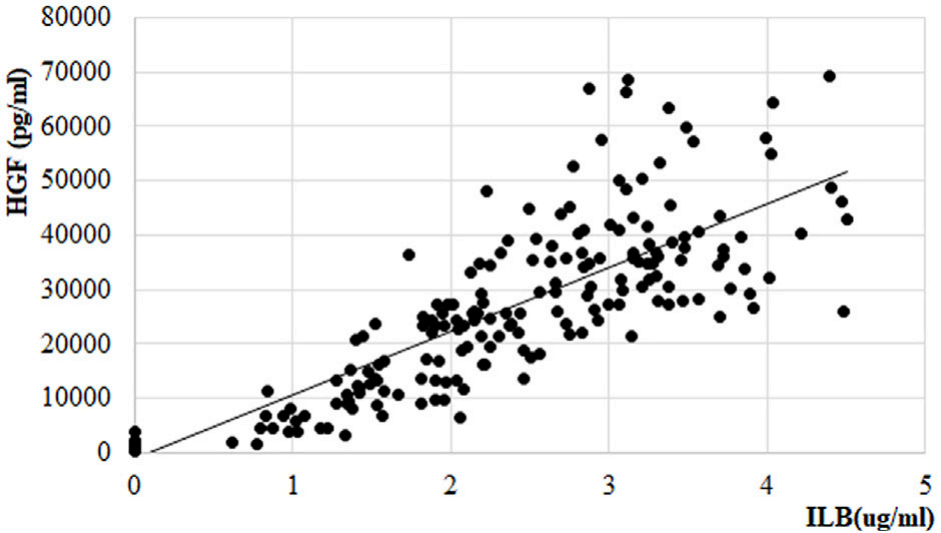
Relationship between HGF and ILB^®^ concentration in plasma of patients with ALS. Linear regression within therapeutic range of ILB^®^, regression function: y = 11733x-1197.2; *R* ^2^ = 0.7014.

HGF release was also measured from the PK samples at the first and the last treatment session in the EudraCT No 2017-005065-47 clinical trial of ILB^®^ in patients with ALS. Following s. c. administration of 1 mg/kg ILB^®^, the HGF peak plasma concentration reached an average 60-fold increase in patients (Figure 10). The relationship between average HGF and ILB^®^ levels was strongly linear. At an individual level, there was some variability between ILB^®^ plasma concentration and HGF release (Figure 11). It was also clear that the s. c. administration of 1 mg/kg ILB^®^ released HGF near to the maximum levels observed with higher ILB^®^ concentrations.

**FIGURE 12:**
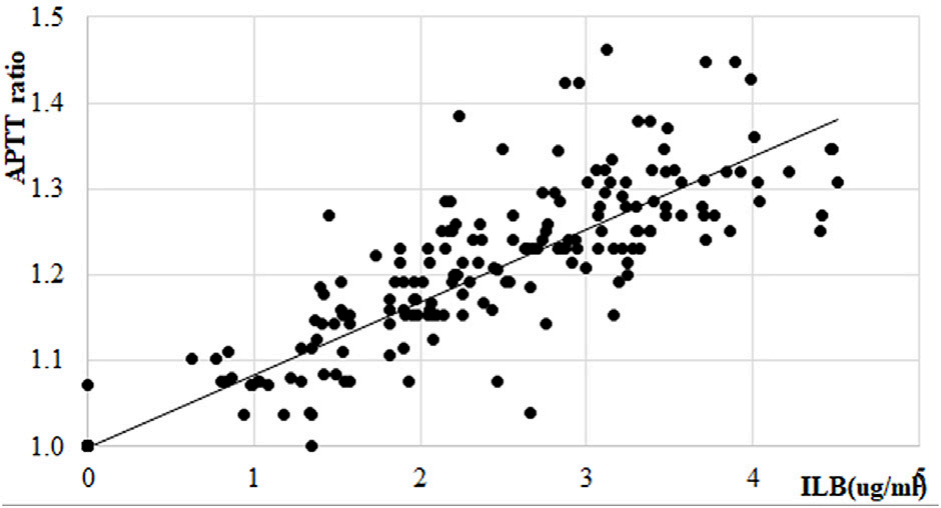
Effect of ILB^®^ on APTT ratio in patients with ALS. Linear regression within therapeutic range of ILB^®^, regression function: y = 0.0844x+0.9999; *R* ^2^ = 0.5202.

In the ALS trial EudraCT No EudraCT No 2017-005065-47 [full clinical results reported in Logan et al. (2022)] the increase in the APTT was measured as part of the safety assessment of the drug. ILB^®^ plasma concentrations had a significant and strong relationship with the change in APTT time in individual patients (Figure 12).

In summary, therapeutic levels of ILB^®^ elicited a steady dose-dependent change in the APTT time and in HGF release assessed in the plasma from all patients. This release most likely affects all heparin-binding growth factors and allows the redistribution of these factors to their binding sites.

## Discussion

The aim of this study was to evaluate the mechanism of action of the novel neuroprotective LMW dextran sulphate reported to have a beneficial effect in ALS (Logan et al., 2022). The working hypothesis was that ILB^®^ binds to and affects the activity of heparin-binding growth factors and cytokines.

The *in vitro* data presented provide evidence that ILB^®^ binds to heparin-binding proteins (effect on the coagulation system and HGF-Glypican3 binding). We have also demonstrated *in vitro* that ILB^®^ enhances the effect of heparin binding growth factors in the system with profound effects on gene expression. The animal and human studies performed indicate that the ILB^®^-induced release of growth factors does occur *in vivo*. Additionally, our studies in sTBI animals indicate that the growth factor release and redistribution initiated by ILB^®^ is followed by significant gene expression changes *in vivo* that correct dysregulated mitochondrial function, limit oxidative stress, attenuate glutamate toxicity, and control inflammation.

**FIGURE 13:**
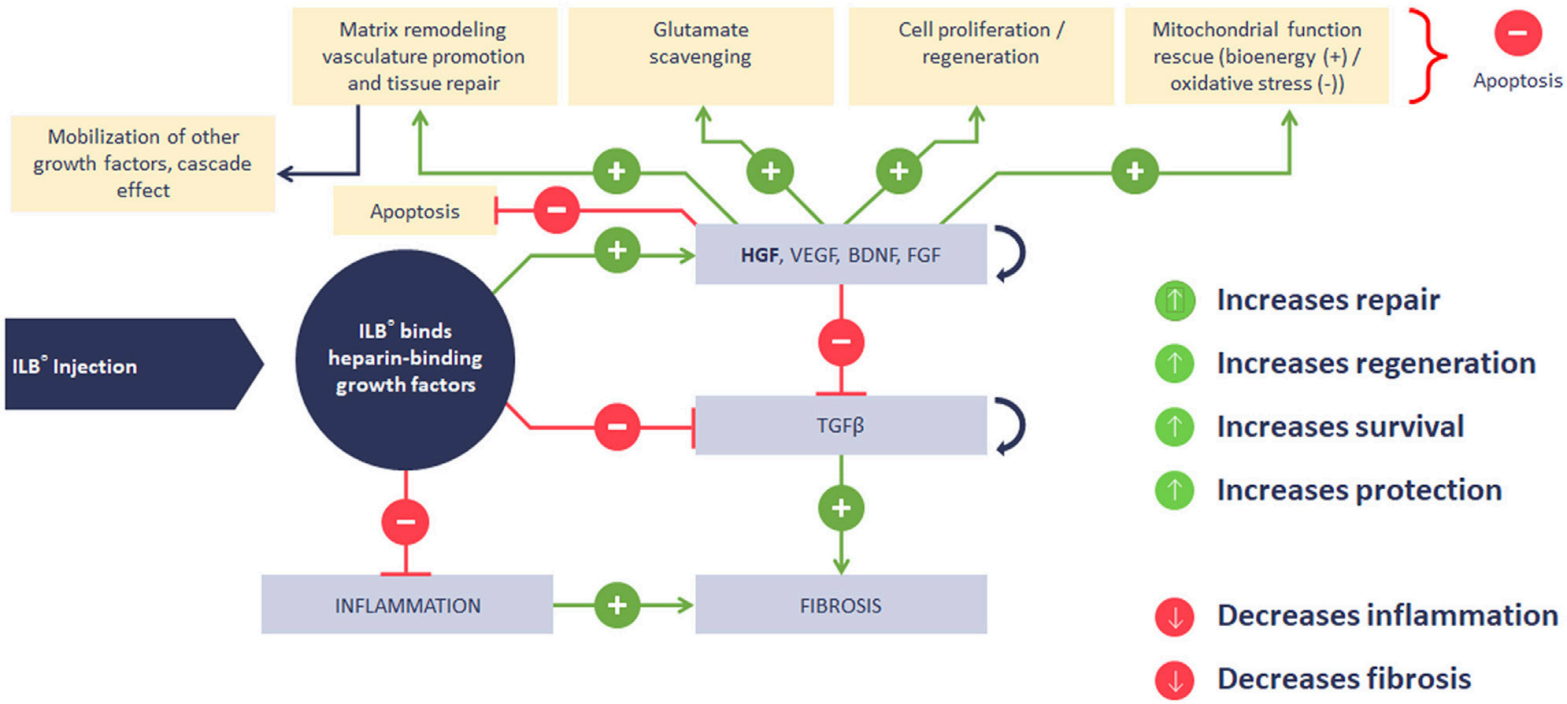
Summary diagram of ILB^®^ mechanism of action.

We have also justified our exploitation of a rodent model of sTBI to evaluate the potential functional impact of these disease-relevant mechanisms of action of ILB^®^. Figure 13 illustrates the sequential molecular and cellular mechanisms of action of ILB^®^ that lead to the beneficial functional effect seen in our neurodegeneration model, a mechanistic sequence that is further explained in the paragraphs set out below.

### ILB^®^ releases, redistributes and modulates endogenous heparin binding growth factors and cytokines with profound downstream effects

The demonstration of ILB^®^-stimulated release of heparin-binding growth factors, including HGF and BDNF, reported here in healthy humans and in people with ALS is consistent with previous reports (Logan et al., 2022). This activity is probably related to the known ability of related glycosaminoglycans to bind and solubilize growth factors, releasing them from their extracellular stores in the endothelium, although the pharmacokinetics and profile of growth factor release is unique to each glycosaminoglycan tested (Barritault et al., 2017; Zhang et al., 2019). Furthermore, the observation of heparin binding growth factor release and redistribution is also supported by the transcriptomic signature of ILB^®^, that indicates an activation, without changes in expression, of several growth factors and cytokines in an animal model of sTBI. The *ex vivo* effect of ILB^®^ on Glypican-2-HGF binding, modulation of human monocyte cytokine secretion and its effect on APTT provide direct evidence supporting this hypothesis for the ILB^®^ mechanism of action.

HGF is a potent endogenous neurotrophic and myogenic factor synthesized by epithelial and endothelial cells and stored in the endothelial extracellular matrix of peripheral and central tissues. Once released into the circulation and redistributed, this versatile growth factor acts directly through the MET receptor to protect and repair motor neurons and muscle cells from damaging signals and also initiates indirect repair responses in macrophages and glia [reviewed by Desole et al. (2021)]. HGF stimulates multi-faceted cellular processes, including glucose transport and metabolism and also reduces inflammation, oxidative stress and glutamatergic neurotoxicity whilst promoting central and peripheral synaptogenesis and neuroplasticity (Perdomo et al., 2008; Lee et al., 2019; Molokotina et al., 2019; Molnarfi et al., 2019; Sun et al., 2002; Desole et al., 2021). Interestingly, the therapeutic potential of recombinant and vector delivered HGF for ALS is being tested in clinical trials (Sufit et al., 2002; Sun et al., 2002; Warita et al., 2019) that, thus far, have demonstrated safety and tolerability.

BDNF is one of the most studied neurotrophic factors, with impressive neuroprotective credentials (Numakawa et al., 2018; Colucci-D'Amato et al., 2020). As well as the brain, BDNF is expressed in the endothelium, from where the raised circulating levels we have observed after ILB^®^ treatment probably originate (Brigadski and Leßmann, 2020). Reduced levels of BDNF have been implicated in the pathogenesis of ALS (Pradhan et al., 2019). The potential of BDNF to treat a range of neurodegenerative conditions including ALS has long been highlighted. However, clinical trials with recombinant BDNF in patients with ALS have yielded disappointing results that have been attributed to the poor pharmacokinetics and pharmacodynamics of delivered exogenous BDNF (Colucci-D'Amato et al., 2020). The observation that ILB^®^ can mobilize and redistribute endogenous stores of BDNF alongside HGF, making their combined neuroprotective activity available to compromised neurons, glia and muscle cells, is one that holds great promise for people with ALS and other neurodegenerative conditions. The protective cellular responses that we have observed with ILB^®^ treatment after sTBI, including normalization of mitochondrial function, reduction in oxidative stress, suppression of glutamate excitotoxicity and attenuation of pro-inflammatory signaling are all in accord with the known activities of HGF and BDNF and are directly relevant to the pathogenesis of ALS and other neurodegenerative conditions.

The *in vitro* studies reported here offer evidence that ILB^®^ not only releases and redistributes but also impacts on the interaction of heparin-binding growth factors and cytokines with their receptors. The results from the HEK-Blue TGFβ reporter cells reporter assay revealed that the functional impact of ILB^®^ on growth factors and cytokines is quite sophisticated. For example, there is a potentiation of TGFβ responses when human cells are exposed to low concentrations of TGFβ together with low concentrations of ILB^®^; such concentrations that are achieved in clinical studies with ILB^®^ in healthy volunteers. Yet at high concentrations of TGFβ with higher concentrations of ILB^®^ the cytokine potentiation is reversed into an inhibition. Given the non-linearity of many growth factor signaling mechanisms (Calabrese and Baldwin, 2002; Calabrese and Mattson, 2017) this is not unexpected. Indeed, the non-linear dose response to ILB^®^ seen in the sTBI animal model supports the notion that the same mechanism is at play *in vivo*. Furthermore, the studies in humans showing the impact of ILB^®^ upon circulating HGF levels indicate that lower levels of ILB^®^ (that elicit a pulsed release of HGF) given at intervals are more likely to have a beneficial effect than high or sustained levels of ILB^®^ that may sequester HGF in the plasma for prolonged periods of time, an effect that presumably would lower physiological responses to the growth factor.

### ILB^®^ corrects mitochondrial dysfunction and reduces oxidative stress

Obrador et al. (2021) have described the link between oxidative stress, redox status, bioenergetics and mitochondria in the pathophysiology of ALS. Mitochondrial dysfunction is not specific for ALS but appears to be a feature of several neurodegenerative diseases. The mitochondria, besides being the powerhouses of the cell by producing adenosine triphosphate (ATP), are also involved in other vital functions of the cell. They participate in the production of key metabolites of the cell, regulate apoptosis and calcium buffering and are the primary source of endogenous reactive oxygen species. The high energy consumption and the lack of energy storage makes neurons particularly vulnerable to mitochondrial dysfunction. Several studies have found mitochondrial aggregates in the muscles and spinal motor neurons of patients with ALS (Zuo et al., 2021; Nesci et al., 2022). Oxidative stress is one of the most important factors involved in neuronal ageing and death and several studies have documented an increased oxidative stress in post-mortem tissue of patients with ALS compared to healthy individuals (summarized in the review by Obrador et al., 2021).

We have previously demonstrated that ILB^®^ normalizes the metabolic profile of the rat brain after sTBI (Lazzarino et al., 2020; Lazzarino et al., 2022) and also the dysregulated metabolites found in the plasma of people with ALS (Lazzarino et al., 2021). The cerebral transcriptional data reported here from the sTBI model strongly support the conclusion that ILB^®^ restores mitochondrial function and reduces the consequences of oxidative stress. For example, the observation of ILB^®^ dependent activation of defense pathways such as neuronal CREB and cAMP signalling and several antioxidant mechanisms (Bhatti et al., 2017; Yoo et al., 2017; Bouchez and Devin, 2019; Signorile et al., 2020) indicates restoration of cellular homeostasis.

### ILB^®^ limits glutamate excitotoxicity

The metabolomics signature of ALS reflects partly the neuronal death and gliosis and partly the pathophysiological mechanisms that drive the disease (Kumar et al., 2013; Blasco et al., 2018; Lanznaster et al., 2018; Goutman et al., 2020). Glutamate is the most often cited metabolite found to be increased in patients with ALS and other neurodegenerative conditions (Blasco et al., 2014). Astrocytes control and reduce the concentrations of extracellular glutamate and hence the increase in this metabolite seen in patients with ALS most likely arises from the decreased glutamate uptake by astrocytes in these patients (Blasco et al., 2014). The uptake of glutamate depends on the functioning of specialized glutamate transporters (EAAT2) in these cells. Excessive glutamate in the neuronal environment leads to excessive firing via predominantly ionotropic glutamate receptors and increased [Ca^2+^]_i_ from influx and release from calcium stores with excessive levels being neurotoxic, leading to cell death. There is evidence the deficits in glutamate uptake by astrocytes in patients with ALS is due to the loss or mutation of the EAAT2 transporter (Jackson et al., 1999).

The prevention of raised brain glutamate levels by ILB^®^ after sTBI (Lazzarino et al., 2020; Lazzarino et al., 2022) and the gene expression data obtained from rat brains after sTBI reported here suggest an important and distinctive influence of the drug on glutamate signaling and related excitotoxicity. Clearly the observed effect of ILB^®^ on normalizing the glutamate signaling pathway through enhancing levels of glial glutamate transporters without affecting neuronal glutamate signaling indicates that, while the drug is able to moderate the sTBI induced excitotoxicity (and reduce neuronal death), it does not interfere with the most important neuronal functions of glutamate as a neurotransmitter that promotes synaptic remodeling.

### ILB^®^ attenuates inflammation

The mobilization of neuroinflammatory processes is a common event in many neurodegenerative disorders where the death of neurons leads to the activation of microglia and astrocytes that further fuel neurodegeneration (Haukedal and Freude 2019; Rodrigues Lima-Junior et al., 2021). Post-mortem studies have found microglial activation and higher concentrations of pro-inflammatory mediators in patients with ALS (Quek et al., 2022). Furthermore, co-cultures of astrocytes from patients with fALS and sALS are toxic to motor neurons due to the upregulation pro-inflammatory genes (Mancuso and Navarro, 2015). As the disease progresses, central infiltration of inflammatory cells from the periphery exacerbates the neuroinflammatory processes (Dressman and Elyaman, 2021).

The present studies have demonstrated the anti-inflammatory activity of ILB^®^ that is achieved by the modulation of several molecular and cellular responses. The data obtained from the HEK-Blue TGFβ reporter cells demonstrates that ILB^®^ directly modulates intracellular signaling by the pleiotropic cytokine TGFβ. The anti-inflammatory activity of ILB^®^ is further supported by the reduction in secretion of key inflammatory cytokines (e.g., IL-6) from monocytes (myeloid cousins of microglia) that further drive central as well as peripheral inflammation. Furthermore, the transcriptomic signature of ILB^®^ supports a strong effect on inflammatory processes.

### sTBI is a valid model of neurodegenerative disease that demonstrates the functional benefit of ILB^®^ treatment

The experimental models available to help evaluate the mechanism of action and potential efficacy of new drugs like ILB^®^ to treat heterogenous neurodegenerative diseases like ALS all have limitations. Most focus on modeling the effect of rare genetic mutations that are of limited relevance to people suffering from the common sporadic forms of the disease (Liguori et al., 2021). This may explain in part why few of the drugs effective in the transgenic mouse models of these diseases have translated their potential in the clinic.

The sTBI model, by contrast, recapitulates the most common pathogenic processes involved in many sporadic heterogeneous neurodegenerative diseases, including ALS. The sTBI model shows perturbation of neurotransmission, synaptic remodeling, neuronal survival, inflammation and oxidative stress. Additionally, many of the predicted upstream regulators of disease progression (possible drug targets) identified in the sTBI model are relevant to those involved in these diseases. Our analysis, similar to recent reports from other groups (Rana et al., 2019 and, 2020), supports a predictive potential of therapeutic drug action for neurodegenerative diseases using the sTBI model. Positive results from our recent open label Phase II trial of ILB^®^ with patients with ALS (Logan et al., 2022) support our assertion that the sTBI model recapitulates the common mechanisms involved in sporadic neurodegenerative diseases and can be used to predict therapeutic potential of drugs. We recognize that more extensive and broader characterization of the model is still required before its wider acceptance. Nevertheless, the sTBI model has allowed us to investigate the mechanism of action of ILB^®^ and the exploration of its potential to modulate the pathogenic mechanisms involved in neurodegenerative diseases.

The profound transcriptional changes observed in the traumatized brain after ILB^®^ treatment are indicative of the beneficial impact of the drug on neuronal function. The functional effects of ILB^®^ in the brain are widespread, impacting multiple cellular processes relating to neuronal death, neurotransmission, neuronal development, synaptogenesis, vasculogenesis, microtubule dynamics and many other pathophysiological responses. These effects are accompanied by clear and measurable neurocognitive changes in these animals (Lazzarino et al., 2022).

### ILB^®^ has potential as a disease modifying drug to treat ALS

As with most neurodegenerative conditions, the pathogenesis of ALS involves several different mechanisms with essentially the same outcome: the death of neurons, in this case motor neurons both in the cortex and lower (bulbar and spina cord) motor regions, irreversibly worsening patients’ conditions up to their death. Over the last 20 years several potentially disease modifying drugs have been developed and evaluated for efficacy in patients with ALS, all without showing significant clinical benefit (Barp et al., 2020). At present there is no disease modifying or even symptomatic therapy for the disease. The only approved drugs for the disease are riluzole and edaravone, that have been shown to modestly prolong the life of patients but do not arrest disease progression (Barp et al., 2020). In common with most other neurodegenerative conditions, the pathophysiological processes that culminate in neuronal death are diverse and include mitochondrial dysfunction, oxidative stress, excitotoxicity, inflammation and apoptosis, involving both neurons and surrounding glial cells (Coupé and Gordon, 2013; Gois et al., 2020; Dhasmana et al., 2022).

Our enhanced understanding of the mechanisms underpinning neurodegenerative disease progression and the lessons learned from the failed drug trials help us formulate expectations for potential disease modifying drugs. Thus, we postulate that targeting multiple disease mechanisms will be the most likely successful strategy, due to the multiple pathways involved in the progression of ALS and other neurodegenerative diseases and the heterogeneous patient populations. Targeting etiological factors (removing the triggers of the neurodegenerative process) will only eliminate the causes of neuron death at best halting the disease process in perhaps subsets of patients. However, this type of disease modification will unlikely result in functional improvement and will, by definition, be of limited relevance to many affected individuals. At best we can expect a stabilization of function or more realistically a slowing of the functional decline.

Achieving functional improvement in heterogeneous neurodegenerative diseases such as ALS is made more difficult because by the time symptoms emerge and the disease is diagnosed there is already significant neuronal loss and functional compromise in the affected CNS regions [reviewed by Turner et al. (2012)]. Additionally, given the limited regenerative potential in the CNS, protection and synaptic remodeling of the remaining healthy neuronal population offers the most likely opportunity for therapeutic benefit. Consequently, the achievement of clinical functional improvement requires a relatively large number of functional neurons. Hence, the expectation of clinical improvement is most realistic in the earlier stages of the disease process where efforts should focus on enhancing the function of surviving neurons and their supporting glia. Herein lies the advantage of a pharmacological strategy that awakens natural tissue repair molecules that can nourish disease compromised, but surviving, neurons and glia to re-establish functional homeostasis and protect and rescue viable neurons.

### Practical consequences of the MoA of ILB^®^

Since the MoA of ILB^®^ indicates the mobilization of the patients’ own neuroprotective potential, it follows that for maximal beneficial effect ideally the drug should be administered as early in the disease process as possible, when there are remaining neuronal populations available for the protective effect to have a significant clinical impact. It also means that the patient benefit from treatment will likely depend on the speed of the degenerative process. Patients with a fulminant form of the disease may benefit less from the treatment than those who have a slower disease progression. Equally, younger people with greater regenerative potential would likely benefit more than older patients. Therefore, the patient functional response to the drug will likely be highly heterogenous. Additionally, the non-linear dose response of ILB^®^ resembles the hormetic dose response of the growth factors and functional systems affected (Calabrese and Baldwin, 2002; Calabrese and Mattson, 2017). This also means that classical dose-finding studies may not be particularly informative for ILB^®^. Furthermore, we predict the beneficial effect of the drug requires a pulsed dosing regimen, rather than maintained doses of ILB^®^, to prevent the long-term sequestration of the growth factors in the blood. The growth factor mediated effects of ILB^®^ on synaptic remodeling and regeneration also mean that any drug that would affect neuronal glutamate signaling will potentially modulate the therapeutic effects of ILB^®^. Careful modeling of potential interference from drugs like riluzole and memantine is of practical importance for the design of pivotal phase III clinical trials of ILB^®^ with the aim of achieving regulatory approval for this drug.

## Conclusion

The results described demonstrate a unique mechanism of action of ILB^®^ (summarized in Figure 13) that mobilizes and modulates naturally occurring tissue repair molecules to restore cellular homeostasis and function, indicating the potential of this drug as the first disease modifying treatment for people with complex neurodegenerative conditions like ALS. Further clinical studies are needed to confirm the therapeutic benefit of ILB^®^ in ALS and other neurodegenerative conditions. Carefully designed studies will be required to elucidate the patient characteristics that determine patient response. The potential drug-drug interactions identified will also need further investigation and animal studies are under way to elucidate some of these already. While there is still a lot of open questions, the potential for ILB^®^ as a platform drug to treat heterogenous neurodegenerative disease is clear.

## Data Availability

The original contributions presented in the study are included in the article/Supplementary Material, further inquiries can be directed to the corresponding author. Expression datasets are available in the GEO database (NCBI): GSE210027 and GSE210028.

## Supplementary material

Supplementary Figure S1 The Effect of ILB® on the glutamate signaling pathway. **(A)** The effect of sTBI on the glutamate signaling pathway. The downregulation of the glutamate transporters (green) leads to an increase in extracellular glutamate (orange), while the downregulation of neuronal glutamate receptors (green) lead to an inhibition (blue) of neuronal function and synaptic plasticity. **(B)** ILB® at 1mg/kg dose in rats reverses the effect of sTBI on glutamate signaling by increasing the expression of glutamate transporters (both glial and neuronal–red) and reducing the extracellular glutamate concentrations (blue). Additionally, ILB® increases the expression of neuronal glutamate receptors (red) restoring neuronal signaling and synaptic plasticity (orange). The downregulated molecules are green, the upregulated molecules are red. The inhibited functions/molecules are blue, while activated molecules/functions are orange.

Supplementary Figure S2 The effect of ILB® on the mitochondrial dysfunction pathway. **(A)** sTBI leads to a disproportionate activation of complexes IV and V and inhibition of complex I. **(B)** ILB® at 1 mg/kg dose in rats reverses the effect of sTBI on the disproportionate activation/inhibition of these complexes normalizing mitochondrial function. The downregulated molecules are green, the upregulated molecules are red. The inhibited functions/molecules are blue, while activated molecules/functions are orange.

Supplementary Figure S3 The Effect of ILB® on the inflammasome. **(A)** The effect of sTBI on the inflammasome. sTBI leads to a strong activation of the inflammasome. **(B)** ILB® at 1 mg/kg dose in rats reverses the effect of sTBI on the activation of NLRP3. The downregulated molecules are green, the upregulated molecules are red. The inhibited functions/molecules are blue, while activated molecules/functions are orange.

Supplementary Figure S4 Calibrator for the Glypican 3 binding assay.

Supplementary Figure S5 Lowest level of Glypican detected above background (accepted runs only). Bars represent the average reading from triplicate values (both the lowest calibrator and background). Error bars represent the standard deviation (SD) from the triplicates.

Supplementary Figure S6 Recovery rate in the Glypican-3 binding assay. **(A)** Within assay variation. Minimum (green bars), average (blue bars) and maximum (red bars) are represented for each experiment separately. The error bars represent the SD of the recovery of different calibrators from the same plate. **(B)** Between assay variation. Minimum (green bars), average (blue bars) and maximum (red bars) are represented for each calibrator value. The error bars represent the SD of the recovery of different experiments.

Supplementary Figure S7 Bias in the Glypican-3 binding assay. **(A)** Within assay variation. Minimum (green bars), average (blue bars) and maximum (red bars) are represented for each experiment separately. The error bars represent the SD of the bias of different calibrators from the same plate. **(B)** Between assay variation. Minimum (green bars), average (blue bars) and maximum (red bars) are represented for each calibrator value. The error bars represent the SD of the bias of different experiments.

Supplementary Figure S8 Random error (variability) in the Glypican-3 binding assay. **(A)** Within assay variation. Minimum (green bars), average (blue bars) and maximum (red bars) are represented for each experiment separately. The error bars represent the SD of the %CV of different calibrators from the same plate. **(B)** Between assay variation. Minimum (green bars), average (blue bars) and maximum (red bars) are represented for each calibrator value. The error bars represent the SD of the random error of different experiments.

Supplementary Figure S9 Total error (variability) in the Glypican-3 binding assay. **(A)** Within assay variation. Minimum (green bars), average (blue bars) and maximum (red bars) are represented for each experiment separately. The error bars represent the SD of the TE of different calibrators from the same plate. **(B)** Between assay variation. Minimum (green bars), average (blue bars) and maximum (red bars) are represented for each calibrator value. The error bars represent the SD of the total error of different experiments.

Supplementary Figure S10 Sigma Metric (SM) for the calibrators of the Glypican-3 binding assay. **(A)** Within assay variation. Minimum (green bars), average (blue bars) and maximum (red bars) are represented for each experiment separately. The error bars represent the SD of the SM of different calibrators from the same plate. **(B)** Between assay variation. Minimum (green bars) average (blue bars) and maximum (red bars) are represented for each calibrator value. The error bars represent the SD of the total error of different experiments.

Supplementary Figure S11 The linearity of the Glypican-3 binding assay. Average calculated calibrators (black; error bars represent SD), minimum calculated calibrators (green) and maximum calculated calibrators (red).

Supplementary Figure S12 Variability of output calculations from the binding assay (simulated data). **(A)** Schild plot with the summated data. The black dots of the Log(DR-1) value indicates the possible variation of the output data with ±20% of the original EC50 calculation. Resulting Schild equation (black) compared to the ideal competitive antagonist equation fitted to the same data (red). **(B)** Variability of pA2 calculations due the assay variability—simulated data. Simulation based on data from experiments with 0.02 ug/ml ILB® (blue), simulation based on data from experiments with 0.04 ug/ml ILB® (red), simulation based on data from experiments with 0.08 ug/ml ILB® (black). **(C)** Variability of ILB® dose required to induce a 50% response. Change only calculated if calculated value is outside the range of values calculated with original experiment. Simulation based on data from experiments with 0.02 ug/ml ILB® (blue), simulation based on data from experiments with 0.04 ug/ml ILB® (red), simulation based on data from experiments with 0.08 ug/ml ILB® (black). **(D)** Small variability of pA2 translates to large variability in calculated ILB® dose required for 50% response. Simulation based on data from experiments with 0.02 ug/ml ILB® (blue), simulation based on data from experiments with 0.04 ug/ml ILB® (red), simulation based on data from experiments with 0.08 μg/ml ILB® (black).
