# Supplementary figures and images for: The mechanism of action of a novel neuroprotective low molecular weight dextran sulphate: New platform therapy for neurodegenerative diseases like Amyotrophic Lateral Sclerosis
Source: Front Pharmacol. 2022 Aug 30;13:983853. doi: 10.3389/fphar.2022.983853 (PMC9468270; doi:10.3389/fphar.2022.983853)

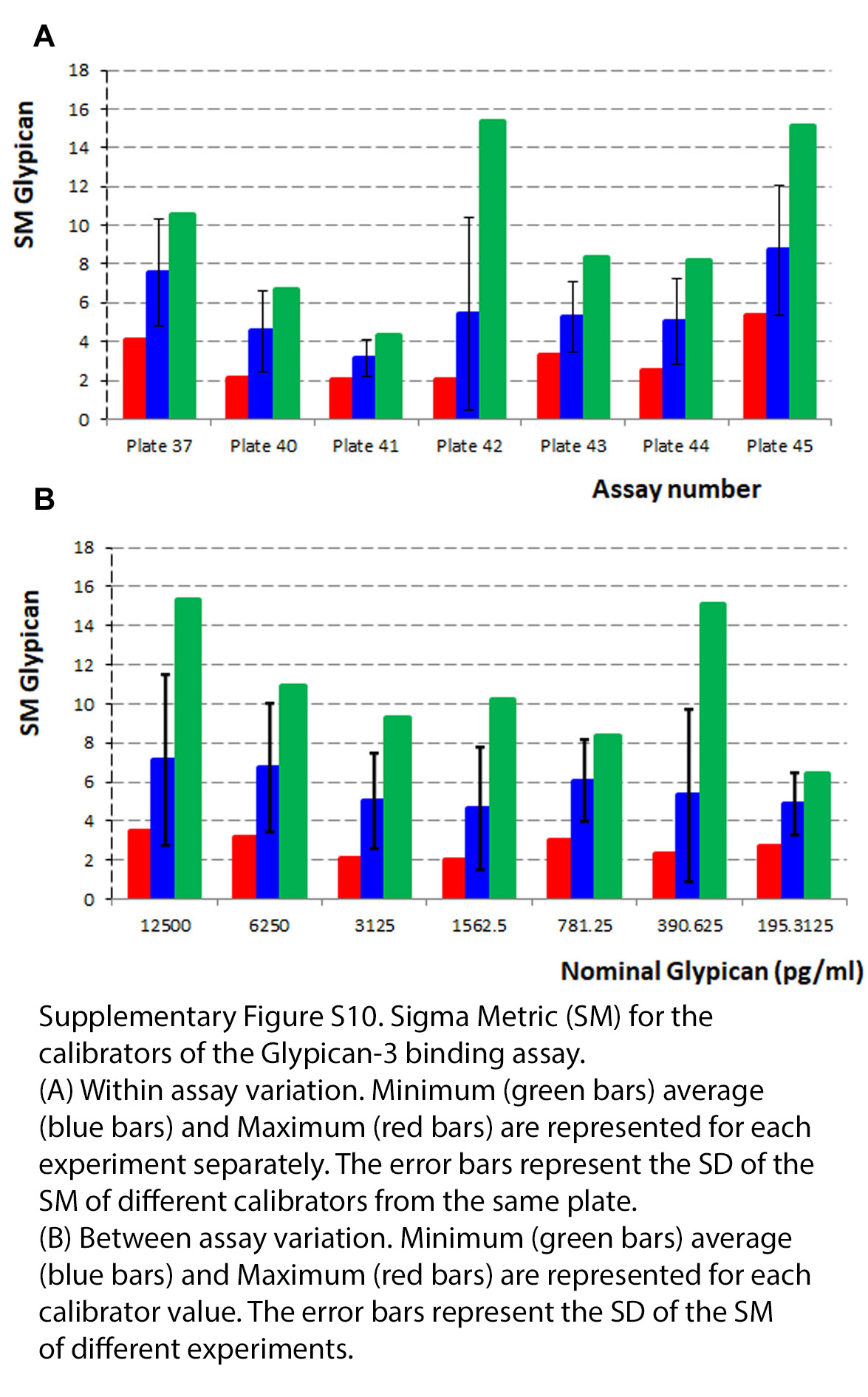

Supplement: Supplementary file 1 [file Image10.jpg]

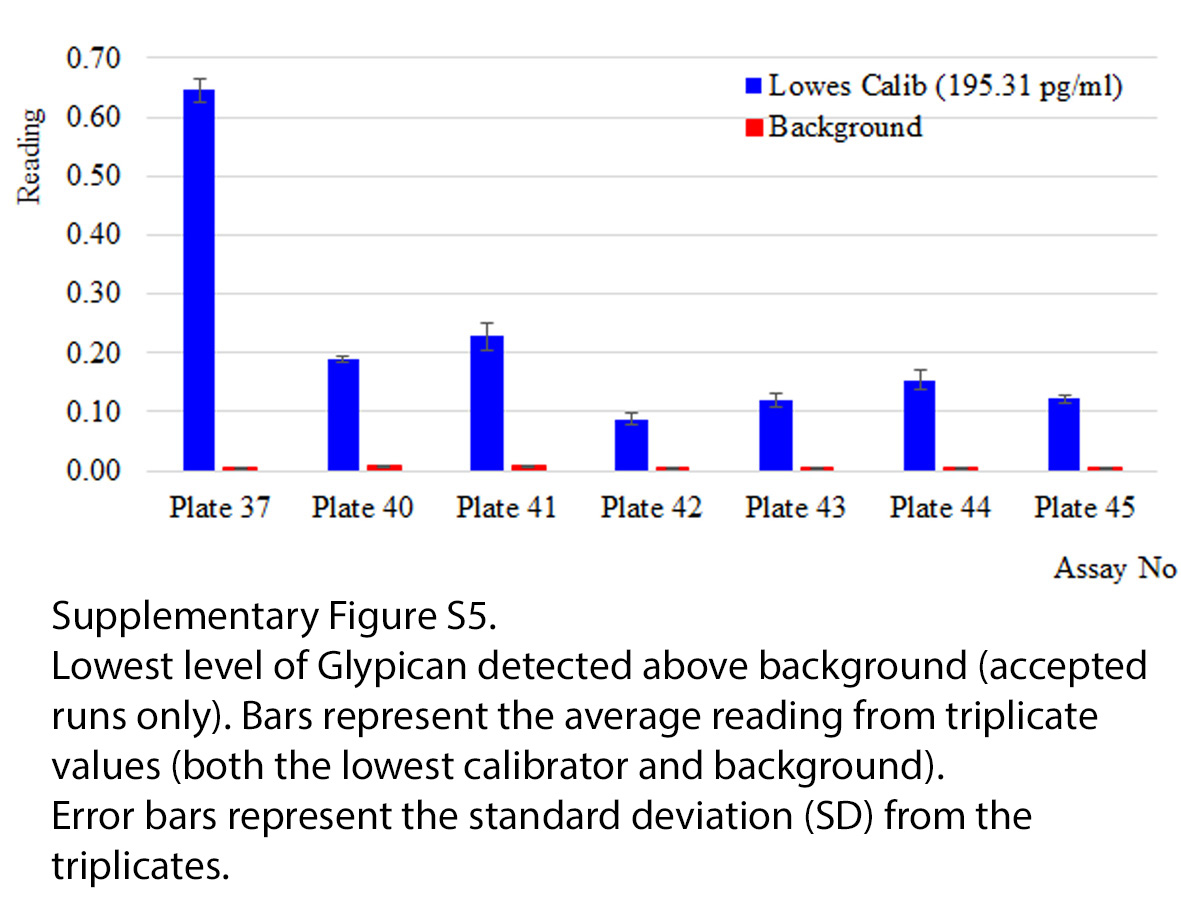

Supplement: Supplementary file 2 [file Image5.jpg]

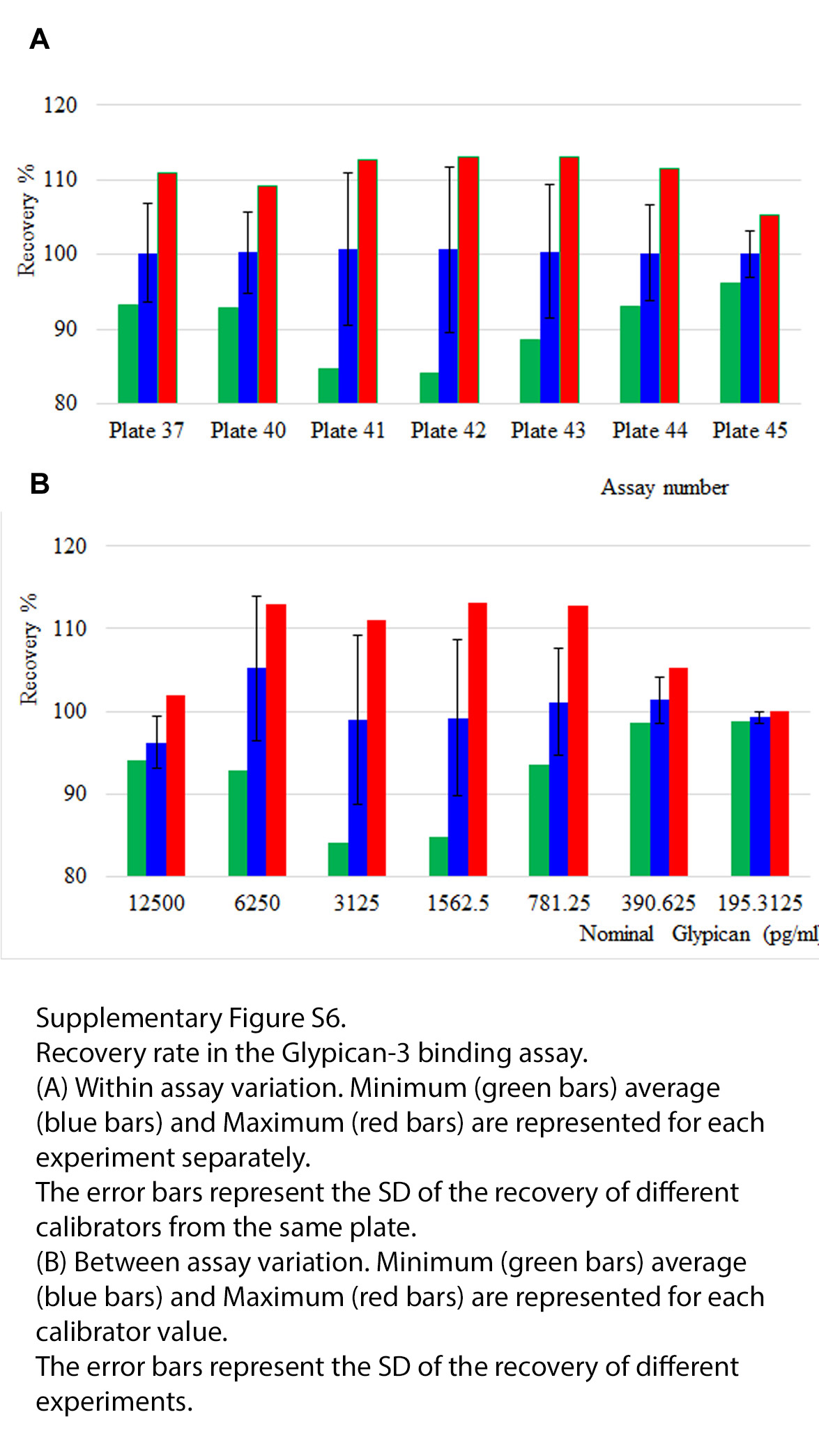

Supplement: Supplementary file 3 [file Image6.jpg]

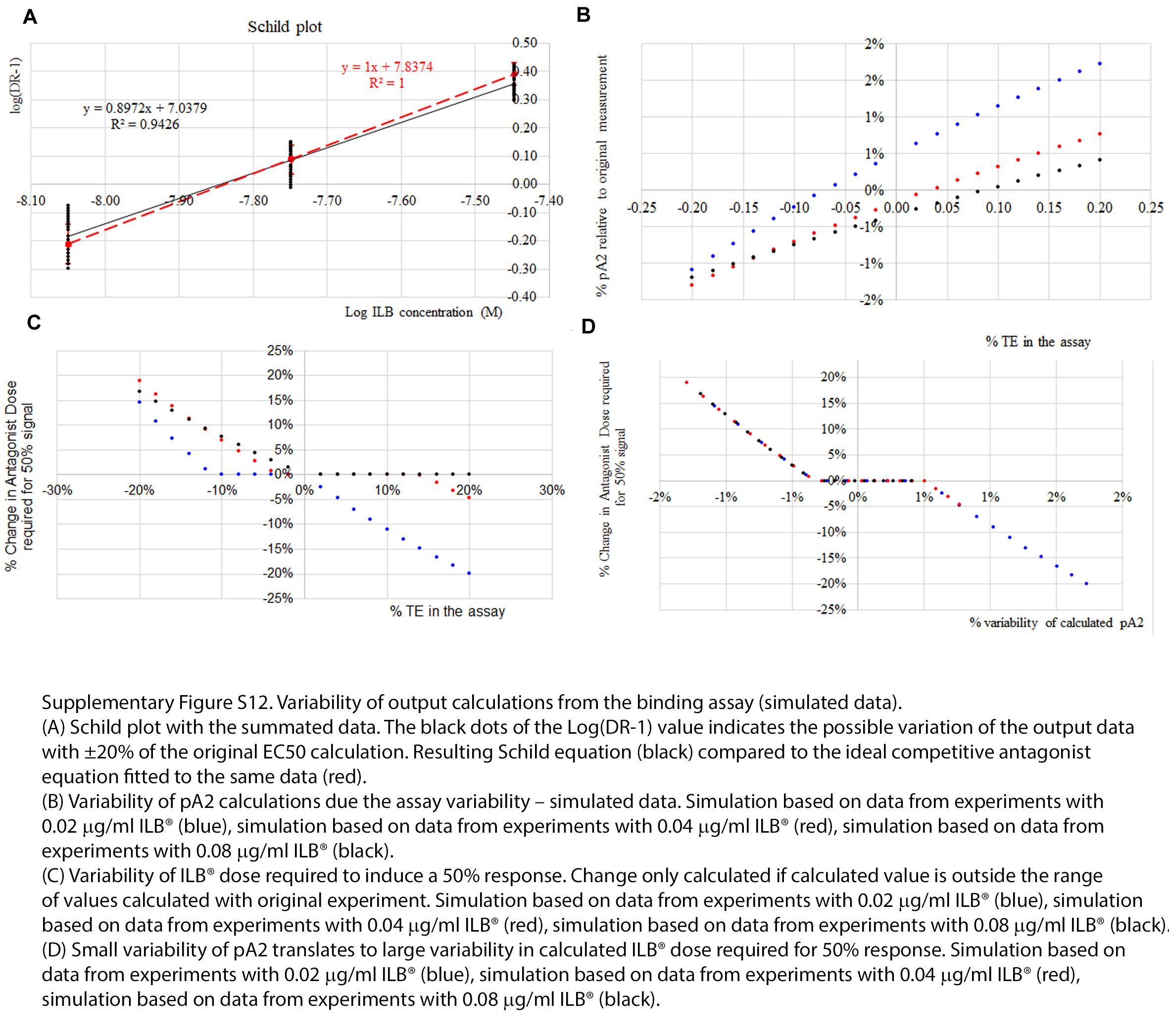

Supplement: Supplementary file 4 [file Image12.jpg]

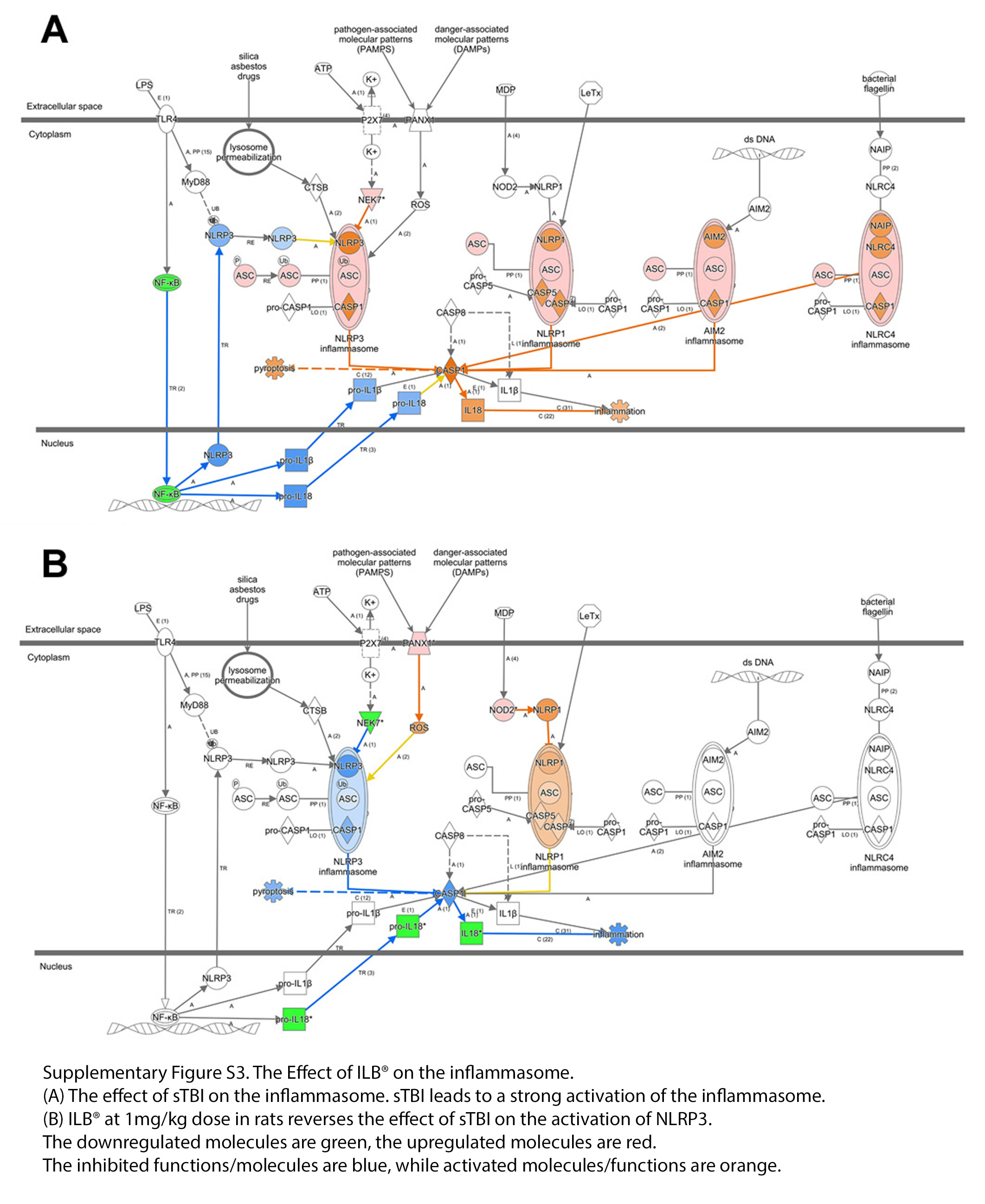

Supplement: Supplementary file 5 [file Image3.jpg]

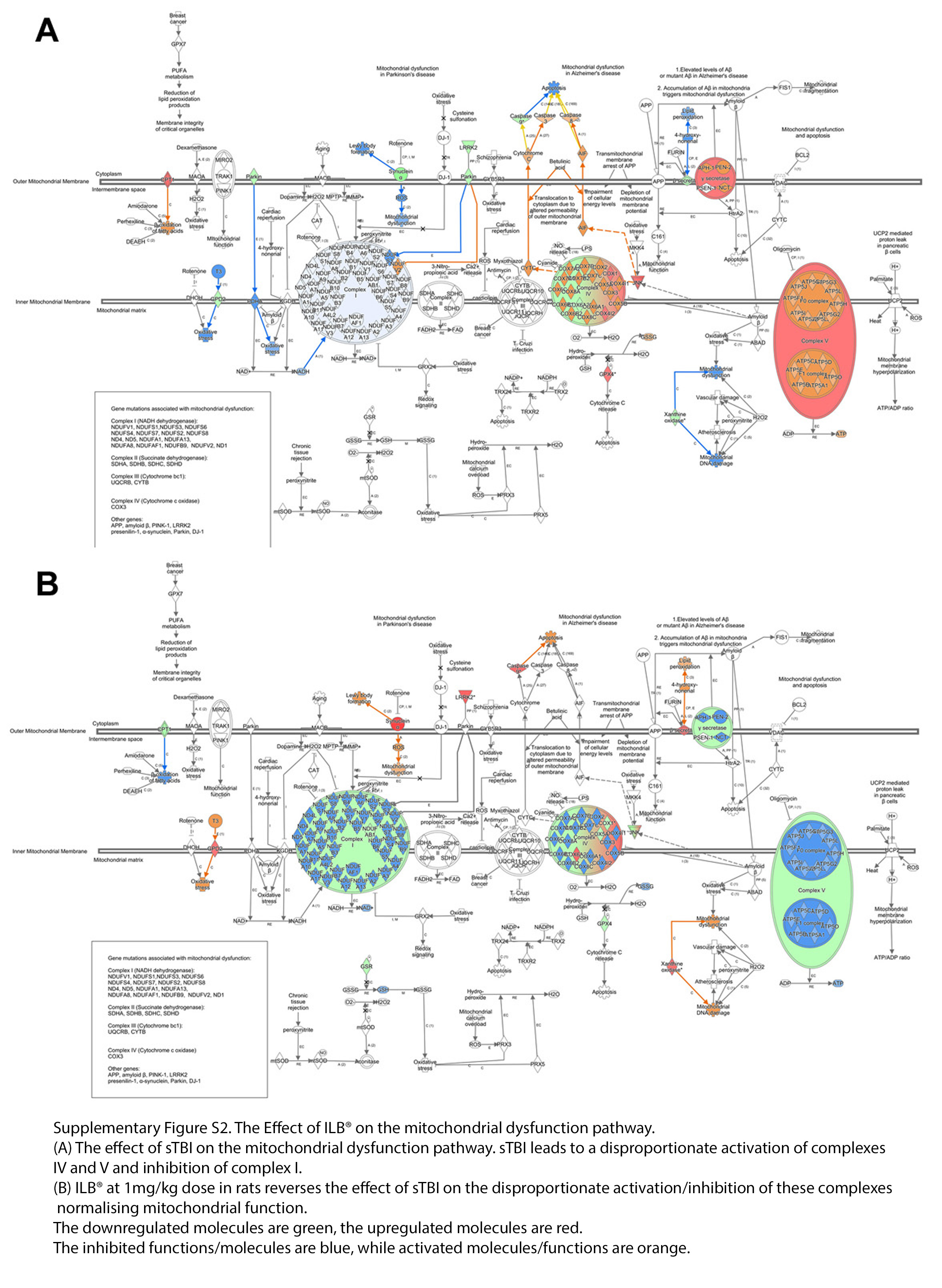

Supplement: Supplementary file 6 [file Image2.jpg]

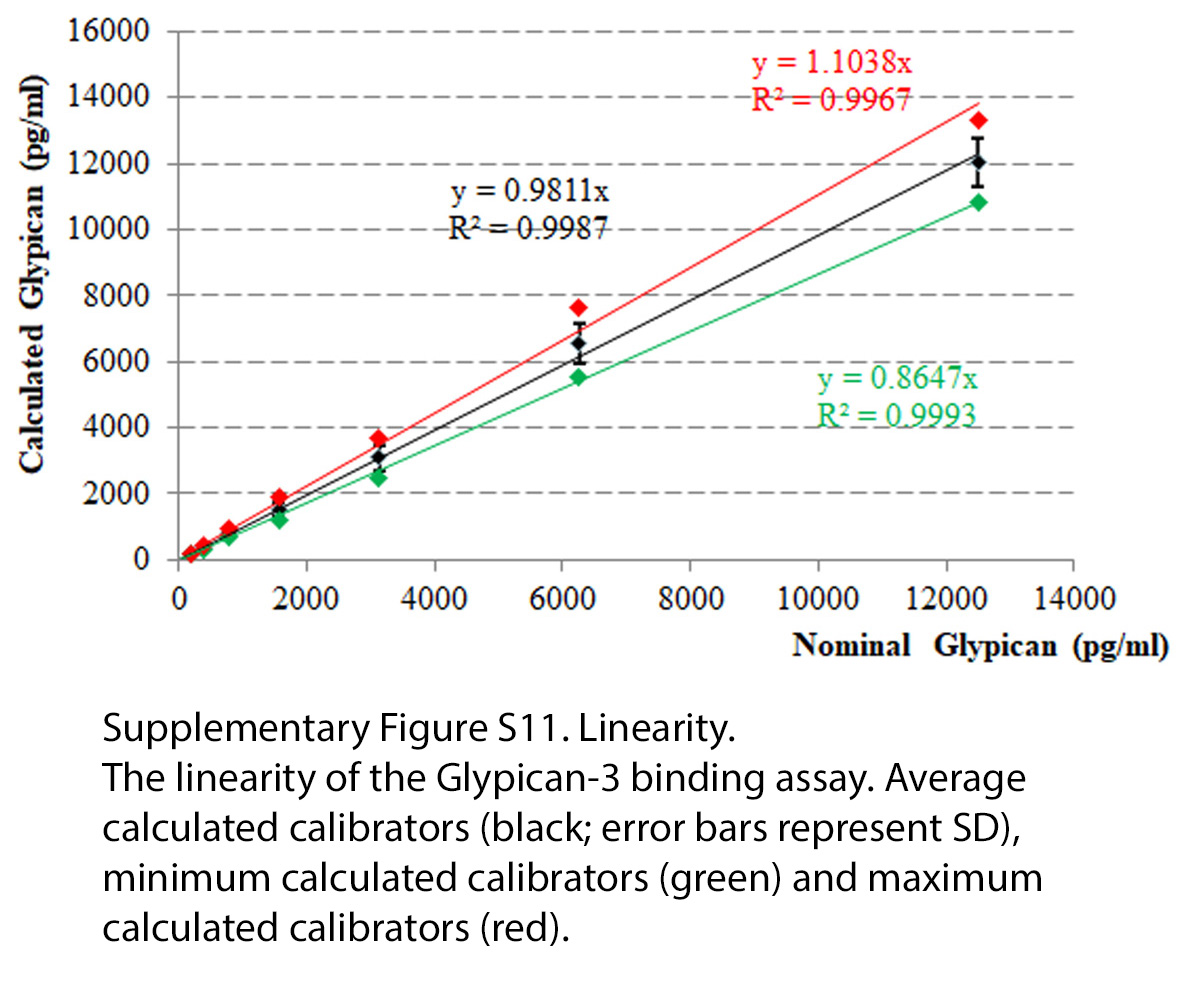

Supplement: Supplementary file 7 [file Image11.jpg]

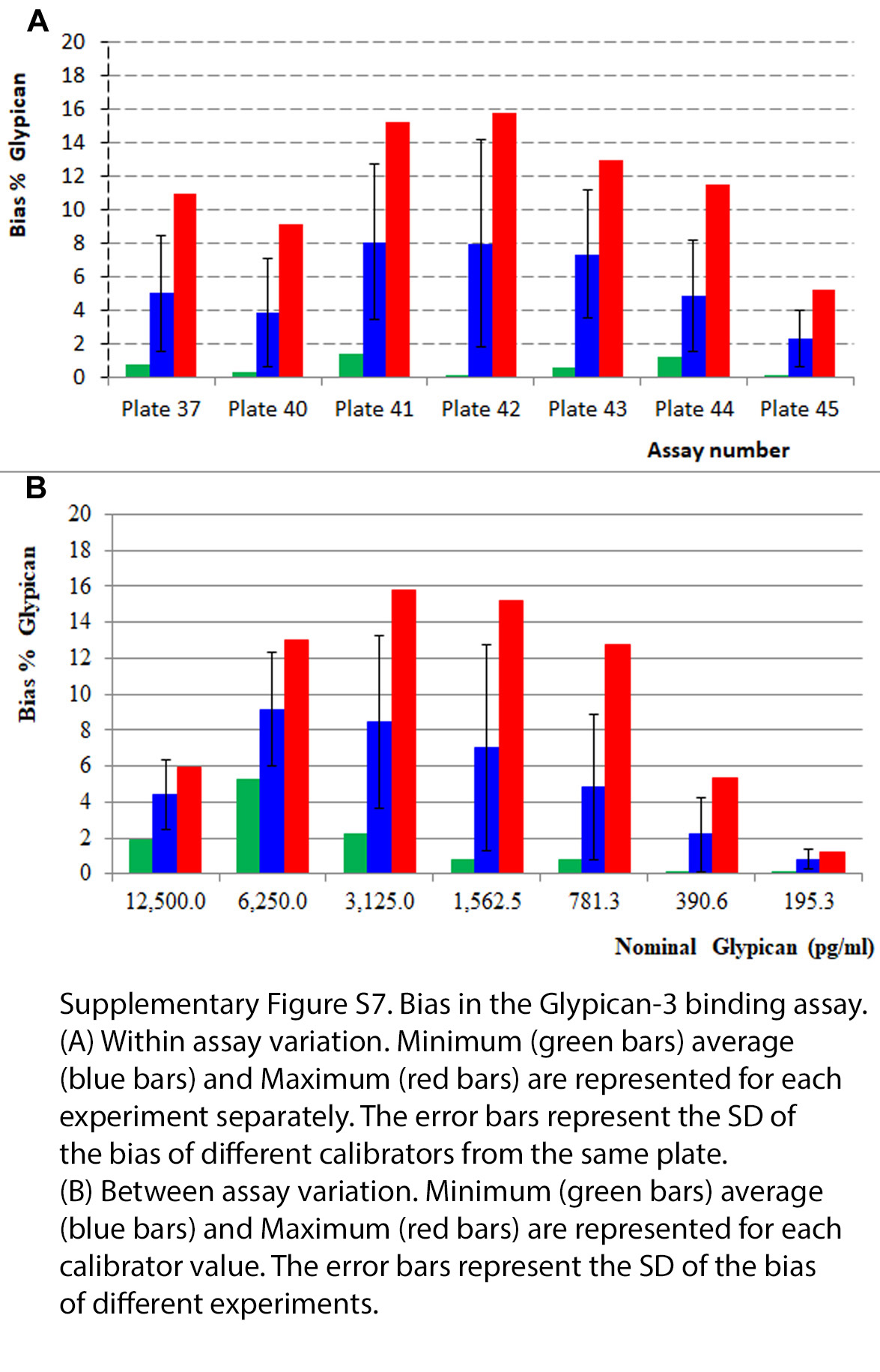

Supplement: Supplementary file 8 [file Image7.jpg]

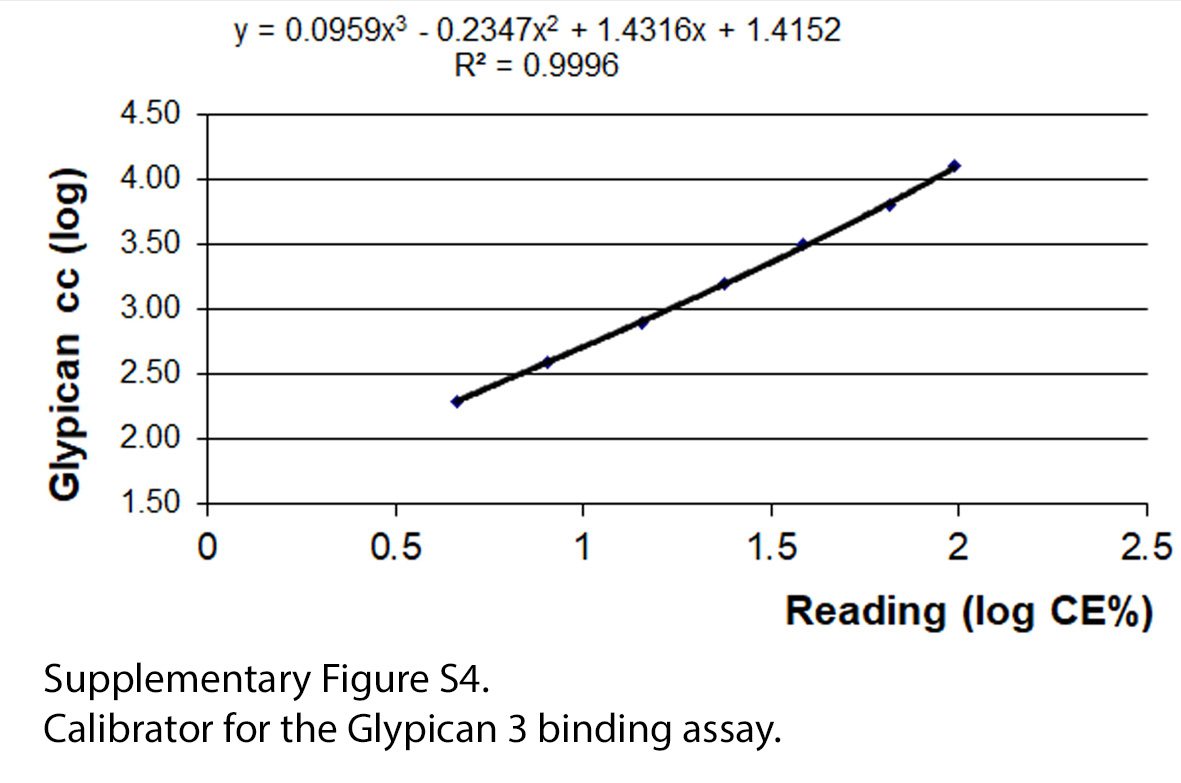

Supplement: Supplementary file 11 [file Image4.jpg]

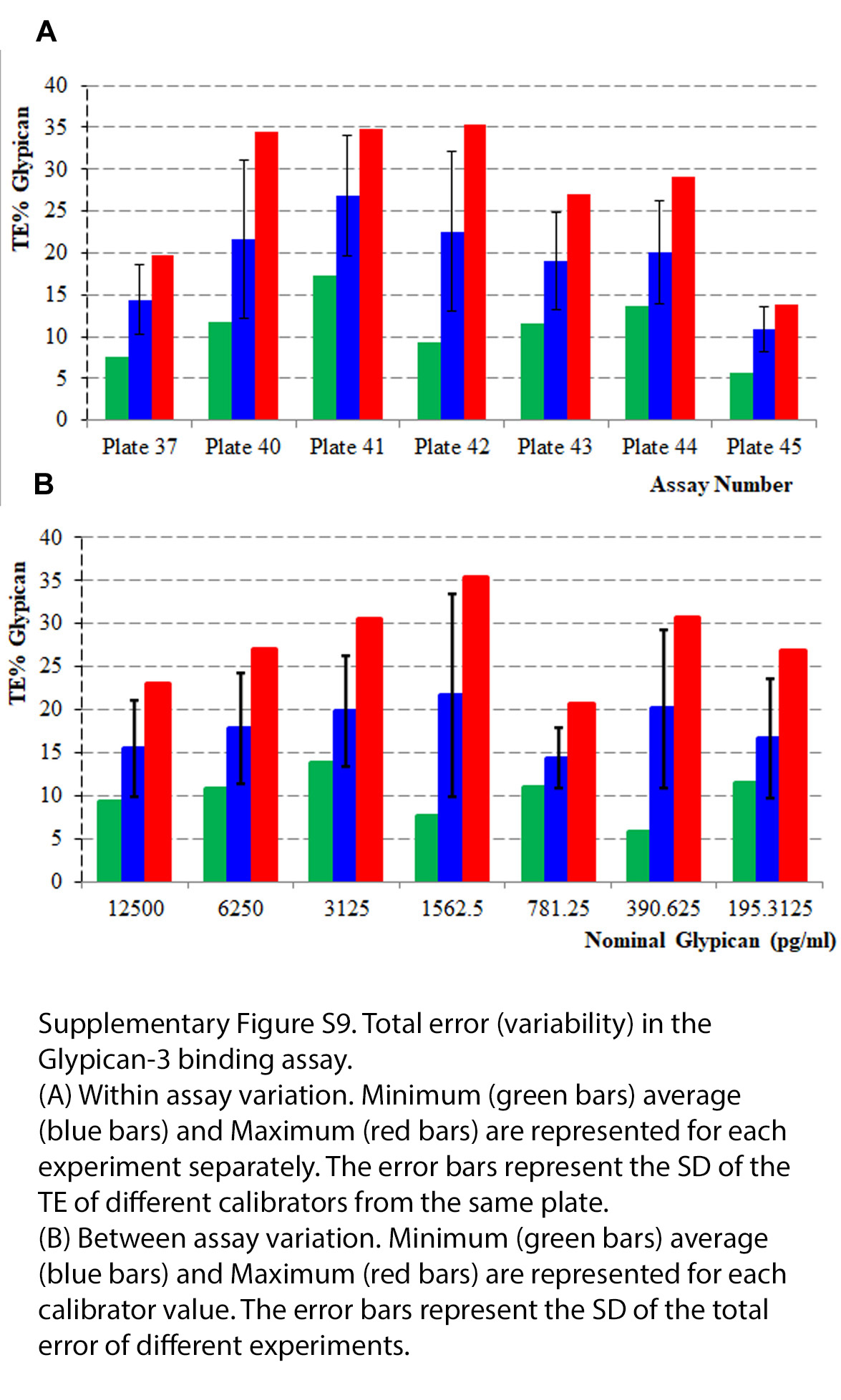

Supplement: Supplementary file 12 [file Image9.jpg]

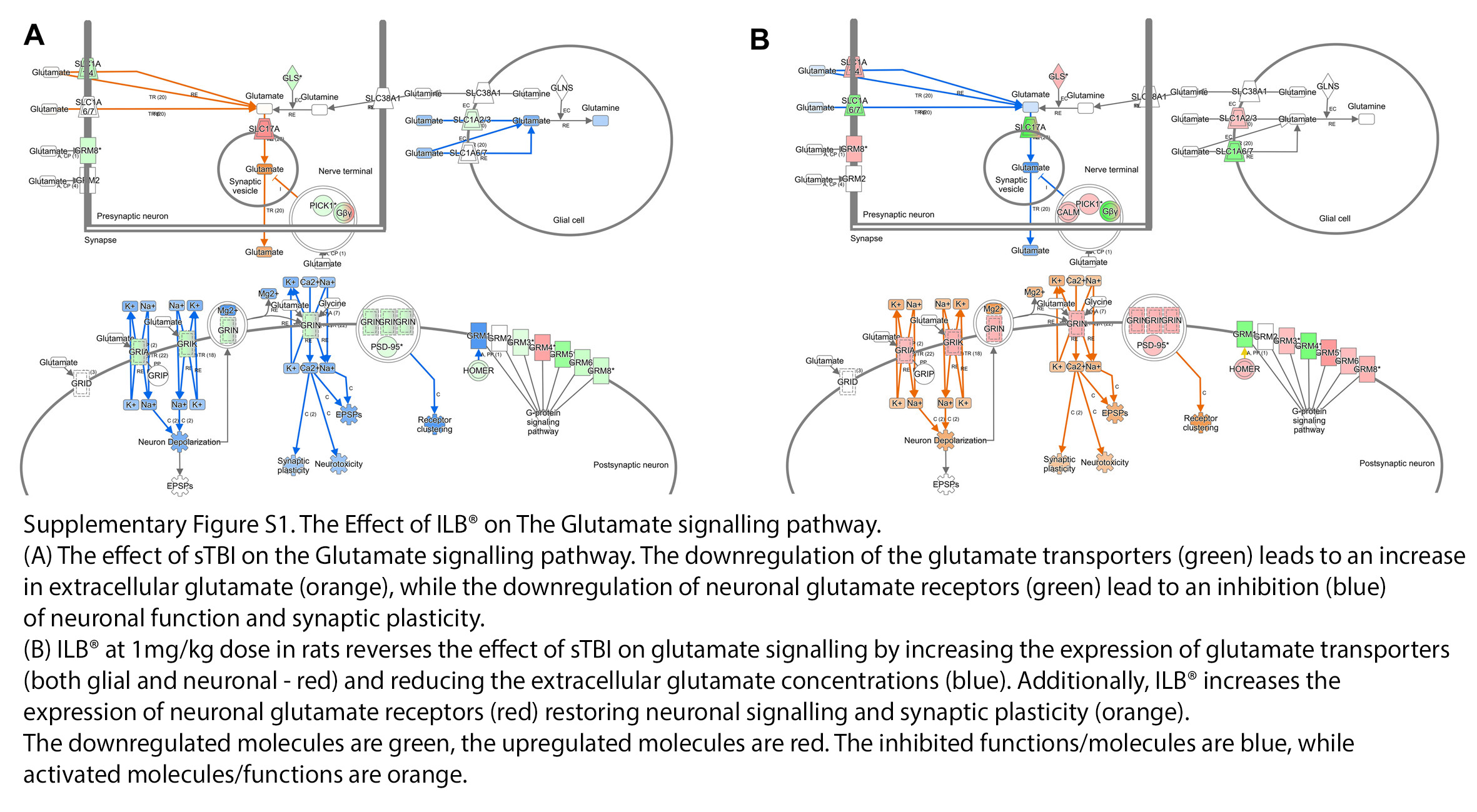

Supplement: Supplementary file 13 [file Image1.jpg]

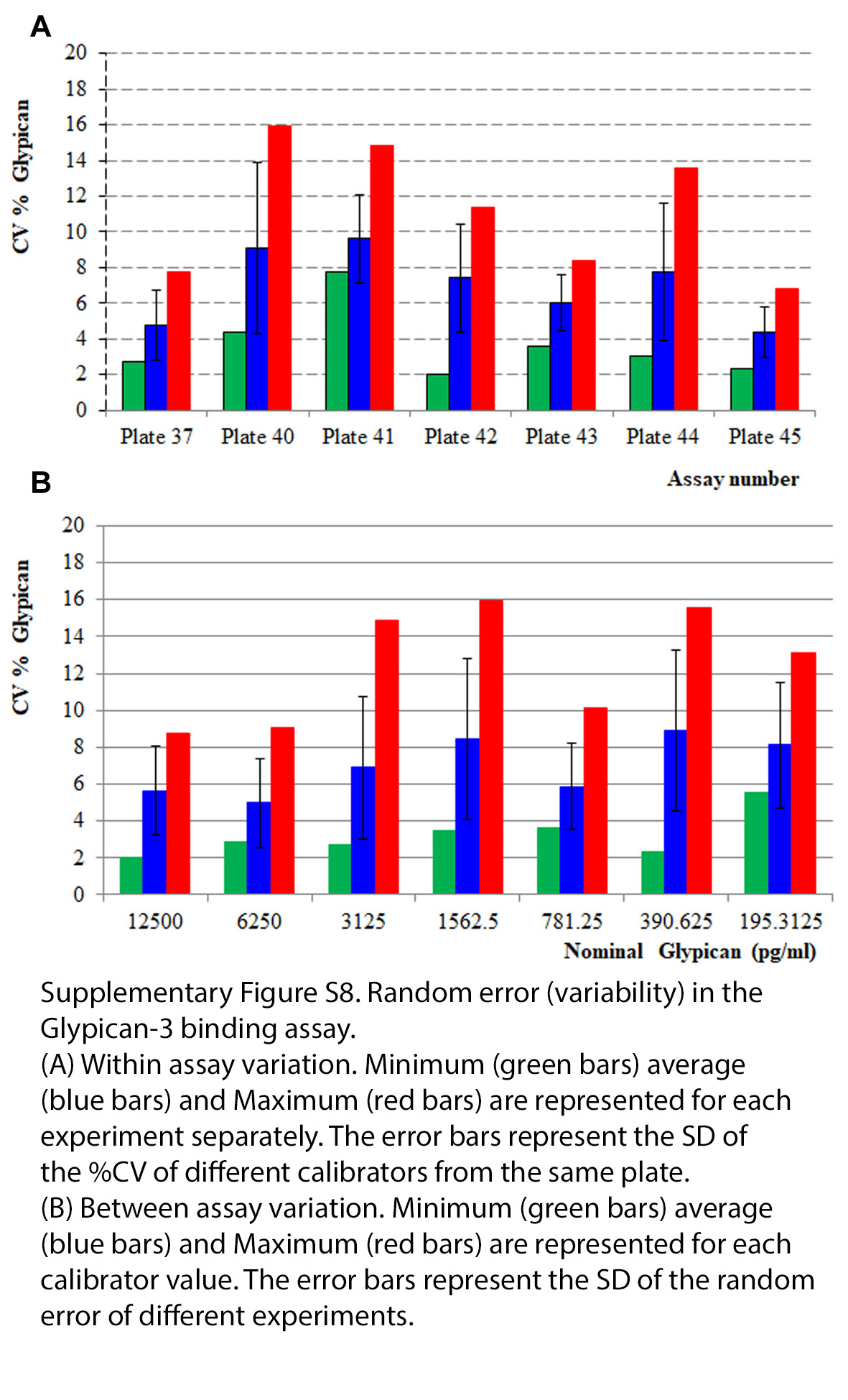

Supplement: Supplementary file 14 [file Image8.jpg]
