# Supplementary material for: The mechanism of action of a novel neuroprotective low molecular weight dextran sulphate: New platform therapy for neurodegenerative diseases like Amyotrophic Lateral Sclerosis
Source: Front Pharmacol. 2022 Aug 30;13:983853. doi: 10.3389/fphar.2022.983853 (PMC9468270; doi:10.3389/fphar.2022.983853)
Supplement: Supplementary file 9 [file DataSheet2.docx]

Supplementary Table 1. Canonical pathways affected by ILB^®^ in Schwann cell cultures.

Numerical values are the activation z scores (z>0 activation; z<0 inhibition) of the pathways based on the differential gene expression in the different data-sets.

| **Canonical Pathways** | **Schwann cells d0 to d2 Control** | **Schwann cells d2 C *vs* ILB^®^ 0.01 µg/ml** | **Schwann cells d2 C *vs* ILB^®^ 0.1 µg/ml** | **Schwann cells d2 C *vs* ILB^®^ 1.0 µg/ml** | **Schwann cells d2 C *vs* ILB^®^ 10 µg/ml** |
| --- | --- | --- | --- | --- | --- |
| Thrombin Signaling | -2.714 | 2.236 | 2.236 | 2.714 | 2.333 |
| NF-κB Signaling | -2.887 | 1.342 |  | 2.111 | 1.414 |
| Superpathway of Inositol Phosphate Compounds | -2.449 | 2 | 2 | 3.873 | 3 |
| Senescence Pathway | -2.111 | 2 | 2 | 2 | 2.333 |
| 3-phosphoinositide Biosynthesis | -2 | 2 |  | 3.606 | 2.646 |
| Huntington's Disease Signaling | 0.447 | 0.447 |  | 0.333 | 1 |
| Protein Kinase A Signaling | 0.535 | -1 | 0.378 | -0.943 | 0.333 |
| D-myo-inositol-5-phosphate Metabolism | -2 |  | 2 | 3.464 | 2.449 |
| Synaptogenesis Signaling Pathway | -4.025 |  |  | 3.357 | 2.828 |
| Reelin Signaling in Neurons | -3.606 |  |  | 2.646 | 2 |
| STAT3 Pathway | -3.606 |  |  | 2.646 | 3 |
| **FGF Signaling** | -2.828 |  |  | 2.828 | 2.646 |
| Pyridoxal 5'-phosphate Salvage Pathway | -2.646 |  |  | 2.828 | 2 |
| Salvage Pathways of Pyrimidine Ribonucleotides | -2.646 |  |  | 3 | 2.236 |
| Apelin Cardiomyocyte Signaling Pathway | -2.646 |  |  | 2.449 | 2.236 |
| Dopamine-DARPP32 Feedback in cAMP Signaling | -2.646 |  |  | 2.646 | 2.646 |
| cAMP-mediated signaling | -2.646 |  |  | 3.162 | 3 |
| B Cell Receptor Signaling | -2.449 |  |  | 1.604 | 1.414 |
| p70S6K Signaling | -2.449 |  |  | 2.646 | 2.121 |
| **PDGF Signaling** | -2.449 |  |  | 2.121 | 2.236 |
| **EGF Signaling** | -2.449 |  |  | 1.633 | 2.236 |
| **HGF Signaling** | -2.449 |  |  | 2.449 | 2.449 |
| IL-6 Signaling | -2.449 |  |  | 2.449 | 2.646 |
| Wnt/Ca+ pathway | -2.236 |  |  | 2.646 | 2 |
| GDNF Family Ligand-Receptor Interactions | -2.236 |  |  | 2.449 | 2.646 |
| Acute Phase Response Signaling | -2.236 |  |  | 2.646 | 2.828 |
| Role of BRCA1 in DNA Damage Response | -2 |  |  | 1.633 | 1.342 |
| FLT3 Signaling in Hematopoietic Progenitor Cells | -2 |  |  | 1.89 | 1.633 |
| CNTF Signaling | -2 |  |  | 2 | 2 |
| RANK Signaling in Osteoclasts | -2 |  |  | 2.449 | 2.236 |
| UVA-Induced MAPK Signaling | -2 |  |  | 2.236 | 2.236 |
| IL-17A Signaling in Airway Cells | -2 |  |  | 1.633 | 2.236 |
| Sphingosine-1-phosphate Signaling | -1.414 |  |  | 1 | 1.89 |
| Apelin Endothelial Signaling Pathway | -1.342 |  |  | 1.89 | 1.633 |
| NGF Signaling | -1.342 |  |  | 2.333 | 1.89 |
| Systemic Lupus Erythematosus In B Cell Signaling Pathway | -1.134 |  |  | 2.138 | 2.111 |
| Neurotrophin/TRK Signaling | -1 |  |  | 1.633 | 1 |
| AMPK Signaling | -1 |  |  | 2.121 | 1.134 |
| JAK/Stat Signaling | -0.447 |  |  | 2.449 | 2.236 |
| PPARα/RXRα Activation | 0.905 |  |  | -0.816 | -1 |
| PTEN Signaling | 2.887 |  |  | -1 | -1.342 |
| Renin-Angiotensin Signaling | -2 |  |  | 2.449 |  |
| Cell Cycle Control of Chromosomal Replication | -2 |  |  | 2.449 |  |
| Mitotic Roles of Polo-Like Kinase | -1.342 |  |  | 1.342 |  |
| Regulation of Cellular Mechanics by Calpain Protease | -1 |  |  |  |  |
| 3-phosphoinositide Degradation |  | 2 |  | 3.742 | 2.646 |
| D-myo-inositol (3,4,5,6)-tetrakisphosphate Biosynthesis |  |  |  | 3.317 | 2.236 |
| D-myo-inositol (1,4,5,6)-Tetrakisphosphate Biosynthesis |  |  |  | 3.317 | 2.236 |
| IL-9 Signaling |  |  |  | 2.449 | 2.236 |
| **Growth Hormone Signaling** |  |  |  | 2 | 2.236 |
| PCP pathway |  |  |  | 2.236 | 2 |
| PI3K Signaling in B Lymphocytes |  |  |  | 1.897 | 1.89 |
| Interferon Signaling |  |  |  | 1.342 | 2 |
| IL-3 Signaling |  |  |  | 1.342 | 1.342 |
| CD40 Signaling |  |  |  | 1.633 | 1 |
| LPS-stimulated MAPK Signaling |  |  |  | 2.449 |  |
| NER Pathway |  |  | -1 | 1 |  |
| Cell Cycle: G2/M DNA Damage Checkpoint Regulation |  |  |  | -2 |  |
| Estrogen-mediated S-phase Entry |  |  |  | 1 |  |
| Cell Cycle: G1/S Checkpoint Regulation |  |  |  | 0.447 |  |

Supplementary Table 2. Cellular and molecular functions affected by ILB^®^ in Schwann cell cultures.

Numerical values are the activation z scores (z>0 activation; z<0 inhibition) of the functions based on the differential gene expression in the different data-sets.

| **Molecular and Cellular Functions** | **Schwann cells d0 to d2 Control** | **Schwann cells d2 C *vs* ILB^®^ 0.01 µg/ml** | **Schwann cells d2 C *vs* ILB^®^ 0.1 µg/ml** | **Schwann cells d2 C *vs* ILB^®^ 1.0 µg/ml** | **Schwann cells d2 C *vs* ILB^®^ 10 µg/ml** |
| --- | --- | --- | --- | --- | --- |
| **Apoptosis** | 4.271 | -2.984 | -2.869 | -4.641 | -2.721 |
| **Mitosis** | -0.943 | -0.369 | 0.647 | 1.386 | 1.674 |
| **Differentiation of nervous system** | -2.686 | 0.986 |  | 2.409 | 2.363 |
| **Development of neurons** | -4.429 | 1.039 |  |  |  |
| **Microtubule dynamics** | -4.789 | 1.31 | 0.938 | 4.141 | 3.336 |
| Ploidy of cells | -0.176 | 1.538 | 2.207 | 0.221 |  |
| **Necrosis** | 2.134 |  | -2.776 | -4.012 | -1.577 |
| Proliferation of connective tissue cells | -2.319 |  |  | 1.781 |  |
| Organization of cytoplasm | -4.974 |  |  | 4.001 | 3.283 |
| Organization of cytoskeleton | -4.91 |  |  | 4.001 | 3.283 |
| Invasion of cells | -5.799 |  |  | 4.671 | 1.934 |
| Cell proliferation of tumor cell lines | -6.055 |  |  | 5.334 |  |
| **Cell movement** | -7.301 |  |  | 5.578 | 3.335 |
| **Migration of cells** | -7.219 |  |  | 5.94 | 3.893 |
| Cell viability of tumor cell lines | -7.307 |  |  | 6.701 |  |
| **Cell viability** | -8.674 |  |  | 7.44 | 5.226 |
| **Cell survival** | -8.75 |  |  | 7.885 | 5.649 |
| **Neuritogenesis** | -3.655 |  |  |  | 2.159 |
| Phosphorylation of protein | -2.132 |  |  |  | 2.251 |
| Endothelial cell development | -1.303 |  |  |  | 2.642 |
| Binding of tumor cell lines | -1.919 |  |  |  | 2.67 |
| Formation of cellular protrusions | -5.205 |  |  |  | 3.161 |
| Growth of neurites | -4.246 |  |  |  |  |
| Outgrowth of cells | -4.132 |  |  |  |  |
| Proliferation of neuronal cells | -3.884 |  |  |  |  |
| Outgrowth of neurites | -3.881 |  |  |  |  |
| Morphogenesis of neurons | -3.655 |  |  |  |  |
| Outgrowth of neurons | -3.611 |  |  |  |  |
| Cell spreading | -3.458 |  |  |  |  |
| Cell movement of fibroblast cell lines | -3.153 |  |  |  |  |
| Cell movement of endothelial cells | -2.765 |  |  |  |  |
| Migration of endothelial cells | -2.258 |  |  |  |  |
| Adhesion of connective tissue cells | -2.066 |  |  |  |  |
| Movement of vascular endothelial cells | -1.643 |  |  |  |  |
| Binding of connective tissue cells | -1.6 |  |  |  |  |
| Migration of vascular endothelial cells | -0.827 |  |  |  |  |
| Cell death of connective tissue cells | -0.051 |  |  |  |  |
| M phase |  | -0.365 | 1.102 | 1.539 | 1.857 |
| Cell viability of fibroblast cell lines |  | 0.555 |  |  |  |
| Differentiation of neurons |  | 0.714 |  | 2.756 | 2.678 |
| DNA replication |  | 0.883 | 0.883 |  |  |
| Cell cycle progression |  | 1.049 | 1.912 | 2.017 |  |
| Cell cycle progression of tumor cell lines |  | 1.154 |  |  | 0.9 |
| Ploidy |  | 1.189 | 1.746 | -0.249 |  |
| Metabolism of DNA |  | 1.278 | 1.063 | 1.176 |  |
| Interphase |  | 1.413 | 0.634 |  |  |
| Cytokinesis of tumor cell lines |  |  | 1 | 1.488 | 2.186 |
| M phase of cervical cancer cell lines |  |  | 1 | 0.816 |  |
| Repair of DNA |  |  | 1.961 |  |  |
| Formation of nuclear foci |  |  |  | -1.195 |  |
| G2 phase |  |  |  | 0.128 |  |
| Formation of cytoskeleton |  |  |  | 0.291 |  |
| Development of cytoplasm |  |  |  | 0.345 |  |
| Transcription of RNA |  |  |  | 0.498 |  |
| G2/M phase |  |  |  | 0.555 |  |
| Proliferation of embryonic cell lines |  |  |  | 0.56 |  |
| Formation of filaments |  |  |  | 0.561 |  |
| Fibrogenesis |  |  |  | 1.077 |  |
| Checkpoint control |  |  |  | 2.373 |  |
| Transactivation |  |  |  | 2.88 |  |
| Cell death of blood cells |  |  |  |  | 0.373 |
| Apoptosis of hematopoietic cell lines |  |  |  |  | 0.967 |
| Colony formation of cells |  |  |  |  | 1.052 |
| Cell death of hematopoietic cell lines |  |  |  |  | 1.211 |
| Apoptosis of leukocyte cell lines |  |  |  |  | 1.311 |
| Colony formation |  |  |  |  | 1.328 |
| Apoptosis of B-lymphocyte derived cell lines |  |  |  |  | 1.442 |
| Cell death of leukocyte cell lines |  |  |  |  | 1.55 |

Supplementary Table 3. Canonical pathways affected by ILB^®^ in SHSY5Y neuroblastoma cultures.

Numerical values are the activation z scores (z>0 activation; z<0 inhibition) of the pathways based on the differential gene expression in the different data-sets.

| **Canonical Pathways** | **SHSY5Y d0 to d2 Control** | **SHSY5Y d2 C *vs* ILB**^®^ **0.01 µg/ml** | **SHSY5Y d2 C *vs* ILB**^®^ **0.1 µg/ml** | **SHSY5Y d2 C *vs* ILB**^®^ **1.0 µg/ml** | **SHSY5Y d2 C *vs* ILB**^®^ **10 µg/ml** |
| --- | --- | --- | --- | --- | --- |
| IL-7 Signaling Pathway | -2 | 1 | 1 | 2 | 2 |
| Ephrin Receptor Signaling | 0 | 0 | 0 | 1 | 1 |
| mTOR Signaling | -0.447 | 0.447 | -0.447 | 0.447 | 1.342 |
| Insulin Receptor Signaling | -2.236 | 2.236 | 1.342 | 2.236 | 2.236 |
| IL-15 Signaling | -2 | 2 | 1 | 2 | 2 |
| Systemic Lupus Erythematosus In B Cell Signaling Pathway | -1.633 | 1.633 | 0.816 | 1.633 | 1.633 |
| Leukocyte Extravasation Signaling | -1.342 | 1.342 | 1.342 | 1.342 | 1.342 |
| Integrin Signaling | -1.342 | 1.342 | 1.342 | 1.342 | 1.342 |
| Hepatic Fibrosis Signaling Pathway | -1.134 | 1.134 | 1.134 | 1.134 | 1.134 |
| IL-3 Signaling | -1 | 1 | 1 | 1 | 1 |
| Neuregulin Signaling | -1 | 1 | 1 | 1 | 1 |
| Estrogen Receptor Signaling | -1 | 1 | 0.333 | 1 | 1 |
| PI3K/AKT Signaling | -1 | 1 | 0 | 1 | 1 |
| UVB-Induced MAPK Signaling | -1 | 1 | 0 | 1 | 1 |
| ERK/MAPK Signaling | -0.816 | 0.816 | 0 | 0.816 | 0.816 |
| Cardiac Hypertrophy Signaling (Enhanced) | -0.707 | 0.707 | 0 | 0.707 | 0.707 |
| p70S6K Signaling | -0.447 | 0.447 | 0.447 | 0.447 | 0.447 |
| Fcγ Receptor-mediated Phagocytosis in Macrophages and Monocytes | 0 | 0 | 0 | 0 | 1 |
| IL-8 Signaling | 0 | 0 | 0 | 0 | 0.816 |
| Synaptogenesis Signaling Pathway | -0.632 | 0.632 | -0.632 | 0.632 | 0.632 |
| Androgen Signaling | 1 | -1 | -1 | 0 | 0 |
| Synaptic Long Term Depression | 1.342 | -1.342 | -1.342 | -0.447 | -0.447 |
| Inhibition of Matrix Metalloproteases | 1 | -1 | -1 | -1 | -1 |
| Sirtuin Signaling Pathway | 1 | -1 | -1 | -1 | -1 |
| Phospholipase C Signaling | 1 | -1 | -1 | -1 | -1 |
| fMLP Signaling in Neutrophils | 0.447 | -0.447 | -0.447 | -0.447 | -0.447 |
| GNRH Signaling | 0.447 | -0.447 | -0.447 | -0.447 | -0.447 |
| Thrombin Signaling | 0.447 | -0.447 | -0.447 | -0.447 | -0.447 |
| Huntington's Disease Signaling | 0.447 | -0.447 | -0.447 | -0.447 | -0.447 |
| CREB Signaling in Neurons | 0.447 | -0.447 | -0.447 | -0.447 | -0.447 |
| CCR3 Signaling in Eosinophils | 0.447 | -0.447 | -0.447 | -0.447 | -0.447 |

Supplementary Table 4. Cellular and molecular functions affected by ILB^®^ in SHSY5Y neuroblastoma cultures.

Numerical values are the activation z scores (z>0 activation; z<0 inhibition) of the functions based on the differential gene expression in the different data-sets.

| **Functions** | **SHSY5Y d0 to d2 Control** | **SHSY5Y d2 C *vs* ILB^®^ 0.01 µg/ml** | **SHSY5Y d2 C *vs* ILB^®^ 0.1 µg/ml** | **SHSY5Y d2 C *vs* ILB^®^ 1.0 µg/ml** | **SHSY5Y d2 C *vs* ILB^®^ 10 µg/ml** |
| --- | --- | --- | --- | --- | --- |
| Cell survival | -1.474 | 0.522 | 1.234 | 2.374 | 2.676 |
| Formation of actin filaments | -0.8 | 0.077 | 1.007 | 1.729 | 1.729 |
| Formation of filaments | -0.776 | 0.059 | 0.981 | 1.698 | 1.698 |
| Formation of focal adhesions | -0.677 | -0.097 | 0.677 | 1.45 | 1.45 |
| Transcription | -0.493 | 0.147 | 0.639 | 1.161 | 1.161 |
| Development of cytoplasm | -0.46 | -0.19 | 0.158 | 1.295 | 1.295 |
| Cell movement | -1.794 | 1.547 | 0.942 | 2.891 | 2.633 |
| Cell viability | -1.674 | 0.692 | 1.161 | 2.337 | 2.636 |
| Receptor-mediated endocytosis | -1 | 0.2 | 1.6 | 1.6 | 1.6 |
| Expression of RNA | -0.429 | 0.074 | 0.903 | 1.121 | 1.121 |
| Transcription of RNA | -0.693 | 0.342 | 0.642 | 0.972 | 0.972 |
| Phosphorylation of protein | -2.148 | 2.148 | 2.796 | 2.796 | 2.796 |
| Organization of cytoskeleton | -1.479 | 1.479 | -0.205 | 2.518 | 2.518 |
| Angiogenesis | -1.466 | 0.446 | 1.919 | 1.352 | 1.862 |
| Vasculogenesis | -1.58 | 0.194 | 2.147 | 1.438 | 1.438 |
| Formation of cellular protrusions | -0.941 | 0.941 | -0.486 | 2.048 | 2.048 |
| Microtubule dynamics | -0.748 | 0.748 | -0.626 | 1.823 | 1.823 |
| Development of neurons | -0.769 | 0.312 | -0.957 | 1.612 | 1.612 |
| Cellular homeostasis | -1.236 | 1.256 | -0.39 | 1.435 | 1.535 |
| Cognition | 0.566 | 0 | 0 | 1.414 | 1.414 |
| Learning | 0.566 | 0 | 0 | 1.414 | 1.414 |
| Outgrowth of cells | -0.961 | -0.241 | -1.153 | 0.36 | 0.36 |
| Growth of neurites | -0.597 | -0.579 | -1.08 | 0.401 | 0.401 |
| Sprouting | -0.328 | -1.129 | -0.202 | 0.379 | 0.379 |
| Insulin sensitivity | -0.529 | 1.352 | 0.529 | 1.147 | 1.147 |
| Migration of neurons | -0.088 | -1.223 | 0.214 | 0.467 | 0.467 |
| Shape change of neurites | 0.292 | -0.292 | -0.982 | 0.982 | 0.982 |
| Proliferation of neuronal cells | -0.577 | -0.158 | -1.539 | 0.376 | 0.376 |
| Growth of embryo | -0.136 | 0.724 | -0.608 | 0.6 | 0.6 |
| Cell-cell contact | -0.462 | 0.986 | -0.245 | 0.462 | 0.462 |
| Growth of organism | -0.084 | 0.633 | -0.597 | 0.517 | 0.517 |
| Apoptosis | 0.061 | -1.089 | 1.759 | -0.175 | -0.491 |
| Organismal death | 1.314 | -0.74 | -2.063 | -3.019 | -3.385 |

Supplementary Table S5. Upstream regulators in Schwann cell cultures.

Numerical values are the activation z scores (z>0 activation; z<0 inhibition) of the functions based on the differential gene expression in the different data-sets.

| **Upstream regulators (Growth factors and cytokines)** | **Schwann cells d0 to d2 Control** | **Schwann cells d2 C *vs* ILB^®^ 0.01 µg/ml** | **Schwann cells d2 C *vs* ILB^®^ 0.1 µg/ml** | **Schwann cells d2 C *vs* ILB^®^ 1.0 µg/ml** | **Schwann cells d2 C *vs* ILB^®^ 10 µg/ml** |
| --- | --- | --- | --- | --- | --- |
| AREG | -2.043 | 1.342 | 1.89 | 2.523 | 1.772 |
| TGFB2 | -2.611 |  | -0.391 |  | 1.097 |
| ANGPT2 | -2.386 |  | 1.954 | 1.864 | 2.133 |
| INHA | 1.125 |  |  | -1.408 | -0.881 |
| TGFB3 | -2.016 |  |  | 1.639 |  |
| IL15 | -2.378 |  |  | 2.909 |  |
| EDN1 | -3.672 |  |  | 3.233 | 3.079 |
| TGFB1 | -5.672 |  |  | 3.331 | 2.643 |
| HGF | -3.854 |  |  | 3.505 | 3.543 |
| OSM | -4.162 |  |  | 3.956 | 3.332 |
| CSF2 | -3.954 |  |  | 4.343 | 3.161 |
| TNF | -4.614 |  |  | 4.752 | 4.158 |
| PDGFB | -1.942 |  |  |  | 1.27 |
| WNT3A | -3.476 |  |  |  | 2.234 |
| AGT | -3.008 |  |  |  | 2.398 |
| VEGFA | -3.029 |  |  |  | 2.697 |
| IL6 | -2.819 |  |  |  | 3.159 |
| CCN2 | -3.118 |  |  |  |  |
| FGF2 | -3.075 |  |  |  |  |
| EGF | -2.798 |  |  |  |  |
| CSF1 | -2.718 |  |  |  |  |
| NRG1 | -2.637 |  |  |  |  |
| FGF10 | -2.425 |  |  |  |  |
| INHBB | -2.216 |  |  |  |  |
| JAG1 | -2.176 |  |  |  |  |
| NAMPT | -1.387 |  |  |  |  |
| GDF9 | -0.671 |  |  |  |  |
| FGF1 | 0.336 |  |  |  |  |
| CNTF |  |  |  |  | 1.446 |
| TNFSF10 |  |  |  |  | 1.71 |
| IFNA1/IFNA13 |  |  |  |  | 1.932 |
| IFNA4 |  |  |  |  | 2.164 |
| IFNA2 |  |  |  |  | 2.258 |
| PRL |  |  |  |  | 2.384 |
| IL21 |  |  |  |  | 2.747 |
| IFNL1 |  |  |  |  | 3.109 |
| TNFSF11 |  |  |  |  | 3.179 |
| IL1B |  |  |  |  | 3.454 |
| IL1RN |  |  |  | -1.373 | -1.358 |
| IL1A |  |  |  | 2.307 | 3.394 |

Supplementary Table S6. Upstream regulators in SHSY5Y neuroblastoma cultures.

Numerical values are the activation z scores (z>0 activation; z<0 inhibition) of the functions based on the differential gene expression in the different data-sets.

| **Upstream Regulators (growth factors and Cytokines** | **SHSY5Y d0 to d2 Control** | **SHSY5Y d2 C *vs* ILB^®^ 0.01 µg/ml** | **SHSY5Y d2 C *v*s ILB^®^ 0.1 µg/ml** | **SHSY5Y d2 C *vs* ILB^®^ 1.0 µg/ml** | **SHSY5Y d2 C *vs* ILB^®^ 10 µg/ml** |
| --- | --- | --- | --- | --- | --- |
| PRL | -0.606 | -0.202 | 1.212 | 1.818 | 1.818 |
| WNT3A | -1.98 | 1.386 | 1.386 | 2.772 | 2.772 |
| TNF | -1.695 | 0.626 | 0.718 | 1.695 | 2.087 |
| FGF2 | -1.702 | 0.837 | 0.837 | 1.702 | 1.702 |
| BDNF | -1.134 | 0.378 | 0.378 | 1.89 | 1.89 |
| TGFB3 | -2.414 | 0.928 | 2.414 | 2.414 | 2.414 |
| AGT | -2.22 | 1.434 | 1.729 | 1.434 | 2.122 |
| IL1B | -1.48 | 0.932 | -0.643 | 1.727 | 2.372 |
| INHBA | -1.478 | -0.369 | 2.17 | 1.478 | 1.478 |
| TGFB2 | -1.131 | 1.131 | 1.131 | 1.131 | 1.98 |
| ANGPT2 | -0.987 | 0.987 | 0.987 | 0.987 | 1.571 |
| EDN1 | -1.725 | 1.725 | 1.725 | 1.725 | 1.725 |
| CCN2 | -1.183 | 1.183 | 1.183 | 1.183 | 1.183 |
| TGFB1 | -1.617 | -1.036 | 1.617 | 0.385 | 0.842 |
| IGF1 | -1.188 | 0.62 | 0.052 | 0.62 | 0.62 |
| IFNG | 0.151 | -1.323 | -0.984 | 0.129 | 0.21 |
| MIF | -0.051 | 0.051 | -0.977 | 0.051 | 0.051 |
| FGF1 | 2.177 | -1.408 | -2.177 | -1.408 | -0.128 |

Supplementary Table S7. Canonical pathways affected in the sTBI model compared with the canonical pathways affected by neurodegenerative diseases (based on the Ingenuity knowledge base – QIAGEN IPA, (QIAGEN Inc., https://digitalinsights.qiagen.com/IPA)

Numerical values represent -log(pvalue).

| **Ingenuity Canonical Pathways** | **ALS -log(p-value)** | **AD -log(p value)** | **Neurodegeneration-log(p value)** | **TBI day 7**  **- log(p value)** |
| --- | --- | --- | --- | --- |
| Axonal Guidance Signaling | 2.31 | 12.2 | 4.10 | 15.3 |
| CREB Signaling in Neurons | 3.93 | 7.49 | 7.72 | 9.71 |
| Opioid Signaling Pathway | 8.12 | 12.3 | 8.46 | 9.61 |
| Synaptic Long Term Depression | 4.25 | 4.51 | 6.86 | 9.24 |
| Synaptic Long Term Potentiation | 8.52 | 12.1 | 3.93 | 8.97 |
| GNRH Signaling |  | 8.56 | 4.72 | 8.6 |
| Molecular Mechanisms of Cancer | 4.72 | 16 | 7.80 | 8.53 |
| CXCR4 Signaling | 2.11 | 4.85 |  | 7.64 |
| Neuropathic Pain Signaling In Dorsal Horn Neurons | 8.99 | 15.3 | 5.01 | 7.41 |
| Cholecystokinin/Gastrin-mediated Signaling | 2.94 | 7.45 | 4.33 | 7.18 |
| Calcium Signaling | 17.2 | 18.9 | 6.35 | 7.1 |
| Osteoarthritis Pathway | 5.25 | 17.7 | 17.71 | 7.09 |
| Epithelial Adherens Junction Signaling | 4.48 | 2.74 | 1.86 | 6.62 |
| Endothelin-1 Signaling | 4.15 | 13.6 | 3.55 | 6.58 |
| Cardiac Hypertrophy Signaling | 10 | 12.7 | 6.67 | 6.35 |
| Glutamate Receptor Signaling | 16 | 7.97 | 10.49 | 6.22 |
| GPCR-Mediated Nutrient Sensing in Enteroendocrine Cells |  | 3.82 |  | 6.15 |
| Actin Cytoskeleton Signaling | 5.17 | 1.46 |  | 6.1 |
| UVC-Induced MAPK Signaling |  | 6.73 | 2.43 | 6.08 |
| Dopamine-DARPP32 Feedback in cAMP Signaling | 11.7 | 13.6 | 6.86 | 5.96 |
| Role of Macrophages, Fibroblasts and Endothelial Cells in Rheumatoid Arthritis | 2.53 | 16 | 15.29 | 5.79 |
| nNOS Signaling in Neurons | 11.7 | 12.6 | 4.08 | 5.03 |
| Hepatic Fibrosis / Hepatic Stellate Cell Activation | 10.2 | 15 | 23.77 | 4.83 |
| Huntington's Disease Signaling | 6.91 | 19.7 | 20.00 | 4.49 |
| G-Protein Coupled Receptor Signaling | 20.3 | 21.2 | 4.17 | 3.94 |
| cAMP-mediated signaling | 18.4 | 16 | 4.05 | 3.13 |
| Neuroinflammation Signaling Pathway | 31.7 | 49.6 | 37.49 | 2.65 |
| Gαs Signaling | 9.75 | 8.84 | 4.78 | 2.5 |
| GABA Receptor Signaling | 17.6 | 12.5 | 4.39 | 2.28 |
| eNOS Signaling | 9.05 | 30.3 | 4.27 | 2.12 |
| Amyotrophic Lateral Sclerosis Signaling | 28.3 | 21.1 | 29.41 | 1.83 |
| Acute Phase Response Signaling | 1.52 | 17.7 | 12.42 | 1.75 |
| AMPK Signaling | 19.7 | 27.8 | 4.69 |  |
| LXR/RXR Activation | 12.1 | 33.4 | 8.99 |  |
| Endocannabinoid Cancer Inhibition Pathway | 9.94 |  | 13.30 |  |
| Reelin Signaling in Neurons | 9.84 | 8.93 | 6.36 |  |
| Cardiac Hypertrophy Signaling (Enhanced) | 9.37 |  | 14.77 |  |
| Hepatic Fibrosis Signaling Pathway | 9.03 |  | 25.35 |  |
| Atherosclerosis Signaling | 7.97 | 19.1 | 5.70 |  |
| FXR/RXR Activation | 4.92 | 23.1 | 5.33 |  |
| Glucocorticoid Receptor Signaling | 4.37 | 15.3 | 10.64 |  |
| Clathrin-mediated Endocytosis Signaling | 3.61 | 13.7 | 4.28 |  |
| IL-12 Signaling and Production in Macrophages | 2.07 | 20.5 | 9.46 |  |
| Role of NFAT in Cardiac Hypertrophy | 2.62 | 9.66 | 7.52 | 5.84 |
| Phospholipase C Signaling | 2.61 | 4.07 | 1.69 | 5.81 |
| Role of Osteoblasts, Osteoclasts and Chondrocytes in Rheumatoid Arthritis | 5.81 | 9.32 | 8.07 | 5.73 |
| Agrin Interactions at Neuromuscular Junction | 2.42 | 3.92 | 2.24 | 5.72 |
| Aldosterone Signaling in Epithelial Cells |  | 4.37 | 2.62 | 5.68 |
| Protein Kinase A Signaling | 4.03 | 6.85 | 5.37 | 5.66 |
| PTEN Signaling | 5.19 | 9.46 | 8.99 | 5.48 |
| Gap Junction Signaling | 6.16 | 12 | 2.87 | 5.46 |
| G Beta Gamma Signaling |  | 5.74 | 3.42 | 5.43 |
| Wnt/β-catenin Signaling |  | 1.52 | 6.96 | 5.4 |
| Thrombin Signaling | 1.67 | 2.86 | 1.90 | 5.28 |
| Glioblastoma Multiforme Signaling | 4.86 | 4.54 | 4.97 | 5.27 |
| Corticotropin Releasing Hormone Signaling | 3.03 | 8.06 | 10.23 | 5.27 |
| Tec Kinase Signaling | 1.67 | 5.08 |  | 5.14 |
| Cellular Effects of Sildenafil (Viagra) | 8.24 | 4.53 | 1.55 | 5.01 |
| IL-8 Signaling | 2.29 | 11 | 5.98 | 4.97 |
| Ephrin Receptor Signaling | 4.56 | 5.28 | 3.99 | 4.95 |
| Colorectal Cancer Metastasis Signaling | 4.47 | 10.9 | 13.82 | 4.93 |
| PPARα/RXRα Activation | 1.87 | 7.85 | 3.72 | 4.89 |
| Neuregulin Signaling | 1.38 | 7.58 | 5.17 | 4.84 |
| Ephrin B Signaling |  |  |  | 4.83 |
| GP6 Signaling Pathway | 1.65 | 2.21 | 1.87 | 4.79 |
| UVA-Induced MAPK Signaling | 2.04 | 7.39 | 4.47 | 4.72 |
| Regulation of the Epithelial-Mesenchymal Transition Pathway |  | 3.83 | 8.57 | 4.72 |
| Signaling by Rho Family GTPases | 3.43 | 2.11 | 2.77 | 4.71 |
| Pyridoxal 5'-phosphate Salvage Pathway |  | 5.4 | 1.74 | 4.59 |
| ErbB Signaling |  | 7.23 | 4.66 | 4.46 |
| α-Adrenergic Signaling | 3.37 | 6.55 |  | 4.45 |
| Fcγ Receptor-mediated Phagocytosis in Macrophages and Monocytes | 1.39 | 8.37 |  | 4.45 |
| Renin-Angiotensin Signaling | 1.61 | 12.7 | 5.70 | 4.39 |
| Natural Killer Cell Signaling |  | 4.98 |  | 4.39 |
| RhoGDI Signaling | 1.97 | 1.36 |  | 4.34 |
| GPCR-Mediated Integration of Enteroendocrine Signaling Exemplified by an L Cell | 4.07 | 4.32 |  | 4.3 |
| *HGF Signaling* | 1.79 | 8.28 | 4.67 | 4.2 |
| Gαq Signaling | 8.38 | 11.2 |  | 4.19 |
| 14-3-3-mediated Signaling | 3.56 | 12.6 | 4.87 | 4.18 |
| P2Y Purigenic Receptor Signaling Pathway |  | 7.33 | 3.29 | 3.99 |
| PCP pathway |  |  | 1.47 | 3.93 |
| Thyroid Cancer Signaling |  | 10 | 8.20 | 3.91 |
| Melatonin Signaling | 1.74 | 9.33 |  | 3.9 |
| Mouse Embryonic Stem Cell Pluripotency |  | 1.6 | 4.32 | 3.87 |
| IL-3 Signaling |  | 4.57 | 2.26 | 3.86 |
| Nitric Oxide Signaling in the Cardiovascular System | 4.82 | 8.77 | 6.32 | 3.8 |
| Integrin Signaling | 3.31 | 1.71 |  | 3.8 |
| Androgen Signaling |  | 3.59 | 3.24 | 3.8 |
| Adrenomedullin signaling pathway | 4.11 | 10.9 | 7.34 | 3.79 |
| *NGF Signaling* | 3.02 | 8.44 | 6.79 | 3.79 |
| Fc Epsilon RI Signaling |  | 5.69 | 1.40 | 3.79 |
| Paxillin Signaling |  | 1.85 |  | 3.79 |
| Semaphorin Signaling in Neurons | 1.37 | 1.32 |  | 3.69 |
| FLT3 Signaling in Hematopoietic Progenitor Cells |  | 2.09 | 3.65 | 3.56 |
| fMLP Signaling in Neutrophils |  | 3.73 | 2.43 | 3.54 |
| Phagosome Formation |  | 6.34 |  | 3.42 |
| Ovarian Cancer Signaling | 2 | 7.22 | 5.19 | 3.41 |
| VDR/RXR Activation | 1.72 | 5.14 | 2.26 | 3.39 |
| Leukocyte Extravasation Signaling | 2.3 | 7.29 |  | 3.28 |
| Salvage Pathways of Pyrimidine Ribonucleotides |  | 3.45 |  | 3.27 |
| Wnt/Ca+ pathway |  | 4.5 | 3.46 | 3.24 |
| Role of NANOG in Mammalian Embryonic Stem Cell Pluripotency |  |  | 2.52 | 3.24 |
| Virus Entry via Endocytic Pathways |  | 3.7 |  | 3.22 |
| Type II Diabetes Mellitus Signaling | 1.71 | 8.66 | 7.38 | 3.2 |
| Rac Signaling |  | 2.55 | 2.61 | 3.17 |
| CCR3 Signaling in Eosinophils | 2.18 | 3.56 | 1.31 | 3.14 |
| HER-2 Signaling in Breast Cancer | 1.63 | 3.69 | 2.79 | 3.08 |
| Caveolar-mediated Endocytosis Signaling |  | 1.71 |  | 3.04 |
| CCR5 Signaling in Macrophages | 1.41 | 6.02 | 1.80 | 3.01 |
| Sperm Motility | 4.84 | 4.6 | 5.07 | 2.95 |
| Regulation of Actin-based Motility by Rho | 4.46 |  |  | 2.94 |
| Adipogenesis pathway | 3.43 | 4.9 | 8.63 | 2.91 |
| Growth Hormone Signaling | 1.79 | 7.52 | 2.37 | 2.88 |
| B Cell Receptor Signaling | 1.43 | 9.39 | 3.26 | 2.86 |
| PI3K Signaling in B Lymphocytes | 1.42 | 7.83 | 3.15 | 2.81 |
| Role of Tissue Factor in Cancer | 3.04 | 5.64 | 4.49 | 2.79 |
| Human Embryonic Stem Cell Pluripotency |  | 3.84 | 12.97 | 2.78 |
| Germ Cell-Sertoli Cell Junction Signaling | 3.35 | 3.74 | 2.51 | 2.65 |
| Glioma Signaling | 3.82 | 7.19 | 5.27 | 2.64 |
| Production of Nitric Oxide and Reactive Oxygen Species in Macrophages | 2.42 | 13.6 | 8.49 | 2.59 |
| Cardiac β-adrenergic Signaling | 3.14 | 2.38 |  | 2.57 |
| UVB-Induced MAPK Signaling | 1.53 | 7.78 | 3.20 | 2.55 |
| ILK Signaling | 7.49 | 6.88 | 5.66 | 2.22 |
| STAT3 Pathway | 3.4 | 10.5 | 16.33 | 2.2 |
| NRF2-mediated Oxidative Stress Response | 1.94 | 8.73 | 5.75 | 2.16 |
| RhoA Signaling | 5.28 |  |  | 2.14 |
| Serotonin Receptor Signaling | 3.22 | 7.25 |  | 2.1 |
| PEDF Signaling | 4.96 | 6.46 | 5.19 | 2.05 |
| Breast Cancer Regulation by Stathmin1 | 3.44 | 6.16 |  | 2 |
| Sertoli Cell-Sertoli Cell Junction Signaling | 3.03 | 6.01 | 3.20 | 1.95 |
| Apoptosis Signaling | 8.24 | 13 | 7.87 | 1.9 |
| Gαi Signaling | 6.97 | 9.53 | 1.33 | 1.9 |
| *PDGF Signaling* |  | 7.37 | 4.75 | 1.9 |
| Glutamate Dependent Acid Resistance | 3.01 |  |  | 1.88 |
| Sphingosine-1-phosphate Signaling | 4.55 | 5.16 | 5.16 | 1.82 |
| CDK5 Signaling | 2.48 | 8.42 | 5.51 | 1.8 |
| Pancreatic Adenocarcinoma Signaling | 4.01 | 9.83 | 14.08 | 1.74 |
| LPS-stimulated MAPK Signaling |  | 7.23 | 2.85 | 1.74 |
| Role of MAPK Signaling in the Pathogenesis of Influenza | 4 | 5.02 | 5.15 | 1.51 |
| IL-6 Signaling | 1.64 | 7.54 | 7.40 | 1.49 |
| Circadian Rhythm Signaling | 7.92 | 4.34 | 5.25 | 1.45 |
| Inhibition of Angiogenesis by TSP1 | 2.81 | 11.4 | 7.17 | 1.45 |
| Dendritic Cell Maturation | 2.56 | 10.3 | 8.11 | 1.43 |
| NF-κB Signaling | 2.06 | 8.69 | 15.33 | 1.42 |
| nNOS Signaling in Skeletal Muscle Cells | 3.51 | 2.14 | 5.44 | 1.39 |
| HMGB1 Signaling | 2.24 | 7.95 | 8.90 | 1.33 |
| Induction of Apoptosis by HIV1 | 8.26 | 10.2 | 6.40 |  |
| Synaptogenesis Signaling Pathway | 8.12 |  | 8.59 |  |
| Myc Mediated Apoptosis Signaling | 7.98 | 8.62 | 3.46 |  |
| Endocannabinoid Neuronal Synapse Pathway | 7.38 |  | 7.46 |  |
| Docosahexaenoic Acid (DHA) Signaling | 7.08 | 5.05 | 6.68 |  |
| Tumoricidal Function of Hepatic Natural Killer Cells | 6.32 | 5.78 | 3.98 |  |
| Senescence Pathway | 6.09 |  | 9.84 |  |
| VEGF Signaling | 5.97 | 8.76 | 5.01 |  |
| Type I Diabetes Mellitus Signaling | 5.71 | 6.72 | 6.32 |  |
| Death Receptor Signaling | 5.57 | 6.46 | 3.27 |  |
| Sirtuin Signaling Pathway | 5.27 | 12.1 | 12.71 |  |
| Tight Junction Signaling | 4.91 | 3.72 | 5.72 |  |
| Glutamate Degradation III (via 4-aminobutyrate) | 4.91 | 2.05 | 1.62 |  |
| Cytotoxic T Lymphocyte-mediated Apoptosis of Target Cells | 4.15 | 2.97 | 2.32 |  |
| Systemic Lupus Erythematosus In T Cell Signaling Pathway | 3.83 |  | 4.61 |  |
| 4-aminobutyrate Degradation I | 3.62 |  | 1.78 |  |
| MIF Regulation of Innate Immunity | 3.56 | 8.06 | 5.51 |  |
| p53 Signaling | 3.55 | 9.06 | 9.86 |  |
| Systemic Lupus Erythematosus In B Cell Signaling Pathway | 3.52 |  | 9.84 |  |
| Apelin Liver Signaling Pathway | 3.49 |  | 1.81 |  |
| IGF-1 Signaling | 3.34 | 8.84 | 1.67 |  |
| Amyloid Processing | 3.3 | 10.4 | 6.10 |  |
| Aryl Hydrocarbon Receptor Signaling | 3.14 | 13.6 | 9.66 |  |
| Unfolded protein response | 3.11 | 12 | 1.57 |  |
| EIF2 Signaling | 3.11 | 3.77 | 1.74 |  |
| Agranulocyte Adhesion and Diapedesis | 3.03 | 5.93 |  |  |
| PI3K/AKT Signaling | 2.63 | 7.42 | 3.50 |  |
| TREM1 Signaling | 2.52 | 9.53 | 7.38 |  |
| IL-17 Signaling | 2.42 | 7.37 | 6.19 |  |
| TNFR1 Signaling | 2.41 | 8.58 | 3.29 |  |
| Mitochondrial Dysfunction | 1.95 | 10.7 | 7.95 |  |
| Role of Pattern Recognition Receptors in Recognition of Bacteria and Viruses | 1.82 | 11.9 | 9.41 |  |
| HIF1α Signaling | 1.77 | 8.02 | 6.19 |  |
| Hepatic Cholestasis | 1.35 | 13 | 7.55 |  |
| Systemic Lupus Erythematosus Signaling |  | 10.1 | 2.99 |  |
| Th1 and Th2 Activation Pathway |  | 9.41 | 6.30 |  |
| Endoplasmic Reticulum Stress Pathway |  | 8.89 | 2.07 |  |
| Th1 Pathway |  | 8.85 | 4.39 |  |
| Neuroprotective Role of THOP1 in Alzheimer's Disease |  | 7.3 | 3.70 |  |
| Erythropoietin Signaling |  | 4.51 | 2.95 | 2.75 |
| *TGF-β Signaling* |  | 2.09 | 4.58 | 2.75 |
| Antiproliferative Role of Somatostatin Receptor 2 |  |  |  | 2.75 |
| ERK/MAPK Signaling |  | 6.51 | 2.14 | 2.73 |
| p70S6K Signaling |  | 6.33 | 1.68 | 2.71 |
| CNTF Signaling |  | 1.32 | 3.82 | 2.69 |
| BMP signaling pathway | 2.29 | 4.9 | 3.44 | 2.68 |
| GDNF Family Ligand-Receptor Interactions |  | 4.11 | 6.24 | 2.68 |
| Role of NFAT in Regulation of the Immune Response |  | 5.24 | 1.83 | 2.67 |
| Netrin Signaling |  | 3.56 | 3.26 | 2.63 |
| Calcium-induced T Lymphocyte Apoptosis | 1.89 | 3.56 |  | 2.55 |
| ErbB4 Signaling |  | 4.38 | 1.88 | 2.55 |
| RAR Activation |  | 6.96 | 2.56 | 2.48 |
| Acute Myeloid Leukemia Signaling | 1.5 | 2.3 | 1.95 | 2.41 |
| Relaxin Signaling | 1.75 | 3.69 | 2.78 | 2.4 |
| NF-κB Activation by Viruses |  | 3.1 | 1.57 | 2.39 |
| Superpathway of Inositol Phosphate Compounds |  |  |  | 2.33 |
| PAK Signaling | 2.04 | 2.1 | 1.80 | 2.32 |
| Prolactin Signaling |  | 5.08 | 2.79 | 2.26 |
| Thrombopoietin Signaling |  | 6.99 | 2.62 | 2.22 |
| Parkinson's Signaling | 1.75 | 6.06 | 7.87 | 2.18 |
| SAPK/JNK Signaling |  | 2.51 | 1.73 | 2.18 |
| Melanocyte Development and Pigmentation Signaling | 1.45 | 3.2 | 3.11 | 2.15 |
| FcγRIIB Signaling in B Lymphocytes |  | 2.73 | 2.26 | 2.13 |
| FAK Signaling | 1.42 | 2.13 | 1.30 | 2.1 |
| VEGF Family Ligand-Receptor Interactions |  | 5.32 | 4.04 | 2 |
| D-myo-inositol-5-phosphate Metabolism |  |  |  | 1.98 |
| IL-10 Signaling | 1.83 | 6.33 | 3.93 | 1.97 |
| IL-15 Signaling | 1.85 | 2.97 | 4.82 | 1.95 |
| JAK/Stat Signaling | 1.7 | 2.21 | 4.45 | 1.92 |
| Non-Small Cell Lung Cancer Signaling | 2.45 | 3.92 | 5.34 | 1.9 |
| PKCθ Signaling in T Lymphocytes |  | 3.18 | 1.79 | 1.88 |
| Role of IL-17F in Allergic Inflammatory Airway Diseases | 2.69 | 4.35 | 5.75 | 1.87 |
| TWEAK Signaling | 2.98 | 4.44 | 2.27 | 1.82 |
| Mechanisms of Viral Exit from Host Cells | 1.8 | 3.91 | 1.30 | 1.8 |
| IL-1 Signaling |  | 3.06 |  | 1.78 |
| Leptin Signaling in Obesity | 1.74 | 5.91 | 1.67 | 1.75 |
| Cancer Drug Resistance By Drug Efflux | 2 | 5.55 | 1.98 | 1.68 |
| Calcium Transport I | 1.87 |  |  | 1.68 |
| Antioxidant Action of Vitamin C |  | 5.25 |  | 1.67 |
| Phospholipases |  |  |  | 1.65 |
| 3-phosphoinositide Degradation |  |  |  | 1.64 |
| Regulation of Cellular Mechanics by Calpain Protease | 2.79 | 1.49 | 1.34 | 1.59 |
| Angiopoietin Signaling |  | 1.81 |  | 1.54 |
| ERK5 Signaling |  | 3.41 | 5.66 | 1.47 |
| GM-CSF Signaling |  | 3.71 | 3.22 | 1.46 |
| Oncostatin M Signaling |  | 2.45 | 3.66 | 1.45 |
| 3-phosphoinositide Biosynthesis |  |  |  | 1.44 |
| PPAR Signaling | 1.95 | 3.39 | 9.46 | 1.41 |
| Macropinocytosis Signaling |  | 5.37 | 1.71 | 1.41 |
| Actin Nucleation by ARP-WASP Complex |  |  |  | 1.32 |
| Insulin Receptor Signaling | 1.4 | 6.53 | 1.54 | 1.31 |
| mTOR Signaling |  | 2.93 |  | 1.3 |
| Dopamine Receptor Signaling | 2.94 | 6.6 |  |  |
| TR/RXR Activation | 2.94 | 4.42 | 2.59 |  |
| Prostate Cancer Signaling | 2.84 | 4.32 | 6.28 |  |
| Differential Regulation of Cytokine Production in Macrophages and T Helper Cells by IL-17A and IL-17F | 2.82 | 6.92 | 3.38 |  |
| GADD45 Signaling | 2.75 | 5.46 |  |  |
| Role of PKR in Interferon Induction and Antiviral Response | 2.73 | 4.77 | 4.77 |  |
| Remodeling of Epithelial Adherens Junctions | 2.73 | 1.35 |  |  |
| Glycolysis I | 2.69 |  |  |  |
| Chronic Myeloid Leukemia Signaling | 2.66 | 2.99 | 5.90 |  |
| BAG2 Signaling Pathway | 2.58 |  | 6.59 |  |
| Inflammasome pathway | 2.57 | 6.12 | 6.74 |  |
| Small Cell Lung Cancer Signaling | 2.57 | 5.31 | 5.61 |  |
| FAT10 Cancer Signaling Pathway | 2.54 |  | 11.32 |  |
| Sumoylation Pathway | 2.53 | 5.21 | 4.11 |  |
| IL-7 Signaling Pathway | 2.42 | 3.73 | 3.65 |  |
| Melanoma Signaling | 2.35 | 2.04 | 2.39 |  |
| HOTAIR Regulatory Pathway | 2.3 |  | 6.69 |  |
| Inhibition of ARE-Mediated mRNA Degradation Pathway | 2.29 |  | 1.41 |  |
| Glycine Degradation (Creatine Biosynthesis) | 2.25 |  |  |  |
| RANK Signaling in Osteoclasts | 2.23 | 2.77 | 3.33 |  |
| Crosstalk between Dendritic Cells and Natural Killer Cells | 2.21 | 5.2 | 4.04 |  |
| Ceramide Signaling | 2.17 | 5.84 | 9.27 |  |
| Superoxide Radicals Degradation | 2.16 | 2.28 | 4.49 |  |
| β-alanine Degradation I | 2.16 |  | 1.78 |  |
| Nur77 Signaling in T Lymphocytes | 2.12 | 1.61 | 1.47 |  |
| PFKFB4 Signaling Pathway | 2.08 |  | 6.34 |  |
| Bladder Cancer Signaling | 2.07 | 3.69 | 3.79 |  |
| Activation of IRF by Cytosolic Pattern Recognition Receptors | 2.05 | 2.62 | 6.27 |  |
| LPS/IL-1 Mediated Inhibition of RXR Function | 2 | 6.88 | 3.57 |  |
| Cell Cycle: G1/S Checkpoint Regulation | 1.96 | 1.9 | 3.38 |  |
| MIF-mediated Glucocorticoid Regulation | 1.95 | 3.4 | 2.23 |  |
| Telomerase Signaling | 1.9 |  | 2.81 |  |
| PXR/RXR Activation | 1.89 | 6.56 | 1.85 |  |
| Superpathway of Citrulline Metabolism | 1.86 | 1.83 | 2.10 |  |
| Sphingomyelin Metabolism | 1.81 |  | 1.44 |  |
| Glioma Invasiveness Signaling | 1.79 | 2.63 |  |  |
| Granzyme B Signaling | 1.75 | 2.6 | 2.42 |  |
| Citrulline-Nitric Oxide Cycle | 1.75 | 2.6 | 2.42 |  |
| Intrinsic Prothrombin Activation Pathway | 1.75 |  |  |  |
| p38 MAPK Signaling | 1.73 | 3.78 | 7.74 |  |
| Gustation Pathway | 1.73 |  | 1.32 |  |
| Role of RIG1-like Receptors in Antiviral Innate Immunity | 1.72 |  | 1.92 |  |
| Role of BRCA1 in DNA Damage Response | 1.7 |  |  |  |
| IL-15 Production | 1.69 |  | 7.59 |  |
| Histidine Degradation III | 1.66 |  |  |  |
| Gluconeogenesis I | 1.64 |  |  |  |
| Chemokine Signaling | 1.63 | 6.53 | 1.55 |  |
| iNOS Signaling | 1.62 | 2.71 | 5.29 |  |
| Cell Cycle: G2/M DNA Damage Checkpoint Regulation | 1.6 | 3.29 |  |  |
| Graft-versus-Host Disease Signaling | 1.6 | 2.01 | 3.34 |  |
| tRNA Splicing | 1.58 |  |  |  |
| Pregnenolone Biosynthesis | 1.57 | 1.49 |  |  |
| Altered T Cell and B Cell Signaling in Rheumatoid Arthritis | 1.53 | 3.87 | 4.83 |  |
| CD27 Signaling in Lymphocytes | 1.51 | 3.84 | 3.15 |  |
| Role of Cytokines in Mediating Communication between Immune Cells | 1.49 | 3.78 | 2.32 |  |
| Lymphotoxin β Receptor Signaling | 1.47 | 1.35 | 4.84 |  |
| Differential Regulation of Cytokine Production in Intestinal Epithelial Cells by IL-17A and IL-17F | 1.45 | 7.14 | 1.96 |  |
| Role of IL-17A in Arthritis | 1.45 | 4.92 | 5.75 |  |
| Hereditary Breast Cancer Signaling | 1.43 | 1.55 | 2.06 |  |
| White Adipose Tissue Browning Pathway | 1.42 |  | 8.24 |  |
| Glutamate Removal from Folates | 1.42 |  |  |  |
| MSP-RON Signaling Pathway | 1.39 | 4.79 | 4.61 |  |
| EGF Signaling | 1.39 | 4.14 | 5.55 |  |
| Role of JAK family kinases in IL-6-type Cytokine Signaling | 1.39 | 3.58 | 6.32 |  |
| Endometrial Cancer Signaling | 1.34 | 2.84 | 2.79 |  |
| Apelin Cardiomyocyte Signaling Pathway | 1.34 |  | 2.89 |  |
| Iron homeostasis signaling pathway | 1.32 | 6.58 | 8.66 |  |
| Retinoic acid Mediated Apoptosis Signaling | 1.32 | 1.97 |  |  |
| Folate Transformations I | 1.3 |  |  |  |
| Xenobiotic Metabolism Signaling |  | 6.95 | 3.49 |  |
| Inhibition of Matrix Metalloproteases |  | 6.78 |  |  |
| Granulocyte Adhesion and Diapedesis |  | 6.38 | 1.92 |  |
| Neurotrophin/TRK Signaling |  | 6.04 | 10.09 |  |
| Th2 Pathway |  | 6.04 | 2.17 |  |
| IL-17A Signaling in Gastric Cells |  | 5.63 | 3.89 |  |
| Communication between Innate and Adaptive Immune Cells |  | 5.55 | 3.09 |  |
| Role of Hypercytokinemia/hyperchemokinemia in the Pathogenesis of Influenza |  | 5.48 | 2.76 |  |
| Sonic Hedgehog Signaling |  | 4.85 |  |  |
| T Helper Cell Differentiation |  | 4.73 | 8.53 |  |
| Toll-like Receptor Signaling |  | 4.55 | 3.77 |  |
| Renal Cell Carcinoma Signaling |  | 4.51 | 7.00 |  |
| CD40 Signaling |  | 4.43 | 4.30 |  |
| Hematopoiesis from Pluripotent Stem Cells |  | 4.19 |  |  |
| Regulation of IL-2 Expression in Activated and Anergic T Lymphocytes |  | 3.96 | 2.59 |  |
| Phagosome Maturation |  | 3.6 | 6.06 |  |
| Maturity Onset Diabetes of Young (MODY) Signaling |  | 3.58 | 3.89 |  |
| Superpathway of Cholesterol Biosynthesis |  | 3.57 |  |  |
| IL-17A Signaling in Fibroblasts |  | 3.56 | 2.27 |  |
| iCOS-iCOSL Signaling in T Helper Cells |  | 3.48 | 1.50 |  |
| Estrogen-mediated S-phase Entry |  | 3.48 |  |  |
| Extrinsic Prothrombin Activation Pathway |  | 3.38 |  |  |
| Hypoxia Signaling in the Cardiovascular System |  | 3.32 | 6.54 |  |
| DNA damage-induced 14-3-3σ Signaling |  | 3.26 | 2.20 |  |
| April Mediated Signaling |  | 3.26 |  |  |
| Cholesterol Biosynthesis III (via Desmosterol) |  | 3.26 |  |  |
| CD28 Signaling in T Helper Cells |  | 3.18 | 1.85 |  |
| TNFR2 Signaling |  | 3.12 | 4.57 |  |
| B Cell Activating Factor Signaling |  | 3.12 |  |  |
| Autophagy |  | 3.11 | 6.14 |  |
| IL-17A Signaling in Airway Cells |  | 3.09 | 5.13 |  |
| ATM Signaling |  | 2.95 | 2.43 |  |
| Estrogen-Dependent Breast Cancer Signaling |  | 2.7 | 6.30 |  |
| Ephrin A Signaling |  | 2.67 |  |  |
| Cholesterol Biosynthesis I |  | 2.51 |  |  |
| Cholesterol Biosynthesis II (via 24,25-dihydrolanosterol) |  | 2.51 |  |  |
| T Cell Receptor Signaling |  | 2.26 |  |  |
| 4-1BB Signaling in T Lymphocytes |  | 2.19 | 2.41 |  |
| ErbB2-ErbB3 Signaling |  | 2.15 | 5.19 |  |
| Role of Oct4 in Mammalian Embryonic Stem Cell Pluripotency |  | 2.14 | 2.63 |  |
| FGF Signaling |  | 2.03 | 8.55 |  |
| Cyclins and Cell Cycle Regulation |  | 1.96 | 2.88 |  |
| IL-22 Signaling |  | 1.94 | 1.91 |  |
| Prostanoid Biosynthesis |  | 1.77 |  |  |
| OX40 Signaling Pathway |  | 1.68 |  |  |
| Role of p14/p19ARF in Tumor Suppression |  | 1.66 | 1.69 |  |
| Autoimmune Thyroid Disease Signaling |  | 1.41 |  |  |
| Superpathway of Geranylgeranyldiphosphate Biosynthesis I (via Mevalonate) |  | 1.39 |  |  |
| (S)-reticuline Biosynthesis II |  | 1.37 | 1.87 |  |
| Allograft Rejection Signaling |  | 1.37 |  |  |

Supplementary Table S8. Upstream regulators affected in the sTBI model compared with the upstream regulators affected by neurodegenerative diseases (based on the Ingenuity knowledge base – QIAGEN IPA, (QIAGEN Inc., https://digitalinsights.qiagen.com/IPA)

Numerical values represent -log(pvalue).

| **Upstream Regulators** | **ALS related molecules -log(p value)** | **IPA Alzheimer's disease -log(p value)** | **Neurodegeneration -log(p value)** | **sTBI - log(p value)** |
| --- | --- | --- | --- | --- |
| FGF8 |  |  | 1.72 | 1.42 |
| MDK |  | 8.45 | 5.88 |  |
| FGF9 |  | 6.17 | 2.30 |  |
| Prl3b1 |  | 5.65 | 2.53 |  |
| IL36A |  | 5.36 | 2.20 |  |
| CCL21 |  | 5.11 | 1.41 |  |
| Ptk |  | 5.02 | 4.52 |  |
| GRP |  | 4.98 | 2.15 |  |
| TNFSF15 |  | 4.98 |  |  |
| TNFSF14 |  | 4.75 | 5.32 |  |
| FGF3 |  | 4.73 | 1.69 |  |
| VAV |  | 4.50 | 2.14 |  |
| IL36G |  | 4.23 |  |  |
| CCL18 |  | 4.14 | 2.00 |  |
| SCGB1A1 |  | 3.96 | 1.94 |  |
| CXCL16 |  | 3.86 |  |  |
| CSHL1 |  | 3.61 |  |  |
| TNFSF4 |  | 3.44 | 2.58 |  |
| EBI3 |  | 3.32 |  |  |
| IL9 |  | 3.17 | 4.62 |  |
| IFNL3 |  | 2.98 | 2.40 |  |
| GMFG |  | 2.97 | 2.14 |  |
| CCL15 |  | 2.97 |  |  |
| IFNA1/IFNA13 |  | 2.87 | 2.03 |  |
| IFNL1 |  | 2.61 | 1.87 |  |
| XCL1 |  | 2.39 |  |  |
| DKK3 |  | 2.24 |  |  |
| Wnt |  | 2.17 | 2.51 |  |
| TXLNA |  | 2.07 | 2.53 |  |
| IK |  | 2.07 |  |  |
| CXCL11 |  | 2.07 |  |  |
| FGF16 |  | 2.07 |  |  |
| IFNK |  | 1.96 |  |  |
| BMP15 |  | 1.96 |  |  |
| TH1 Cytokine |  | 1.86 | 2.31 |  |
| 7S NGF |  | 1.86 | 2.31 |  |
| FGF13 |  | 1.86 |  |  |
| IFNA17 |  | 1.81 |  |  |
| CCL22 |  | 1.69 | 3.42 |  |
| CLCF1 |  | 1.69 |  |  |
| Inhibin |  | 1.56 |  |  |
| IFNW1 |  | 1.51 |  |  |
| LTBP4 |  | 1.44 | 1.88 |  |
| EDN2 |  | 1.44 | 1.88 |  |
| TH2 Cytokine |  | 1.44 | 1.88 |  |
| WNT4 |  | 1.42 |  |  |
| PDGF-CC |  | 1.41 | 1.64 |  |
| SCG2 |  | 1.41 |  |  |
| CCL23 |  | 1.41 |  |  |
| Inhibin A |  | 1.41 |  |  |
| MYDGF |  | 1.34 |  |  |
| NRTN |  | 3.08 | 2.00 | 3.09 |
| PROK1 |  | 4.14 |  | 2.31 |
| NDP |  | 1.34 | 3.06 | 2.05 |
| Ccl6 |  | 3.61 | 1.78 | 1.83 |
| AMH |  | 4.02 |  | 1.57 |
| JAG2 |  | 3.98 | 5.66 | 1.10 |
| FGF5 | 1.38 |  | 2.82 |  |
| HDGF | 1.38 |  | 2.82 |  |
| CCL24 | 1.38 |  |  |  |
| FGF6 | 1.56 |  | 1.35 |  |
| GKN1 | 1.85 |  |  |  |
| Lefty | 2.29 |  |  |  |
| AMBN | 2.55 |  |  |  |
| Igf | 2.72 |  | 2.31 |  |
| Eotaxin | 2.94 |  |  |  |
| SLURP1 | 2.94 |  |  |  |
| CXCL14 | 3.52 |  | 4.31 |  |
| IL36B | 1.32 | 5.68 | 2.65 |  |
| WNT7A | 1.32 | 1.45 | 1.74 |  |
| TH17 Cytokine | 1.38 | 4.23 |  |  |
| CCL4 | 1.39 | 5.19 | 7.62 |  |
| LTB | 1.39 | 2.88 | 3.85 |  |
| IL37 | 1.44 | 6.45 | 3.79 |  |
| IFN type 1 | 1.46 | 3.32 | 3.00 |  |
| Lymphotoxin | 1.46 | 7.43 | 2.94 |  |
| IL23 | 1.53 | 6.17 | 5.95 |  |
| JAG1 | 1.53 | 2.62 |  |  |
| EREG | 1.54 | 4.23 | 6.93 |  |
| IL17D | 1.56 | 2.24 | 1.35 |  |
| Il17d | 1.56 | 2.24 | 1.35 |  |
| IFN alpha/beta | 1.57 | 2.94 | 2.62 |  |
| Pdgf Ab | 1.59 | 3.31 | 2.13 |  |
| IFNE | 1.60 | 9.04 | 2.47 |  |
| IL19 | 1.64 | 11.55 |  |  |
| CXCL2 | 1.69 | 5.96 | 6.06 |  |
| CTF1 | 1.74 | 6.52 |  |  |
| AIMP1 | 1.74 | 10.73 | 2.37 |  |
| TNFSF9 | 1.74 | 7.60 | 3.53 |  |
| PPBP | 1.74 | 4.90 | 6.26 |  |
| TSLP | 1.80 | 7.02 |  |  |
| GDF9 | 1.80 | 6.00 |  |  |
| CYTL1 | 1.85 | 1.41 | 1.64 |  |
| REG1A | 1.85 | 1.41 | 1.64 |  |
| SOCS5 | 1.85 | 1.41 | 1.64 |  |
| Timp | 1.85 | 1.41 | 1.64 |  |
| TIMP1 | 1.86 | 13.09 | 5.94 |  |
| VGF | 1.86 | 4.06 | 2.55 |  |
| NRG4 | 1.93 | 3.06 |  |  |
| CXCL13 | 2.00 | 3.22 | 1.61 |  |
| THPO | 2.05 | 5.15 | 2.00 |  |
| IFNA2 | 2.12 | 8.60 | 9.46 |  |
| PDGF (family) | 2.18 | 5.02 | 1.78 |  |
| PRLH | 2.18 | 5.02 | 1.78 |  |
| CCL7 | 2.18 | 3.61 |  |  |
| WISP2 | 2.22 | 8.15 | 6.66 |  |
| NAMPT | 2.28 | 5.62 | 7.91 |  |
| ANGPTL3 | 2.29 | 1.44 |  |  |
| Endothelin | 2.35 | 11.00 | 7.45 |  |
| PDGFD | 2.41 | 4.50 | 3.42 |  |
| CXCL6 | 2.41 | 4.14 | 3.42 |  |
| IL17a dimer | 2.41 | 7.02 | 2.79 |  |
| PDGFC | 2.52 | 7.43 | 6.58 |  |
| CXCL3 | 2.52 | 6.19 | 6.58 |  |
| GDF15 | 2.52 | 2.98 | 4.05 |  |
| CXCL9 | 2.55 | 2.97 | 2.14 |  |
| Cxcl9 | 2.55 | 2.97 | 2.14 |  |
| FGF23 | 2.65 | 2.55 | 2.07 |  |
| CCL17 | 2.72 | 2.07 |  |  |
| PF4 | 2.76 | 11.57 | 2.85 |  |
| IL-17f dimer | 2.80 | 7.05 | 2.21 |  |
| IL12B | 2.82 | 11.31 | 11.44 |  |
| IL17F | 2.85 | 9.56 | 6.04 |  |
| CCL3L3 | 2.88 | 4.90 | 6.06 |  |
| IL23A | 2.90 | 8.57 | 7.33 |  |
| IL27 | 2.93 | 12.30 | 3.84 |  |
| IL36RN | 2.94 | 3.64 | 2.53 |  |
| OSGIN1 | 2.94 | 2.07 | 2.53 |  |
| CCL8 | 2.94 | 2.07 |  |  |
| CCL13 | 2.94 | 2.07 |  |  |
| IL17B | 2.94 | 2.07 |  |  |
| CCL19 | 2.96 | 4.90 | 2.37 |  |
| PTN | 2.96 | 1.56 | 2.37 |  |
| CXCL10 | 3.05 | 8.10 | 6.48 |  |
| C5 | 3.15 | 20.91 | 18.30 |  |
| VEGFD | 3.15 | 1.44 | 5.19 |  |
| NTN1 | 3.17 | 7.29 | 6.73 |  |
| CCL3 | 3.23 | 7.29 | 8.16 |  |
| GAS6 | 3.29 | 2.54 | 5.75 |  |
| CCL1 | 3.38 | 5.88 | 5.62 |  |
| NODAL | 3.42 | 9.11 | 2.65 |  |
| FGF4 | 3.49 | 2.70 | 1.78 |  |
| IL25 | 3.56 | 4.73 | 6.74 |  |
| PDGF-DD | 3.57 | 4.73 | 7.62 |  |
| IL21 | 3.68 | 11.02 | 11.64 |  |
| TNFSF13 | 3.69 | 8.88 | 7.94 |  |
| VAV3 | 3.80 | 3.92 | 9.44 |  |
| IL26 | 3.84 | 9.00 | 3.23 |  |
| Neurotrophin | 3.84 | 1.44 | 1.88 |  |
| Fgf | 3.86 | 4.24 | 4.86 |  |
| BTC | 4.00 | 4.38 | 2.13 |  |
| Ifn | 4.19 | 8.64 | 6.05 |  |
| Pro-inflammatory Cytokine | 4.25 | 13.99 | 7.28 |  |
| LTA | 4.32 | 3.45 | 6.03 |  |
| MST1 | 4.33 | 4.47 | 5.52 |  |
| IL17C | 4.49 | 8.89 | 4.58 |  |
| CLEC11A | 4.60 | 2.58 | 2.56 |  |
| IL12 (family) | 4.73 | 9.55 | 8.03 |  |
| TNFSF12 | 4.73 | 13.53 | 6.90 |  |
| Il8r | 4.77 | 5.59 | 5.39 |  |
| IL22 | 4.79 | 18.77 | 7.31 |  |
| NRG (family) | 4.85 | 19.14 |  |  |
| FGF10 | 4.89 | 5.23 | 2.79 |  |
| Interferon alpha | 5.01 | 12.41 | 8.66 |  |
| IL7 | 5.10 | 6.69 | 4.47 |  |
| TGFB3 | 5.29 | 9.76 | 4.21 |  |
| TNFSF13B | 5.32 | 15.09 | 4.65 |  |
| CCL5 | 5.52 | 12.29 | 12.26 |  |
| growth factor | 5.55 | 2.55 | 5.88 |  |
| CKLF | 5.57 | 4.23 | 4.94 |  |
| BMP4 | 5.62 | 13.17 | 7.61 |  |
| CCL11 | 5.62 | 10.90 | 10.07 |  |
| FGF21 | 5.62 | 4.70 | 6.88 |  |
| IL12 (complex) | 5.67 | 19.21 | 15.70 |  |
| VEGFB | 5.68 | 5.88 | 5.32 |  |
| IL11 | 5.73 | 9.65 | 3.39 |  |
| FASLG | 5.76 | 8.88 | 8.78 |  |
| PGF | 5.83 | 14.17 | 6.57 |  |
| FGF7 | 5.89 | 8.59 | 10.83 |  |
| IFN Beta | 5.94 | 8.52 | 6.61 |  |
| IL5 | 6.05 | 10.52 | 9.12 |  |
| MSTN | 6.09 | 7.08 | 6.49 |  |
| PDGF-AA | 6.11 | 10.72 | 8.85 |  |
| BMP7 | 6.20 | 7.01 | 9.41 |  |
| PDGFB | 6.30 | 15.72 | 6.39 |  |
| CSF3 | 6.37 | 23.01 | 17.42 |  |
| IFNB1 | 6.65 | 13.39 | 8.33 |  |
| IL12A | 6.65 | 12.22 | 9.50 |  |
| GRN | 7.55 | 7.31 | 11.85 |  |
| Ifn gamma | 7.69 | 13.96 | 12.88 |  |
| IL1RN | 8.07 | 15.04 | 8.66 |  |
| IL17A | 8.13 | 36.60 | 16.36 |  |
| MIF | 8.59 | 20.03 | 15.11 |  |
| IGF2 | 9.07 | 17.08 | 13.11 |  |
| IL33 | 9.30 | 14.69 | 13.69 |  |
| IL15 | 9.64 | 18.36 | 14.83 |  |
| IL18 | 9.70 | 19.27 | 15.40 |  |
| IL10 | 10.25 | 31.34 | 21.84 |  |
| HDL | 10.92 | 12.85 | 21.75 |  |
| IL24 | 11.34 | 12.55 | 7.81 |  |
| GHRL | 11.49 | 15.25 | 9.77 |  |
| IL1A | 11.72 | 29.93 | 19.51 |  |
| TNFSF10 | 11.75 | 21.60 | 18.00 |  |
| PRL | 12.19 | 29.40 | 15.79 |  |
| FGF19 | 12.97 | 8.95 | 3.73 |  |
| cytokine | 12.99 | 28.14 | 23.90 |  |
| IL32 | 14.71 | 22.34 | 14.15 |  |
| CSF2 | 14.86 | 26.13 | 26.09 |  |
| IL13 | 15.18 | 26.75 | 16.90 |  |
| SPP1 | 20.19 | 32.90 | 28.55 |  |
| IL1 | 20.61 | 45.03 | 32.61 |  |
| BDNF | 28.94 | 39.50 | 35.35 | 11.42 |
| PDGF BB | 19.52 | 30.13 | 22.70 | 11.09 |
| TGFB1 | 27.55 | 70.72 | 38.17 | 10.73 |
| WNT3A | 7.55 | 16.10 | 9.94 | 8.43 |
| Vegf | 4.88 | 28.17 | 14.02 | 7.54 |
| NGF | 15.66 | 39.95 | 25.66 | 7.54 |
| Ngf | 15.66 | 39.95 | 25.66 | 7.54 |
| TNF | 37.81 | 76.83 | 51.32 | 7.11 |
| FGF1 | 4.03 | 13.94 | 11.76 | 5.75 |
| IFNG | 16.53 | 65.67 | 49.20 | 5.41 |
| GDF2 | 2.02 | 10.60 | 4.35 | 5.16 |
| INHA | 4.67 | 8.25 | 2.49 | 5.14 |
| IGF1 | 24.06 | 53.93 | 38.10 | 4.88 |
| AGT | 20.75 | 45.84 | 39.53 | 4.53 |
| HGF | 11.74 | 30.13 | 18.90 | 4.49 |
| FGF2 | 25.31 | 50.96 | 29.08 | 4.38 |
| CXCL12 | 12.84 | 19.58 | 11.11 | 4.12 |
| LIF | 6.94 | 19.05 | 12.64 | 3.95 |
| EGF | 25.49 | 47.92 | 32.50 | 3.89 |
| GDNF | 10.52 | 14.62 | 7.97 | 3.57 |
| Pdgf (complex) | 5.57 | 24.95 | 16.76 | 3.42 |
| INHBA | 6.07 | 16.50 | 4.31 | 3.37 |
| ANGPT2 | 11.29 | 19.58 | 14.53 | 3.37 |
| NRG1 | 11.14 | 21.38 | 17.65 | 3.35 |
| KITLG | 8.16 | 18.05 | 13.93 | 3.33 |
| EDN1 | 10.24 | 26.56 | 14.32 | 3.10 |
| NRG2 | 2.66 | 5.18 | 6.66 | 2.97 |
| VEGFA | 10.65 | 27.96 | 18.80 | 2.92 |
| WNT1 | 6.44 | 6.84 | 3.06 | 2.72 |
| OSM | 10.78 | 22.02 | 8.72 | 2.67 |
| HBEGF | 4.43 | 5.26 | 9.63 | 2.51 |
| IL4 | 14.54 | 30.78 | 22.93 | 2.47 |
| TNFSF11 | 10.36 | 13.15 | 14.40 | 2.44 |
| BMP2 | 7.13 | 15.58 | 6.26 | 2.40 |
| IL1B | 22.58 | 72.77 | 45.73 | 2.39 |
| BMP6 | 3.21 | 7.36 | 3.93 | 2.24 |
| CNTF | 9.47 | 18.88 | 15.21 | 2.21 |
| CX3CL1 | 6.71 | 18.51 | 10.39 | 2.21 |
| CSF1 | 12.50 | 17.98 | 14.53 | 2.13 |
| Tgf beta | 13.72 | 30.64 | 24.22 | 2.09 |
| DKK1 | 6.35 | 9.04 | 3.06 | 2.07 |
| NTF3 | 3.17 | 8.29 | 6.73 | 1.99 |
| FAM3B | 1.39 | 4.73 |  | 1.87 |
| EPO | 11.86 | 24.71 | 16.95 | 1.86 |
| IL3 | 4.41 | 11.91 | 7.43 | 1.77 |
| Il3 | 4.41 | 11.91 | 7.43 | 1.77 |
| CCL2 | 9.22 | 26.93 | 13.69 | 1.63 |
| Ccl2 | 9.22 | 26.93 | 13.69 | 1.63 |
| LEP | 27.97 | 57.71 | 27.70 | 1.63 |
| MAC | 4.25 | 3.13 | 4.31 | 1.57 |
| IL2 | 9.74 | 17.45 | 17.78 | 1.57 |
| VEGFC | 2.00 | 3.22 | 1.61 | 1.50 |
| NOV | 3.57 | 3.72 |  | 1.43 |
| CRH | 6.51 | 20.67 | 19.30 | 1.43 |
| ANGPT1 | 4.32 | 4.48 | 6.03 | 1.41 |
| CD40LG | 12.91 | 20.78 | 14.92 | 1.39 |
| CXCL1 | 1.80 | 6.45 | 3.66 | 1.36 |
| GH1 | 17.43 | 12.75 | 7.61 | 1.34 |
| CXCL8 | 6.18 | 14.15 | 6.80 | 1.31 |
| TGFA | 7.59 | 14.15 | 18.19 | 1.31 |
| WNT5A | 9.16 | 22.70 | 15.41 | 1.24 |
| Growth hormone | 14.79 | 22.36 | 8.57 | 1.19 |
| NOG | 2.99 | 2.37 | 4.50 | 1.16 |
| IL6 | 21.92 | 69.23 | 36.24 | 1.13 |
| CTGF | 7.41 | 14.35 | 8.54 | 0.78 |
| TGFB2 | 3.69 | 15.30 | 4.24 | 0.76 |
| AREG | 2.06 | 4.37 | 3.69 | 0.47 |
| Tnf (family) | 4.16 | 17.24 | 15.80 | 0.28 |
| NTF4 | 1.64 | 3.44 |  | 2.14 |

Supplementary Table S9a. Cellular functions affected in the sTBI model compared with the cellular functions affected by neurodegenerative diseases (based on the Ingenuity knowledge base – QIAGEN IPA, (QIAGEN Inc., https://digitalinsights.qiagen.com/IPA)

Numerical values represent -log(pvalue).

| **Functions** | **ALS related molecules -log(p value)** | **IPA Alzheimer's disease -log(p value)** | **Neurodegeneration -log(p value)** | **sTBI -log(p value)** |
| --- | --- | --- | --- | --- |
| Metabolism of reactive oxygen species |  | 87.25 | 72.29 |  |
| Synthesis of reactive oxygen species |  | 84.20 | 71.06 |  |
| Behavior |  | 130.70 |  | 38.49 |
| Morphogenesis of neurons |  | 52.13 | 76.86 | 31.42 |
| Neuritogenesis |  | 52.12 | 76.61 | 30.49 |
| Cell movement |  | 106.24 | 66.90 | 24.21 |
| Migration of cells |  | 102.07 | 65.30 | 23.07 |
| Proliferation of neuronal cells |  | 59.50 | 82.23 | 19.23 |
| Shape change of neurites |  | 48.22 | 47.25 | 19.21 |
| Angiogenesis |  | 74.96 | 51.77 | 17.91 |
| Cell viability |  | 87.73 | 103.04 | 11.43 |
| Cell survival |  | 88.63 | 106.00 | 10.67 |
| Cellular degradation | 34.36 | 63.49 |  |  |
| Degeneration of neurons | 33.84 | 62.05 |  |  |
| **development of neurons** | 28.36 | 59.70 | 97.10 | 39.55 |
| **Organization of cytoskeleton** | 29.00 | 65.24 | 75.47 | 36.79 |
| **Organization of cytoplasm** | 29.48 | 64.07 | 88.99 | 33.70 |
| **Morphology of nervous system** | 31.24 | 92.77 | 217.58 | 33.45 |
| **Neurotransmission** | 41.91 | 84.88 | 56.94 | 31.75 |
| **Microtubule dynamics** | 27.03 | 58.55 | 74.86 | 30.56 |
| **Learning** | 37.32 | 98.11 | 84.04 | 30.20 |
| **Synaptic transmission** | 32.69 | 80.59 | 51.78 | 27.75 |
| **Morphology of neurons** | 30.58 | 73.35 | 183.73 | 23.37 |
| **Long-term potentiation** | 25.97 | 67.16 | 53.68 | 22.19 |
| **Vasculogenesis** | 25.47 | 77.05 | 62.47 | 20.02 |
| **Apoptosis** | 36.07 | 127.31 | 124.97 | 19.00 |
| **Memory** | 30.86 | 77.35 | 60.86 | 18.89 |
| **Necrosis** | 47.59 | 144.20 | 139.14 | 18.42 |
| **Emotional behavior** | 69.49 | 56.49 | 39.05 | 16.52 |
| **Neuronal cell death** | 43.65 | 125.33 | 222.95 | 13.19 |
| **Transport of molecule** | 47.13 | 102.58 | 79.38 | 13.10 |
| **Concentration of lipid** | 28.60 | 82.31 | 70.70 | 12.56 |
| **Cellular homeostasis** | 36.84 | 105.56 | 105.67 | 12.43 |
| **Locomotion** | 29.54 | 61.58 | 86.36 | 12.20 |
| Transport of ion | 29.08 |  |  | 10.46 |
| Transport of metal | 29.98 |  | 38.25 | 8.21 |
| Activation of cells |  | 71.28 | 71.10 |  |
| Fatty acid metabolism |  | 68.97 | 51.26 |  |
| Quantity of steroid |  | 66.14 | 46.08 |  |
| Production of reactive oxygen species |  | 62.93 | 69.48 |  |
| Endocytosis |  | 59.99 | 37.21 |  |
| Immune response of brain |  | 58.64 | 38.96 |  |
| Metabolism of protein |  | 58.60 | 40.36 |  |
| Growth of muscle tissue |  | 57.37 |  |  |
| Insulin resistance |  | 56.91 |  |  |
| Concentration of sterol |  | 52.24 |  |  |
| Synthesis of fatty acid |  | 51.07 | 47.70 |  |
| Concentration of cholesterol |  | 50.12 |  |  |
| Autophagy |  | 49.56 | 48.91 |  |
| Interaction of DNA |  | 48.78 | 45.62 |  |
| Binding of DNA |  | 48.70 | 46.68 |  |
| Metabolism of membrane lipid derivative |  | 48.35 |  |  |
| Synthesis of DNA |  | 48.22 | 42.73 |  |
| Export of molecule |  | 47.11 |  |  |
| Stress response of cells |  | 46.76 | 40.77 |  |
| Concentration of fatty acid |  | 46.55 | 39.57 |  |
| Growth of neurites |  | 57.40 | 72.11 | 18.02 |
| Outgrowth of neurites |  | 51.17 | 55.87 | 15.23 |
| Differentiation of connective tissue cells |  | 47.04 | 48.60 | 13.19 |
| Quantity of nervous tissue |  | 51.70 | 112.20 | 12.94 |
| Quantity of neurons |  | 51.17 | 111.33 | 12.45 |
| Synthesis of nitric oxide |  | 47.32 | 49.19 | 11.44 |
| Cell cycle progression |  | 47.68 |  | 11.21 |
| Quantity of carbohydrate |  | 48.96 | 44.12 | 11.21 |
| Morphology of muscle |  | 53.84 | 61.61 | 10.53 |
| Invasion of cells |  | 64.21 | 44.48 | 10.41 |
| Synthesis of lipid |  | 73.03 | 54.97 | 10.02 |
| Quantity of metal |  | 57.22 | 54.38 | 7.87 |
| Proliferation of muscle cells |  | 55.02 | 44.98 | 7.54 |
| Quantity of Ca2+ |  | 49.50 | 50.84 | 7.36 |
| Size of cells |  | 51.62 | 69.58 | 7.31 |
| Quantity of metal ion |  | 50.86 | 52.94 | 6.99 |
| Inflammation of nervous system | 26.82 |  | 49.51 |  |
| Ion homeostasis of cells | 26.72 | 55.27 | 60.88 |  |
| Transmembrane potential | 26.18 | 54.70 | 48.87 |  |
| Sprouting | 26.94 | 57.56 | 49.74 | 17.02 |
| Branching of cells | 26.17 | 52.10 | 50.71 | 15.63 |
| Secretion of molecule | 26.96 | 65.09 | 56.43 | 10.54 |
| Cell death of muscle cells | 27.49 | 53.19 | 50.44 | 8.17 |
| Muscle contraction | 25.50 |  |  | 8.70 |
| Dendritic growth/branching |  |  |  | 18.44 |
| Conditioning |  |  |  | 16.58 |
| Excitatory postsynaptic potential |  |  |  | 13.20 |
| Transcription of RNA |  |  |  | 12.00 |
| Concentration of hormone |  |  |  | 10.79 |
| Differentiation of nervous system |  |  | 55.17 | 10.43 |
| Long term depression |  |  |  | 9.75 |
| Axonogenesis |  |  | 45.08 | 9.24 |
| Innervation |  |  | 37.93 | 8.90 |
| Coordination |  |  | 74.08 | 8.81 |
| Phosphorylation of protein |  |  |  | 8.65 |
| Transactivation of RNA |  |  |  | 8.56 |
| Long-term memory |  |  |  | 8.22 |
| Metabolism of hormone |  |  |  | 8.20 |
| Collapse of growth cone |  |  |  | 8.07 |
| Action potential of neurons |  |  |  | 8.01 |
| Growth of axons |  |  | 37.25 | 7.81 |
| Quantity of connective tissue |  |  | 43.64 | 7.36 |
| Oscillation of Ca2+ |  |  |  | 7.28 |
| Function of smooth muscle |  |  |  | 7.07 |

Supplementary Table S9b. Diseases with similarly affected gene expression changes to the sTBI model compared with the diseases with similar expression signatures to neurodegenerative diseases (based on the Ingenuity knowledge base – QIAGEN IPA, (QIAGEN Inc., https://digitalinsights.qiagen.com/IPA)

Numerical values represent -log(pvalue).

| **Diseases** | **ALS related molecules -log(p value)** | **IPA Alzheimer's disease -log(p value)** | **Neurodegeneration -log(p value)** | **sTBI -log(p value)** |
| --- | --- | --- | --- | --- |
| Huntington's Disease | 100.90 | 106.20 | 36.43 | 9.79 |
| Occlusion of blood vessel |  | 122.80 | 39.62 | 7.89 |
| Chorea |  | 105.88 | 36.31 | 10.22 |
| Severe psychological disorder | 95.65 |  | 42.74 |  |
| Neuromuscular disease | 110.68 | 186.84 | 66.08 |  |
| Drug dependence | 98.20 | 91.92 |  |  |
| Headache | 95.64 | 71.87 |  |  |
| Schizoaffective disorder | 94.21 | 108.85 |  |  |
| Stroke | 93.54 | 115.35 |  |  |
| Damage of nervous system | 91.00 | 134.49 | 121.31 |  |
| Injury of nervous system | 89.19 | 112.62 | 107.36 |  |
| Parkinson's disease | 85.37 | 114.30 | 53.38 |  |
| Inflammation of organ | 69.05 | 208.64 | 73.34 |  |
| Chronic inflammatory disorder | 52.38 | 134.46 | 37.36 |  |
| **Amyotrophic lateral sclerosis** | 126.61 | 97.89 | 37.49 | 8.84 |
| **Neurological signs** | 109.82 | 155.04 | 77.12 | 15.08 |
| **Disorder of basal ganglia** | 109.16 | 151.28 | 92.71 | 9.44 |
| **Anxiety Disorders** | 109.01 | 95.99 |  | 7.09 |
| **Movement Disorders** | 108.95 | 178.43 | 165.77 | 13.50 |
| **Dyskinesia** | 103.45 | 132.16 | 46.49 | 11.27 |
| **Major depression** | 100.20 | 94.55 |  | 8.52 |
| **Discomfort** | 99.60 | 123.84 |  | 7.46 |
| **Depressive disorder** | 99.59 | 122.03 |  | 10.48 |
| **Cerebrovascular dysfunction** | 94.11 | 147.58 |  | 7.70 |
| **Mood Disorders** | 94.10 | 132.69 |  | 14.22 |
| **Schizophrenia spectrum disorder** | 89.80 | 147.45 |  | 11.01 |
| **Major affective disorder** | 84.93 | 109.55 |  | 14.20 |
| **Bipolar disorder** | 75.29 | 83.31 |  | 12.04 |
| **Cognitive impairment** | 71.25 | 95.82 | 49.33 | 14.22 |
| **Glucose metabolism disorder** | 69.88 | 157.38 | 56.80 | 9.63 |
| **Seizure disorder** | 62.01 | 81.01 | 57.30 | 16.43 |
| **Epilepsy** | 56.72 | 57.25 |  | 13.70 |
| **Organismal death** | 44.70 | 105.92 | 122.33 | 30.57 |
| **abnormal morphology of neurons** | 30.26 | 60.53 | 161.51 | 18.68 |
| Tauopathy | 126.61 |  | 79.92 | 13.50 |
| Dementia | 114.85 |  | 85.28 | 12.78 |
| Amyloidosis | 106.30 |  | 84.33 | 11.96 |
| Alzheimer disease | 105.37 |  | 77.86 | 13.21 |
| Autism or intellectual disability | 57.57 |  |  | 10.38 |
| Substance-Related Disorders |  | 98.43 |  |  |
| Addiction |  | 92.79 |  |  |
| Neuropathic pain |  | 74.63 |  |  |
| Ischemia of central nervous system |  | 62.40 |  |  |
| Encephalitis |  | 58.00 | 39.11 |  |
| Cerebral ischemia |  | 56.72 |  |  |
| Personality disorder |  | 52.33 |  |  |
| Vascular cognitive impairment |  | 47.71 |  |  |
| Familial Alzheimer disease |  | 47.30 |  |  |
| Frontotemporal lobar degeneration or amyotrophic lateral sclerosis | 37.99 | 104.34 | 41.93 | 9.56 |
| Cerebral degeneration |  | 108.64 | 47.23 | 9.66 |
| Irritable behavior | 91.89 |  |  |  |
| Musculoskeletal pain | 77.30 |  |  |  |
| Familial amyotrophic lateral sclerosis | 76.70 |  |  |  |
| First episode psychosis | 70.71 |  |  |  |
| Refractory schizophrenia | 70.71 |  |  |  |
| Recurrent mania | 68.92 |  |  |  |
| Acute pain | 64.24 |  |  |  |
| Fatigue | 61.86 |  |  |  |
| Non-cancer pain | 61.43 |  |  |  |
| Early-onset schizophrenia | 55.50 |  |  |  |
| Diabetic neuropathy | 53.72 |  |  |  |
| Tourette syndrome | 53.70 |  |  |  |
| Fragile X syndrome | 53.32 |  |  |  |
| Generalized anxiety disorder | 51.67 |  |  |  |
| Partial seizure | 51.37 |  |  |  |
| Non-Alzheimer degenerative dementia | 46.66 |  |  |  |
| Stuttering | 46.00 |  |  |  |
| Hereditary neuropathy | 45.45 |  | 49.15 |  |
| Unconsciousness | 44.16 |  |  |  |
| Complex partial seizure | 44.08 |  |  |  |
| Localization-related epilepsy | 43.56 |  |  |  |
| Status epilepticus | 42.37 |  |  |  |
| Single major depressive episode | 40.71 |  |  |  |
| Syndromic X-linked mental retardation | 40.27 |  |  |  |
| Skelatal muscle spasticity | 39.35 |  |  |  |
| Ataxia | 38.52 |  | 89.34 |  |
| Degenerative ataxia | 38.49 |  |  |  |
| Lennox-Gastaut syndrome | 38.47 |  |  |  |
| Chronic pain | 38.30 |  |  |  |
| Refractory epilepsy | 38.17 |  |  |  |
| Refractory seizures | 37.99 |  |  |  |
| Tension headaches | 36.96 |  |  |  |
| Spinocerebellar ataxia type 2 | 36.74 |  |  |  |
| Early-onset encephalopathy | 36.56 |  |  |  |
| Multiple system atrophy | 36.41 |  |  |  |
| Motor seizure | 35.72 |  |  |  |
| Convulsion | 35.16 |  |  |  |
| Generalized seizures | 34.55 |  |  |  |
| Tonic-clonic seizure | 34.54 |  |  |  |
| abnormal metabolism | 34.18 |  | 38.04 |  |
| Postpartum depression | 33.38 |  |  |  |
| Familial syndromic intellectual disability | 33.38 |  |  |  |
| Suicidal ideation | 31.92 |  |  |  |
| Pediatric-onset neurological disease | 30.48 |  |  |  |
| Hereditary myopathy | 30.46 |  |  |  |
| Complex insomnia | 30.40 |  |  |  |
| Childhood encephalopathy | 30.23 |  |  |  |
| Primary insomnia | 29.13 |  |  |  |
| Lewy body disease | 28.95 |  |  |  |
| Delusional disorder | 28.58 |  |  |  |
| Cramp of muscle | 28.56 |  |  |  |
| Binge eating disorder | 28.35 |  |  |  |
| Nicotine dependence | 27.61 |  |  |  |
| Treatment-resistant major depressive disorder | 27.43 |  |  |  |
| Peripheral neuropathy | 27.09 |  |  |  |
| Familial epilepsy | 27.00 |  |  |  |
| Childhood epilepsy | 26.81 |  |  |  |
| Chronic insomnia | 26.74 |  |  |  |
| Autosomal dominant motor neuron disease | 26.53 |  |  |  |
| Autosomal dominant neuropathy | 26.48 |  |  |  |
| Hydrocephalus | 26.39 |  |  |  |
| Asperger syndrome | 25.98 |  |  |  |
| Benign seizure | 25.47 |  |  |  |
| Bipolar spectrum disorder | 93.78 | 74.29 |  |  |
| Alcoholism | 93.18 | 88.42 |  |  |
| Social anxiety disorder | 92.74 | 50.17 |  |  |
| Bipolar depression | 91.68 | 78.83 |  |  |
| Migraines | 90.47 | 67.38 |  |  |
| Treatment resistant depression | 87.81 | 73.06 |  |  |
| Agitation | 87.72 | 68.83 |  |  |
| Acute encephalopathy | 79.70 | 88.50 |  |  |
| Non-affective psychosis | 78.97 | 82.08 |  |  |
| Delirium | 77.88 | 84.23 |  |  |
| Extrapyramidal syndrome | 74.54 | 57.34 |  |  |
| Obsessive-compulsive spectrum disorder | 72.02 | 63.03 |  |  |
| Post-traumatic stress disorder | 71.01 | 75.31 |  |  |
| Mania | 70.40 | 59.06 |  |  |
| Opioid dependence | 69.12 | 56.61 |  |  |
| Relapsed schizophrenia | 68.16 | 65.86 |  |  |
| Panic disorder | 67.43 | 61.63 |  |  |
| Multiple Sclerosis | 67.22 | 107.96 |  |  |
| Attention deficit hyperactivity disorder | 66.91 | 67.87 |  |  |
| Sleep Disorders | 66.82 | 77.55 |  |  |
| Impulse control disorder | 66.03 | 52.46 |  |  |
| Trichotillomania | 64.98 | 52.93 |  |  |
| Borderline personality disorder | 64.96 | 50.99 |  |  |
| Syndromic encephalopathy | 64.86 | 60.78 |  |  |
| Insomnia | 63.97 | 71.95 |  |  |
| Fibromyalgia | 63.80 | 48.48 |  |  |
| Pain of muscle | 63.51 | 54.18 |  |  |
| Dyssomnia | 63.23 | 76.18 |  |  |
| Eating Disorders | 61.78 | 69.92 |  |  |
| Tardive dyskinesia | 61.45 | 53.35 |  |  |
| Anorexia nervosa | 61.20 | 56.54 |  |  |
| Phobic anxiety disorder | 58.43 | 48.74 |  |  |
| Speech and language disorders | 56.82 | 46.53 |  |  |
| Early-onset neurological disorder | 56.40 | 50.74 |  |  |
| Myoclonus | 49.00 | 56.73 |  |  |
| Systemic inflammatory response syndrome and/or sepsis | 46.75 | 72.56 |  |  |
| Relapsing-remitting multiple sclerosis | 45.20 | 47.21 |  |  |
| Relapsed multiple sclerosis | 42.80 | 55.58 |  |  |
| Systemic autoimmune syndrome | 42.03 | 98.71 |  |  |
| Psychomotor agitation | 41.90 | 60.11 |  |  |
| Ischemic stroke | 39.21 | 58.09 |  |  |
| Subarachnoid hemorrhage | 37.96 | 47.46 |  |  |
| Disorder of lipid metabolism | 36.65 | 63.41 |  |  |
| Mild cognitive impairment | 35.74 | 76.65 |  |  |
| Cannabis dependence | 35.18 | 54.49 |  |  |
| Degeneration of nervous system | 34.84 | 65.67 | N/A |  |
| Tobacco-related disorder | 30.90 | 48.71 |  |  |
| Organ Degeneration | 30.52 | 61.76 | 309.60 |  |
| Hyperlipidemia | 29.83 | 52.06 |  |  |
| Infarction of cerebrum | 28.55 | 54.14 |  |  |
| Psychosis | 76.52 | 86.53 |  | 8.31 |
| Neurodegeneration | 37.49 | 71.58 | 300.00 | 7.62 |
| abnormal morphology of nervous system | 29.43 | 73.83 | 203.64 | 9.43 |
| Mental retardation | 49.92 |  |  | 9.25 |
| Hyperesthesia | 27.66 |  | 38.96 | 7.98 |
| Sensory disorders | 27.03 |  | 41.47 | 9.16 |
| Familial mental retardation | 25.53 |  |  | 7.20 |
| abnormal morphology of vasculature |  |  |  | 7.59 |
| Hyperactive behavior |  |  |  | 7.43 |
| Fear |  |  |  | 7.42 |

Supplementary Table S11. Canonical pathways affected by ILB^®^ in the sTBI model.

Numerical values are the activation z scores (z>0 activation; z<0 inhibition) of the pathways based on the differential gene expression in the different data-sets.

| **Canonical Pathways** | **sTBI** | **sTBI ILB^®^ 1mg/kg** | **sTBI ILB^®^ 5mg/kg** | **sTBI ILB^®^ 15mg/kg** |
| --- | --- | --- | --- | --- |
| CREB Signaling in Neurons | -5.076 | 3.618 | 3.883 | 3.244 |
| Opioid Signaling Pathway | -3.048 | 3.3 | 3.064 | 2.63 |
| Synaptic Long Term Depression | -4.061 | 3.841 | 3.845 | 3.654 |
| Synaptic Long Term Potentiation | -3.479 | 3.507 | 3.883 | 2.832 |
| GNRH Signaling | -3.592 | 3.92 | 4.106 | 3.394 |
| CXCR4 Signaling | -1.333 | 1.441 | 0.885 | 0.762 |
| Neuropathic Pain Signaling In Dorsal Horn Neurons | -3.307 | 3.162 | 3.727 | 2.846 |
| Cholecystokinin/Gastrin-mediated Signaling | -1.219 | 1.333 | 1.718 | 0.949 |
| Calcium Signaling | -3.781 | 4.423 | 3.727 | 3.479 |
| Osteoarthritis Pathway | -1.64 | 1.664 | 0.98 | 1.361 |
| Endothelin-1 Signaling | -0.885 | 0.98 | 1.477 | 1.336 |
| Cardiac Hypertrophy Signaling | -2.598 | 3.151 | 3.3 | 1.921 |
| Glutamate Receptor Signaling | -2.53 | 2.53 | 2.111 | 1.897 |
| GPCR-Mediated Nutrient Sensing in Enteroendocrine Cells | -2.121 | 2.667 | 3.086 | 2.401 |
| Actin Cytoskeleton Signaling | -2.832 | 3.266 | 3.571 | 2.661 |
| UVC-Induced MAPK Signaling | -1.147 | 1.606 | 1.46 | 1.043 |
| Dopamine-DARPP32 Feedback in cAMP Signaling | -2.611 | 3.28 | 3.355 | 2.082 |
| nNOS Signaling in Neurons | -1.89 | 2.333 | 2.121 | 1.134 |
| Huntington's Disease Signaling | -1.414 | 0.469 | 1.64 | 1.897 |
| cAMP-mediated signaling | -2.722 | 3.394 | 1.604 | 2.143 |
| Neuroinflammation Signaling Pathway | -1.543 | 1.987 | 3.311 | 2.177 |
| Gαs Signaling | -1.964 | 2.502 | 2.043 | 2.041 |
| eNOS Signaling | -1.877 | 2.043 | 1.567 | 2.041 |
| Amyotrophic Lateral Sclerosis Signaling | -1.5 | 1.414 | 1.46 | 1.706 |
| Acute Phase Response Signaling | -1.877 | 1.89 | 2.535 | 2.197 |
| Role of NFAT in Cardiac Hypertrophy | -3.015 | 3.709 | 3.713 | 2.782 |
| Phospholipase C Signaling | -2.534 | 1.697 | 2.138 | 2.023 |
| Agrin Interactions at Neuromuscular Junction | -2.065 | 2.4 | 2.294 | 2.711 |
| Aldosterone Signaling in Epithelial Cells | -2.043 | 2.611 | 2.92 | 1.915 |
| Protein Kinase A Signaling | -1.524 | 2.038 | 1.706 | 1.287 |
| PTEN Signaling | 2.828 | -2.744 | -3.244 | -3 |
| G Beta Gamma Signaling | -3.413 | 3.55 | 3.683 | 3.333 |
| Wnt/β-catenin Signaling | 0.686 | -1.234 | -1.151 | -0.667 |
| Thrombin Signaling | -1.715 | 1.372 | 1.857 | 1.206 |
| Corticotropin Releasing Hormone Signaling | -1.414 | 1.121 | 1.372 | 1.151 |
| Glioblastoma Multiforme Signaling | -1.183 | 0.617 | 1.769 | 0.469 |
| Tec Kinase Signaling | -0.928 | 1.768 | 1.897 | 1.372 |
| IL-8 Signaling | -1.718 | 1.474 | 1.54 | 1.313 |
| Ephrin Receptor Signaling | -4.004 | 4 | 4.333 | 3.363 |
| Colorectal Cancer Metastasis Signaling | -1.043 | 0.555 | 1.192 | 0.832 |
| PPARα/RXRα Activation | 2.335 | -1.808 | -1.508 | -1.622 |
| Neuregulin Signaling | -2.558 | 2.887 | 3.536 | 2.921 |
| Ephrin B Signaling | -2.668 | 2.683 | 2.683 | 2.357 |
| GP6 Signaling Pathway | -2.694 | 2.667 | 3.904 | 3.087 |
| UVA-Induced MAPK Signaling | -2.357 | 1.886 | 2.558 | 2.236 |
| Signaling by Rho Family GTPases | -2.16 | 3.201 | 2.582 | 2.138 |
| **Pyridoxal 5'-phosphate Salvage Pathway** | -1.789 | 1.091 | 1.091 | 1.147 |
| ErbB Signaling | -2.6 | 3 | 2.921 | 2.828 |
| α-Adrenergic Signaling | -2.357 | 2.4 | 2.132 | 1.789 |
| Fcγ Receptor-mediated Phagocytosis in Macrophages and Monocytes | 0.6 | 0.18 | 1.414 | -0.186 |
| Renin-Angiotensin Signaling | -2.353 | 2.268 | 2.197 | 1.915 |
| RhoGDI Signaling | 1.976 | -2.236 | -1.581 | -1.581 |
| GPCR-Mediated Integration of Enteroendocrine Signaling Exemplified by an L Cell | 0.218 | 0.408 | -0.539 | -0.218 |
| HGF Signaling | -2.858 | 2.887 | 3.781 | 2.744 |
| Gαq Signaling | -2.121 | 1.982 | 2.48 | 1.718 |
| 14-3-3-mediated Signaling | -0.784 | 1.826 | 2.402 | 1.667 |
| P2Y Purigenic Receptor Signaling Pathway | -2.117 | 2.117 | 3 | 2.335 |
| PCP pathway | -0.243 | 0 | 0.229 | -0.943 |
| Thyroid Cancer Signaling | -2.828 | N/A | N/A | N/A |
| Melatonin Signaling | -0.471 | 0.447 | 0.784 | 0.209 |
| Mouse Embryonic Stem Cell Pluripotency | -2.2 | 0.928 | 1.4 | 1.8 |
| IL-3 Signaling | -2.4 | 2.711 | 2.887 | 2.646 |
| **Nitric Oxide Signaling in the Cardiovascular System** | -2.711 | 2.828 | 2.121 | 1.961 |
| Integrin Signaling | -2.596 | 2.021 | 3.111 | 2.496 |
| Androgen Signaling | -2.065 | 2.683 | 2.4 | 2.4 |
| Paxillin Signaling | -3.3 | 3.578 | 3 | 3.411 |
| NGF Signaling | -2.746 | 2.556 | 3.244 | 2.475 |
| Adrenomedullin signaling pathway | -1.761 | 1.605 | 2.101 | 1.474 |
| Fc Epsilon RI Signaling | -0.392 | 1.177 | 1.219 | 0.18 |
| FLT3 Signaling in Hematopoietic Progenitor Cells | -2.837 | 3.411 | 3 | 2.858 |
| fMLP Signaling in Neutrophils | -2.2 | 2.414 | 2.414 | 1.616 |
| Ovarian Cancer Signaling | -3.317 | 2.138 | 1.807 | 2.324 |
| VDR/RXR Activation | 0.775 | -0.894 | -1.807 | -1.213 |
| Leukocyte Extravasation Signaling | -2.535 | 4.117 | 4.899 | 2.777 |
| Salvage Pathways of Pyrimidine Ribonucleotides | -1.46 | 0 | 0.6 | 1.043 |
| Role of NANOG in Mammalian Embryonic Stem Cell Pluripotency | -2.714 | 2.887 | 2.887 | 2.714 |
| Wnt/Ca+ pathway | -1.698 | 0.688 | 1.964 | 1.414 |
| Type II Diabetes Mellitus Signaling | -0.243 | 1.706 | 1.89 | 0.962 |
| Rac Signaling | -4.2 | 4.271 | 3.922 | 3.651 |
| CCR3 Signaling in Eosinophils | -2.236 | 1.789 | 1.4 | 1.961 |
| CCR5 Signaling in Macrophages | 0 | 0.832 | 0.728 | 0 |
| Sperm Motility | -1.961 | 2.556 | 2.596 | 1.915 |
| Regulation of Actin-based Motility by Rho | -0.218 | 0.378 | 0.426 | -0.392 |
| Growth Hormone Signaling | -1.698 | 1.528 | 2.558 | 1.706 |
| B Cell Receptor Signaling | -2.959 | 3.363 | 4.23 | 2.832 |
| PI3K Signaling in B Lymphocytes | -2.887 | 3.053 | 4.111 | 3.569 |
| Antiproliferative Role of Somatostatin Receptor 2 | -2.887 | 3.051 | 2.673 | 2.496 |
| TGF-β Signaling | -1.886 | 2.132 | 2.294 | 1.46 |
| Erythropoietin Signaling | -1.183 | N/A | N/A | N/A |
| ERK/MAPK Signaling | -3.43 | 3.124 | 3.507 | 3.202 |
| p70S6K Signaling | -2.746 | 2.414 | 3.333 | 2.744 |
| CNTF Signaling | -3.357 | 2.5 | 2.668 | 2.5 |
| BMP signaling pathway | -2.183 | 1.964 | 2.132 | 1.279 |
| GDNF Family Ligand-Receptor Interactions | -2 | 1.807 | 1.789 | 2 |
| Role of NFAT in Regulation of the Immune Response | -2.646 | 2.414 | 4.217 | 3.773 |
| Glioma Signaling | -2.828 | 2.683 | 3.128 | 2.353 |
| Netrin Signaling | -2 | 2.183 | 2.324 | 2.183 |
| **Production of Nitric Oxide and Reactive Oxygen Species in Macrophages** | -0.686 | 0.926 | 2.885 | 1.414 |
| Cardiac β-adrenergic Signaling | -1.886 | 2.2 | 1.732 | 1.964 |
| UVB-Induced MAPK Signaling | -1.069 | 1.5 | 1.606 | 0.943 |
| ErbB4 Signaling | -1.807 | 2.324 | 2.4 | 1.789 |
| Calcium-induced T Lymphocyte Apoptosis | -1.069 | 0.471 | 1.964 | 1.606 |
| Acute Myeloid Leukemia Signaling | -1.606 | 1.528 | 1.225 | 1.569 |
| Relaxin Signaling | -3 | 2.982 | 2.449 | 2.982 |
| NF-κB Activation by Viruses | -2.324 | 2.357 | 3.411 | 2.985 |
| Superpathway of Inositol Phosphate Compounds | -2.197 | 2.16 | 3.051 | 3.244 |
| PAK Signaling | -2.065 | 2.982 | 2.132 | 1.964 |
| Prolactin Signaling | -2 | 2.524 | 3.128 | 2.294 |
| ILK Signaling | -1.616 | 1.543 | 1.941 | 1.896 |
| Thrombopoietin Signaling | -2.138 | 2.5 | 2.837 | 2.4 |
| STAT3 Pathway | -2.236 | 1.964 | 2.887 | 2.502 |
| SAPK/JNK Signaling | -1.342 | 2.4 | 2.6 | 1.8 |
| **NRF2-mediated Oxidative Stress Response** | -1.4 | 1.4 | 0.898 | 0.174 |
| Melanocyte Development and Pigmentation Signaling | -2.828 | 2.236 | 2.2 | 2.524 |
| RhoA Signaling | -1.043 | 2.058 | 2.414 | 1.177 |
| FcγRIIB Signaling in B Lymphocytes | -0.707 | 1.667 | 1.732 | 1.265 |
| PEDF Signaling | -2.183 | 1.291 | 2 | 2.357 |
| Breast Cancer Regulation by Stathmin1 | -4.001 | N/A | N/A | N/A |
| VEGF Family Ligand-Receptor Interactions | -2 | 1.414 | 1.569 | 1.877 |
| D-myo-inositol-5-phosphate Metabolism | -1.732 | 1.616 | 2.53 | 2.785 |
| IL-15 Signaling | -1.807 | N/A | N/A | N/A |
| JAK/Stat Signaling | -2.5 | 1.886 | 2.982 | 2.683 |
| PDGF Signaling | -3.153 | 2.828 | 3.273 | 3.9 |
| Apoptosis Signaling | 2.524 | -1.964 | -1.4 | -1.633 |
| Gαi Signaling | -1.964 | 1.706 | 0.186 | 1.46 |
| Non-Small Cell Lung Cancer Signaling | -1.941 | 2.5 | 2.357 | 2.357 |
| PKCθ Signaling in T Lymphocytes | -2.236 | 1.46 | 4.596 | 3.413 |
| Role of IL-17F in Allergic Inflammatory Airway Diseases | -2.53 | 1.667 | 1.897 | 1.667 |
| TWEAK Signaling | -0.333 | 0.707 | 0.302 | 0 |
| CDK5 Signaling | -2.524 | 2.132 | 1.512 | 1.706 |
| IL-1 Signaling | 0.378 | 0 | 0.577 | 0 |
| Leptin Signaling in Obesity | -1.342 | 1.134 | 1.265 | 1.134 |
| LPS-stimulated MAPK Signaling | -1.5 | 1.213 | 2.132 | 1.279 |
| Pancreatic Adenocarcinoma Signaling | -1.213 | 0.853 | 1.225 | 1.877 |
| **Antioxidant Action of Vitamin C** | 0.229 | -0.218 | -1.3 | -0.784 |
| Phospholipases | -0.277 | 0.277 | 1.091 | 0.728 |
| 3-phosphoinositide Degradation | -2.117 | 1.976 | 2.535 | 3.024 |
| Regulation of Cellular Mechanics by Calpain Protease | -1.667 | 0.577 | 0.905 | 2.111 |
| Angiopoietin Signaling | -3.051 | 2.138 | 2.324 | 3.5 |
| IL-6 Signaling | -2.4 | 1.877 | 3.157 | 2.556 |
| ERK5 Signaling | -2.673 | 1.213 | 1.886 | 2.524 |
| GM-CSF Signaling | -3.317 | 3.051 | 3.207 | 2.5 |
| Oncostatin M Signaling | -2.333 | 2.333 | 2.714 | 2.53 |
| 3-phosphoinositide Biosynthesis | -2.117 | 2.263 | 2.795 | 3.286 |
| Dendritic Cell Maturation | -0.192 | 0.73 | 3.618 | 2.846 |
| NF-κB Signaling | -2.785 | 3.087 | 3.795 | 3.893 |
| PPAR Signaling | 1.886 | -1.279 | -2.268 | -2.353 |
| Macropinocytosis Signaling | -1.508 | 1.508 | 1.941 | 1.604 |
| HMGB1 Signaling | -1.091 | 1.134 | 1.095 | 0.73 |
| Actin Nucleation by ARP-WASP Complex | -1.155 | 0.5 | 0.577 | 0 |
| Insulin Receptor Signaling | -1.789 | 2.041 | 2.191 | 1.732 |
| mTOR Signaling | -1.569 | 0.707 | 1.029 | 1.48 |

Supplementary Table S12. Cellular functions affected by ILB^®^ in the sTBI model.

Numerical values are the activation z scores (z>0 activation; z<0 inhibition) of the pathways based on the differential gene expression in the different data-sets.

| **Cellular Functions** | **sTBI** | **sTBI + ILB^®^ 1mg/kg** | **sTBI + ILB^®^ 5mg/kg** | **sTBI + ILB^®^ 15mg/kg** |
| --- | --- | --- | --- | --- |
| Cell movement | -6.943 | 5.38 | 8.335 | 6.696 |
| Migration of cells | -6.635 | 4.73 | 7.946 | 6.494 |
| Cell survival | -6.178 | 4.869 | 5.917 | 5.846 |
| Organization of cytoskeleton | -6.125 | 5.366 | 5.835 | 5.557 |
| Organization of cytoplasm | -6.075 | 5.323 | 5.793 | 5.51 |
| Cell viability | -5.803 | 4.536 | 5.616 | 5.637 |
| Microtubule dynamics | -5.832 | 4.992 | 5.395 | 5.415 |
| Development of neurons | -6.078 | 5.388 | 4.961 | 5.159 |
| Neurotransmission | -4.036 | 4.024 | 3.957 | 3.537 |
| Neuritogenesis | -4.472 | 3.951 | 3.266 | 3.572 |
| Morphogenesis of neurons | -4.472 | 3.951 | 3.266 | 3.572 |
| Angiogenesis | -4.136 | 2.578 | 3.778 | 4.084 |
| Synaptic transmission | -3.464 | 3.314 | 3.305 | 2.991 |
| Vasculogenesis | -3.757 | 2.416 | 3.694 | 4.015 |
| Learning | -4.288 | 3.639 | 3.074 | 2.73 |
| Long-term potentiation | -3.298 | 3.163 | 2.663 | 2.738 |
| Apoptosis | 3.58 | -2.958 | -2.756 | -3.127 |
| Cellular homeostasis | -2.799 | 2.502 | 3.992 | 2.532 |
| Memory | -3.43 | 3.236 | 2.802 | 2.87 |
| Transport of molecule | -2.141 | 1.752 | 3.671 | 3.38 |
| Shape change of neurites | -3.274 | 3.056 | 2 | 2.27 |
| Proliferation of neuronal cells | -2.415 | 1.572 | 1.973 | 2.645 |
| Necrosis | 1.878 | -1.743 | -0.978 | -1.506 |
| Transport of metal |  | 1.531 | 1.623 | 2.207 |
| Transport of ion | -1.416 | 1.306 | 1.005 | 1.257 |
| Concentration of lipid | -0.907 | 0.177 | 0.71 | 1.258 |
| Neuronal cell death | 0.841 | -0.852 | -0.599 | -0.371 |
| Locomotion | 0.283 | -0.575 | -0.311 | -0.317 |
| Metabolism of reactive oxygen species |  |  |  | 2.157 |
| Synthesis of reactive oxygen species |  |  |  | 2.14 |
| Degeneration of neurons |  |  |  |  |
| Cellular degradation |  |  |  |  |
| Emotional behavior | -0.275 | 0.001 | -0.588 | -0.635 |
| Behavior |  |  |  |  |
| Invasion of cells | -4.195 | 3.495 | 5.285 | 4.529 |
| Transcription of RNA | -3.584 | 2.963 | 3.877 | 3.999 |
| Quantity of neurons | -3.989 | 2.775 | 2.748 | 4.033 |
| Quantity of nervous tissue | -3.965 | 2.762 | 2.672 | 4.006 |
| Dendritic growth/branching | -2.983 | 3.283 | 1.447 | 1.86 |
| Phosphorylation of protein | -1.144 | 2.424 | 3.091 | 2.561 |
| Conditioning | -2.901 | 2.488 | 1.732 | 1.529 |
| Synthesis of lipid | -2.387 | 1.437 | 2.555 | 2.727 |
| Coordination | -2.796 | 4.236 |  |  |
| Transactivation of RNA | -3.489 | 3.189 | 3.287 |  |
| Branching of cells | -2.775 | 2.057 | 1.314 | 2.398 |
| Sprouting | -2.713 | 2.245 | 1.419 | 2.086 |
| Growth of neurites | -2.079 | 1.421 | 1.754 | 2.387 |
| Concentration of hormone | -1.08 | 1.184 | 2.347 | 1.07 |
| Quantity of connective tissue |  | 1.294 | 2.18 | 1.976 |
| Quantity of carbohydrate | -1.673 | 1.869 | 1.918 |  |
| Synthesis of nitric oxide | -1.474 | 0.748 | 1.346 | 1.623 |
| Axonogenesis | -1.497 | 0.92 | 1.747 | 1.893 |
| Differentiation of connective tissue cells | -1.469 | 1.71 | 1.217 | 1.09 |
| Outgrowth of neurites | -1.565 | 0.796 | 1.252 | 2.143 |
| Differentiation of nervous system | -1.832 | 1.257 | 1.948 |  |
| Quantity of metal | -1.354 | 1.075 | 1.889 | 1.197 |
| Quantity of metal ion | -1.509 | 0.905 | 1.674 | 1.203 |
| Excitatory postsynaptic potential | -2.559 | 0.765 | 1.342 | 0.816 |
| Innervation | -1.999 |  |  | 1.215 |
| Secretion of molecule | -0.868 | 0.258 | 1.762 | 1.52 |
| Collapse of growth cone |  |  |  |  |
| Activation of cells |  |  | 2.011 | 1.79 |
| Cell death of muscle cells |  | -0.554 | -1.234 | -1.118 |
| Cell cycle progression | -1.357 | 0.6 |  | 1.16 |
| Long term depression |  |  |  |  |
| Quantity of Ca2+ | -0.931 | 0.442 | 0.923 | 0.562 |
| Proliferation of muscle cells |  | 1.258 |  | 1.04 |
| Action potential of neurons |  | 0.995 | 1.04 |  |
| Metabolism of hormone |  | 0.911 |  |  |
| Muscle contraction |  |  | 1.684 |  |
| Production of reactive oxygen species |  |  |  | 1.489 |
| Function of smooth muscle |  |  |  | 0.886 |

Supplementary Table S13. Upstream regulators affected by ILB^®^ in the sTBI model.

Numerical values are the activation z scores (z>0 activation; z<0 inhibition) of the pathways based on the differential gene expression in the different data-sets.

| **Upstream Regulators** | **sTBI** | **sTBI + ILB^®^ 1mg/kg** | **sTBI + ILB^®^ 5mg/kg** | **sTBI + ILB^®^ 15mg/kg** |
| --- | --- | --- | --- | --- |
| TGFB1 | -5.754 | 5.461 | 7.076 | 6.14 |
| Vegf | -4.292 | 3.133 | 4.045 | 4.266 |
| BDNF | -3.668 | 4.804 | 3.727 | 3.032 |
| Tgf beta | -3.715 | 3.56 | 4.207 | 4.334 |
| HGF | -3.959 | 3.48 | 3.363 | 3.804 |
| FGF2 | -3.384 | 2.897 | 3.912 | 3.915 |
| EGF | -3.437 | 3.476 | 3.899 | 3.158 |
| IL4 | -2.945 | 2.288 | 4.157 | 3.608 |
| PDGF BB | -2.556 | 2.831 | 4.035 | 3.6 |
| IL6 | -3.181 | 1.644 | 3.975 | 4.208 |
| WNT3A | -2.924 | 2.983 | 3.056 | 2.704 |
| GDF2 | -3.15 | 2.24 | 3.583 | 2.898 |
| IL1 |  | 2.283 | 3.552 | 3.419 |
| IL1B | -2.067 | 1.938 | 2.96 | 2.31 |
| TNF | -2.014 | 1.841 | 2.934 | 2.549 |
| LEP | -2.199 | 1.852 | 2.433 | 2.258 |
| GDNF | -2.252 | 2.501 | 1.568 | 1.962 |
| CXCL12 | -1.629 | 2.252 | 2.269 | 0.986 |
| CSF2 |  | 1.781 | 4.31 |  |
| IL13 | -1.519 | 1.287 | 2.16 | 1.03 |
| IL10 | -1.043 | 1.802 | 2.194 | 2.062 |
| Growth hormone | -2.003 | 0.672 | 1.327 | 1.009 |
| IFNG | -0.545 | 0.313 | 2.598 | 1.501 |
| FGF1 | 0.821 | -1.348 | -1.085 | -0.53 |
| GH1 | -0.388 | 1.026 | 1.685 | 0.929 |
| INHA | 1.164 | -0.572 | -1.69 | -0.742 |
| AGT | -0.389 | 0.55 | 2.201 | 1.389 |
| NGF | -0.854 | 1.445 | 0.341 | 0.626 |
| LIF | -1.272 | 0.942 | 0.438 | 0.71 |
| IGF1 | -0.276 | 0.024 | 1.846 | 0.7 |
| IL17A |  |  | 1.805 | 1.043 |
| Ngf | -0.781 |  | 0.615 |  |
| SPP1 |  |  |  | 0.151 |
| VEGFA | -4.532 | 4.727 | 4.901 | 4.534 |
| TGFB2 | -3.276 | 3.658 | 3.232 | 3.4 |
| EDN1 | -2.896 | 2.546 | 2.455 | 2.808 |
| CXCL8 | -2.496 | 2.496 | 2.493 | 2.514 |
| IL2 | -2.285 | 2.142 | 2.851 | 2.93 |
| OSM | -1.733 | 2.149 | 3.889 | 3.127 |
| PRL |  | 2.318 | 4.078 | 3.3 |
| NRTN | -2.801 | 2.47 | 1.786 | 2.616 |
| BMP2 | -3.092 | 1.926 | 2.323 | 2.563 |
| JAG2 | -2.789 | 2.377 | 2.18 | 2.377 |
| IL1A | -1.648 | 2.067 | 3.235 | 2.284 |
| TNFSF11 | -2.072 | 2.157 | 2.668 | 1.989 |
| FGF7 | -1.898 | 2.409 | 2.177 | 2.284 |
| NOG | 2.52 | -2.204 | -1.92 | -1.537 |
| CTGF | -2.069 | 2.081 | 2.697 | 1.884 |
| HBEGF | -1.605 | 2.083 | 1.793 | 1.973 |
| AREG | -2.334 |  | 2.585 | 2.243 |
| CD40LG |  | 2.682 | 2.765 | 1.625 |
| DKK1 | 1.902 | -1.318 | -2.314 | -1.62 |
| TGFB3 |  | 2.251 | 1.989 | 2.048 |
| cytokine |  | 2.534 | 2.94 |  |
| Pdgf (complex) | -1.5 | 1.85 | 2.206 | 1.835 |
| ANGPT2 | -1.045 | 0.954 | 2.543 | 2.193 |
| TGFA | -1.135 | 1.309 | 2.489 | 2.151 |
| NRG1 | -1.271 | 2.034 | 1.936 | 2.232 |
| WNT1 | -1.131 | 1.313 | 2.372 | 1.631 |
| CSF1 | -1.569 | 1.357 | 2.517 | 1.225 |
| EPO | -1.223 | 1.291 | 1.818 | 1.34 |
| Tnf (family) | -2.43 |  | 2.109 | 2.661 |
| BMP6 | -1.228 | 1.999 | 1.809 | 0.913 |
| Interferon alpha |  | 2.119 | 2.687 | 2.067 |
| WNT5A | -1.767 | 1.835 | 1.215 |  |
| KITLG | -1.225 | 0.672 | 1.939 | 1.712 |
| IL27 | -2.799 |  | 2.404 |  |
| CRH | -1.351 | 1.053 | 0.932 | 1.296 |
| VEGFB |  | -1.116 | -1.943 | -1.567 |
| TNFSF13B |  |  | 2.586 |  |
| BMP4 | -0.619 | 1.012 | 1.479 |  |
| NOV | 1.026 | -0.847 | -0.847 | -0.923 |
| INHBA | -0.3 | 0.118 | 1.197 | 1.784 |
| FASLG |  | -2.113 | -0.575 | -0.575 |
| CNTF | -0.721 | -0.812 | 0.525 | 0.77 |
| PDGFB |  |  | 1.897 | 0.712 |
| CSF3 |  | 0.238 | 0.931 | 1.487 |
| IL18 |  | 0.366 | 1.371 |  |
| IL3 | -0.283 |  | 0.983 | 0.745 |
| VEGFC | -0.537 | 0.537 | 0.168 | 1.446 |
| HDL |  | -0.742 | -0.996 | -1.007 |
| GHRL |  | 1.316 | 1.411 |  |
| IL33 |  |  | 2.714 |  |
| NTF4 | -0.294 | 0.61 | 1.094 | 0.438 |
| FGF8 | -0.126 | 0.019 | 0.775 | 0.963 |
| CX3CL1 | -0.727 |  | 0.657 | 1.032 |
| BMP7 |  | -0.39 | -0.072 | -0.729 |
| IGF2 |  | -0.859 |  | -1.043 |
| C5 | -1.067 | 1.067 |  |  |
| C5 |  |  | 2.024 |  |
| IL1RN |  | -0.438 |  | -1.582 |
| IFN Beta |  |  | 1.996 |  |
| TIMP1 |  |  |  |  |
| TNFSF12 |  | -1.826 |  |  |
| IL12 (complex) |  |  | 1.762 |  |
| Ifn gamma |  | 0.011 | 0.917 | 0.773 |
| NDP | -0.747 |  |  |  |
| NTF3 | 1.134 | -0.156 | 0 | 0.164 |
| PDGF-AA |  |  | 0.719 | 0.732 |
| MSTN |  | 0.57 | -0.282 | -0.187 |
| MAC | -0.594 |  |  | 0.594 |
| CCL2 | 0.059 | -0.45 |  | -0.504 |
| CXCL1 | -1 |  |  |  |
| FGF21 | 0.192 | 0.373 | -0.196 | -0.052 |
| IL15 |  |  | 0.552 | -0.156 |
| Il3 |  |  | 0.391 | -0.202 |
| IL32 |  |  | -0.478 |  |
| FGF10 |  |  |  |  |
| CCL5 |  |  |  | -0.314 |
| AMH | -0.29 |  |  |  |
| Ccl6 |  | 0 |  |  |
| ANGPT1 | 0.07 | -0.063 |  |  |

Supplementary Table S14. Typical high-quality calibrator back-calculated values and QC measurements.

Based on the calibrator the back-calculated Gllypican-3 values are compared to the nominal Glypican-3 in the calibrators to calculate the recovery rate of the calibrators and bias in the assay. The %CV is calculated form the triplicate values of back-calculated Glypican-3. It is important to note, Bias and %CV is always calculated based on the back-calculated calibrator values rather than the reading.

| **Calculated pg Glypican protein (average)** | **Nominal Glypican (microg/ml)** | **Calculated average microg/ml Glypican** | **% Recovery** | **% Difference from Calibrator (Bias)** | **% CV of Calibrator** | **Total Error assay** | **Total Error Accepted (TEa)** | **Sigma Metric** | **Calibrator Quality** |
| --- | --- | --- | --- | --- | --- | --- | --- | --- | --- |
| 11771.48 | 12500.00 | 12261.84 | **98.09** | **1.91** | **4.15** | **10.04** | **36.50** | **8.33** | **OK** |
| 6499.10 | 6250.00 | 6575.81 | **105.21** | **5.21** | **2.86** | **10.81** | **36.50** | **10.95** | **OK** |
| 3175.94 | 3125.00 | 3003.87 | **96.12** | **3.88** | **5.06** | **13.79** | **36.50** | **6.45** | **OK** |
| 1637.48 | 1562.50 | 1547.30 | **99.03** | **0.97** | **5.37** | **11.50** | **36.50** | **6.61** | **OK** |
| 840.58 | 781.25 | 804.76 | **103.01** | **3.01** | **4.07** | **10.98** | **36.50** | **8.24** | **OK** |
| 386.35 | 390.63 | 386.28 | **98.89** | **1.11** | **2.34** | **5.71** | **36.50** | **15.10** | **OK** |
| 208.54 | 195.31 | 195.50 | **100.09** | **0.09** | **6.82** | **13.46** | **36.50** | **5.34** | **OK** |
|  | 0.00 |  |  |  |  |  |  |  |  |
|  |  |  | **Average performance** | **2.31** | **4.38** | **10.90** | **36.50** | **7.80** | **OK** |

Supplementary Table S15. Example of ILB^®^ potency calculations.

| ILB^®^ cc (ug/ml) | ILB^®^ cc (M) | EC50 (Glypican-3; pg/ml) | DR | pA2 |
| --- | --- | --- | --- | --- |
| 0 | 0 | 2428.48 |  |  |
| 0.02 | 8.92459E-09 | 4060.07 | 1.67 | 7.88 |

Supplementary Table S16. 95% Confidence intervals for the assay (calibrator values).

| **Glypican calibrator pg/ml** | **N (experimental measurements used, each sample in triplicates)** | **T value for N-1 DF** | **Bias %** | **Stdev (% of men)** | **RE % (1.96CV)** | **CI (Lower) in %** | **CI (Upper) in %** | **Total Error %** |
| --- | --- | --- | --- | --- | --- | --- | --- | --- |
| 12500 | 6 | 2.57 | 4.40 | 5.73 | 11.07 | 90.46 | 102.03 | 15.47 |
| 6250 | 7 | 2.45 | 9.16 | 9.65 | 9.79 | 95.82 | 114.60 | 18.95 |
| 3125 | 7 | 2.45 | 8.46 | 11.75 | 13.52 | 88.17 | 109.67 | 21.98 |
| 1562.5 | 7 | 2.45 | 7.01 | 11.92 | 16.55 | 88.30 | 110.17 | 23.56 |
| 781.25 | 7 | 2.45 | 4.82 | 8.17 | 11.50 | 93.48 | 108.76 | 16.32 |
| 390.625 | 7 | 2.45 | 2.18 | 8.72 | 17.52 | 93.20 | 109.54 | 19.70 |
| 195.3125 | 4 | 3.18 | 0.79 | 7.30 | 15.89 | 87.72 | 110.79 | 16.69 |
|  |  |  |  |  |  |  |  |  |
| Overall performance | 45 | 2.02 | 5.23 | 9.03 | 12.80 | 91.02 | 109.37 | 18.95 |
| Max error possible | 7 | 2.45 | 9.16 | 11.92 | 17.52 | 85.64 | 116.80 | 26.68 |

Supplementary Table S17. Simulated data.

Values in black are the actual measurements from various experiments. Blue numbers are simulated values and subsequent calculations.

| **Total Error (%)** | **ILB^®^ cc (ug/ml)** | **EC50 (Glypican-3)** | **DR** | **pA2** | **pA2 variability relative to average (measured)** | **Dose required for 1/2 response (M ILB^®^)** | **Dose required for 1/2 response (ug/ml ILB^®^)** | **Difference of Dose required for 1/2 response (% diff from range)** | **Log ILB^®^** | **Log (DR-1)** |
| --- | --- | --- | --- | --- | --- | --- | --- | --- | --- | --- |
|  | **0** | **2428.48** |  |  |  |  |  |  |  |  |
|  | **0.02** | **4060.07** | **1.67** | **7.88** | **0.50%** | **1.33E-08** | 0.0298 |  | **-8.05E+00** | **-0.17** |
|  | **0.04** | **5320.61** | **2.19** | **7.82** | **-0.17%** | **1.50E-08** | 0.0336 |  | **-7.75E+00** | **0.08** |
|  | **0.08** | **8042.16** | **3.31** | **7.81** | **-0.33%** | **1.54E-08** | 0.0346 |  | **-7.45E+00** | **0.36** |
| -0.20 | 0.02 | 3654.07 | 1.50 | 7.75 | **-1.08%** | 1.77E-08 | 0.0396 | 14.51% | -8.05E+00 | -0.30 |
| -0.18 | 0.02 | 3694.67 | 1.52 | 7.77 | **-0.90%** | 1.71E-08 | 0.0384 | 10.84% | -8.05E+00 | -0.28 |
| -0.16 | 0.02 | 3735.27 | 1.54 | 7.78 | **-0.73%** | 1.66E-08 | 0.0372 | 7.39% | -8.05E+00 | -0.27 |
| -0.14 | 0.02 | 3775.87 | 1.55 | 7.79 | **-0.56%** | 1.61E-08 | 0.0360 | 4.16% | -8.05E+00 | -0.26 |
| -0.12 | 0.02 | 3816.47 | 1.57 | 7.81 | **-0.39%** | 1.56E-08 | 0.0350 | 1.11% | -8.05E+00 | -0.24 |
| -0.10 | 0.02 | 3857.07 | 1.59 | 7.82 | **-0.24%** | 1.52E-08 | 0.0340 | Within range | -8.05E+00 | -0.23 |
| -0.08 | 0.02 | 3897.67 | 1.60 | 7.83 | **-0.08%** | 1.48E-08 | 0.0331 | Within range | -8.05E+00 | -0.22 |
| -0.06 | 0.02 | 3938.27 | 1.62 | 7.84 | **0.07%** | 1.44E-08 | 0.0322 | Within range | -8.05E+00 | -0.21 |
| -0.04 | 0.02 | 3978.87 | 1.64 | 7.85 | **0.22%** | 1.40E-08 | 0.0313 | Within range | -8.05E+00 | -0.19 |
| -0.02 | 0.02 | 4019.47 | 1.66 | 7.87 | **0.36%** | 1.36E-08 | 0.0305 | Within range | -8.05E+00 | -0.18 |
| 0.02 | 0.02 | 4100.67 | 1.69 | 7.89 | **0.64%** | 1.30E-08 | 0.0290 | -2.43% | -8.05E+00 | -0.16 |
| 0.04 | 0.02 | 4141.28 | 1.71 | 7.90 | **0.77%** | 1.27E-08 | 0.0284 | -4.74% | -8.05E+00 | -0.15 |
| 0.06 | 0.02 | 4181.88 | 1.72 | 7.91 | **0.90%** | 1.24E-08 | 0.0277 | -6.95% | -8.05E+00 | -0.14 |
| 0.08 | 0.02 | 4222.48 | 1.74 | 7.92 | **1.03%** | 1.21E-08 | 0.0271 | -9.05% | -8.05E+00 | -0.13 |
| 0.10 | 0.02 | 4263.08 | 1.76 | 7.93 | **1.15%** | 1.18E-08 | 0.0265 | -11.07% | -8.05E+00 | -0.12 |
| 0.12 | 0.02 | 4303.68 | 1.77 | 7.94 | **1.27%** | 1.16E-08 | 0.0259 | -12.99% | -8.05E+00 | -0.11 |
| 0.14 | 0.02 | 4344.28 | 1.79 | 7.95 | **1.39%** | 1.13E-08 | 0.0254 | -14.83% | -8.05E+00 | -0.10 |
| 0.16 | 0.02 | 4384.88 | 1.81 | 7.96 | **1.51%** | 1.11E-08 | 0.0248 | -16.60% | -8.05E+00 | -0.09 |
| 0.18 | 0.02 | 4425.48 | 1.82 | 7.96 | **1.62%** | 1.09E-08 | 0.0243 | -18.30% | -8.05E+00 | -0.08 |
| 0.20 | 0.02 | 4466.08 | 1.84 | 7.97 | **1.73%** | 1.06E-08 | 0.0238 | -19.93% | -8.05E+00 | -0.08 |
| -0.20 | 0.04 | 4788.55 | 1.97 | 7.74 | **-1.29%** | 1.84E-08 | 0.0412 | 18.93% | -7.75E+00 | -0.01 |
| -0.18 | 0.04 | 4841.76 | 1.99 | 7.75 | **-1.17%** | 1.80E-08 | 0.0403 | 16.31% | -7.75E+00 | 0.00 |
| -0.16 | 0.04 | 4894.96 | 2.02 | 7.76 | **-1.05%** | 1.76E-08 | 0.0394 | 13.80% | -7.75E+00 | 0.01 |
| -0.14 | 0.04 | 4948.17 | 2.04 | 7.76 | **-0.93%** | 1.72E-08 | 0.0386 | 11.40% | -7.75E+00 | 0.02 |
| -0.12 | 0.04 | 5001.38 | 2.06 | 7.77 | **-0.82%** | 1.68E-08 | 0.0378 | 9.09% | -7.75E+00 | 0.03 |
| -0.10 | 0.04 | 5054.58 | 2.08 | 7.78 | **-0.70%** | 1.65E-08 | 0.0370 | 6.88% | -7.75E+00 | 0.03 |
| -0.08 | 0.04 | 5107.79 | 2.10 | 7.79 | **-0.59%** | 1.62E-08 | 0.0363 | 4.76% | -7.75E+00 | 0.04 |
| -0.06 | 0.04 | 5160.99 | 2.13 | 7.80 | **-0.48%** | 1.59E-08 | 0.0355 | 2.72% | -7.75E+00 | 0.05 |
| -0.04 | 0.04 | 5214.20 | 2.15 | 7.81 | **-0.38%** | 1.56E-08 | 0.0349 | 0.76% | -7.75E+00 | 0.06 |
| -0.02 | 0.04 | 5267.41 | 2.17 | 7.82 | **-0.27%** | 1.53E-08 | 0.0342 | Within range | -7.75E+00 | 0.07 |
| 0.02 | 0.04 | 5373.82 | 2.21 | 7.83 | **-0.07%** | 1.47E-08 | 0.0330 | Within range | -7.75E+00 | 0.08 |
| 0.04 | 0.04 | 5427.02 | 2.23 | 7.84 | **0.03%** | 1.45E-08 | 0.0324 | Within range | -7.75E+00 | 0.09 |
| 0.06 | 0.04 | 5480.23 | 2.26 | 7.85 | **0.13%** | 1.42E-08 | 0.0318 | Within range | -7.75E+00 | 0.10 |
| 0.08 | 0.04 | 5533.44 | 2.28 | 7.86 | **0.23%** | 1.40E-08 | 0.0313 | Within range | -7.75E+00 | 0.11 |
| 0.10 | 0.04 | 5586.64 | 2.30 | 7.86 | **0.32%** | 1.37E-08 | 0.0308 | Within range | -7.75E+00 | 0.11 |
| 0.12 | 0.04 | 5639.85 | 2.32 | 7.87 | **0.41%** | 1.35E-08 | 0.0302 | Within range | -7.75E+00 | 0.12 |
| 0.14 | 0.04 | 5693.06 | 2.34 | 7.88 | **0.50%** | 1.33E-08 | 0.0298 | -0.04% | -7.75E+00 | 0.13 |
| 0.16 | 0.04 | 5746.26 | 2.37 | 7.88 | **0.59%** | 1.31E-08 | 0.0293 | -1.65% | -7.75E+00 | 0.14 |
| 0.18 | 0.04 | 5799.47 | 2.39 | 7.89 | **0.68%** | 1.29E-08 | 0.0288 | -3.20% | -7.75E+00 | 0.14 |
| 0.20 | 0.04 | 5852.67 | 2.41 | 7.90 | **0.77%** | 1.27E-08 | 0.0284 | -4.70% | -7.75E+00 | 0.15 |
| -0.20 | 0.08 | 7237.94 | 2.98 | 7.74 | **-1.19%** | 1.80E-08 | 0.0404 | 16.72% | -7.45E+00 | 0.30 |
| -0.18 | 0.08 | 7318.37 | 3.01 | 7.75 | **-1.10%** | 1.77E-08 | 0.0397 | 14.80% | -7.45E+00 | 0.30 |
| -0.16 | 0.08 | 7398.79 | 3.05 | 7.76 | **-1.01%** | 1.74E-08 | 0.0391 | 12.94% | -7.45E+00 | 0.31 |
| -0.14 | 0.08 | 7479.21 | 3.08 | 7.77 | **-0.92%** | 1.72E-08 | 0.0385 | 11.15% | -7.45E+00 | 0.32 |
| -0.12 | 0.08 | 7559.63 | 3.11 | 7.77 | **-0.83%** | 1.69E-08 | 0.0379 | 9.40% | -7.45E+00 | 0.32 |
| -0.10 | 0.08 | 7640.05 | 3.15 | 7.78 | **-0.75%** | 1.66E-08 | 0.0373 | 7.72% | -7.45E+00 | 0.33 |
| -0.08 | 0.08 | 7720.47 | 3.18 | 7.79 | **-0.66%** | 1.64E-08 | 0.0367 | 6.08% | -7.45E+00 | 0.34 |
| -0.06 | 0.08 | 7800.90 | 3.21 | 7.79 | **-0.58%** | 1.61E-08 | 0.0362 | 4.49% | -7.45E+00 | 0.34 |
| -0.04 | 0.08 | 7881.32 | 3.25 | 7.80 | **-0.49%** | 1.59E-08 | 0.0356 | 2.95% | -7.45E+00 | 0.35 |
| -0.02 | 0.08 | 7961.74 | 3.28 | 7.80 | **-0.41%** | 1.57E-08 | 0.0351 | 1.45% | -7.45E+00 | 0.36 |
| 0.02 | 0.08 | 8122.58 | 3.34 | 7.82 | **-0.25%** | 1.52E-08 | 0.0341 | Within range | -7.45E+00 | 0.37 |
| 0.04 | 0.08 | 8203.00 | 3.38 | 7.82 | **-0.18%** | 1.50E-08 | 0.0336 | Within range | -7.45E+00 | 0.38 |
| 0.06 | 0.08 | 8283.42 | 3.41 | 7.83 | **-0.10%** | 1.48E-08 | 0.0332 | Within range | -7.45E+00 | 0.38 |
| 0.08 | 0.08 | 8363.85 | 3.44 | 7.84 | **-0.02%** | 1.46E-08 | 0.0327 | Within range | -7.45E+00 | 0.39 |
| 0.10 | 0.08 | 8444.27 | 3.48 | 7.84 | **0.05%** | 1.44E-08 | 0.0323 | Within range | -7.45E+00 | 0.39 |
| 0.12 | 0.08 | 8524.69 | 3.51 | 7.85 | **0.12%** | 1.42E-08 | 0.0319 | Within range | -7.45E+00 | 0.40 |
| 0.14 | 0.08 | 8605.11 | 3.54 | 7.85 | **0.20%** | 1.40E-08 | 0.0315 | Within range | -7.45E+00 | 0.41 |
| 0.16 | 0.08 | 8685.53 | 3.58 | 7.86 | **0.27%** | 1.39E-08 | 0.0310 | Within range | -7.45E+00 | 0.41 |
| 0.18 | 0.08 | 8765.95 | 3.61 | 7.86 | **0.34%** | 1.37E-08 | 0.0307 | Within range | -7.45E+00 | 0.42 |
| 0.20 | 0.08 | 8846.38 | 3.64 | 7.87 | **0.41%** | 1.35E-08 | 0.0303 | Within range | -7.45E+00 | 0.42 |
