# Supplementary material for: The mechanism of action of a novel neuroprotective low molecular weight dextran sulphate: New platform therapy for neurodegenerative diseases like Amyotrophic Lateral Sclerosis
Source: Front Pharmacol. 2022 Aug 30;13:983853. doi: 10.3389/fphar.2022.983853 (PMC9468270; doi:10.3389/fphar.2022.983853)
Supplement: Supplementary file 15 [file DataSheet1.docx]

Analytical validation of the HGF-Glypican-3 binding assay

# Intended purpose of the assay

The assay is a binding ELISA assay that allows the quantification of Glypican-3 binding to HGF. The assay will be useful for the testing of the effect of heparinoids, like ILB^®^, on the HGF-Glypican-3 binding. The effect of heparin-like molecules can be measured by the measuring the reduction of Glypican-3 binding to HGF.

The assay is designed and validated with the use of human recombinant HGF and human recombinant biotinylated Glypican-3. The assay matrix used is phosphate buffered saline (PBS) with added BSA.

The assay is NOT intended to be used for the detection of HGF binding partners from biofluids.

# Characteristics of the assay

## Analytical performance.

The assessment of the analytical performance of the assay is provided by the continuous internal quality control (IQC).

The adherence to the **Sigma metric** evaluation system^[[1]](#footnote-1)^ is recommended to allow continuous monitoring of assay performance and improvement for assay quality. The minimum quality requirement of the assay is an overall calibrator quality at or above 3 Sigma, with none of the calibrators below 2 Sigma level. This achieves an overall ~90% accuracy.

The required specification for **analytical sensitivity** for the assay is a minimum of 200pg/ml Glypican-3 to be detectable.

The **analytical specificity (exclusivity and selectivity)** of the essay is not tested as the assay was not intended to measure the HGF binding partners from biofluids.

The expectation is that the starting material (human recombinant Glypican-3 and, if applied, heparin-like molecules, like ILB^®^) is dissolved in PBS with fixed amount of BSA and will not contain any potential impurities or interfering substances.

Therefore, there was no effort to identify or reduce the effect of endogenous interferences or cross-reactions.

Values between 80-120% **trueness** of the calibrators are accepted. The accepted **bias** is ≤20%. The expected **precision** (CV% =standard deviation/mean≤10%.) is ≥90%.

There are several ways of estimating the expected **accuracy** of an essay. The most strait forward method was proposed by Westgard^[[2]](#footnote-2)^. According to this, when the accepted bias and imprecision are known, the accepted uncertainty of the result (TEa) can be calculated using the expression:

TEa=Allowable Bias + 1.65 × Allowable Imprecision

Based on the specification expectations above (bias and imprecision) the TEa for the essay is 36.5%. Our specified TEa for the essay (36.5%) is close to the CLIA laboratory expectations.

The assay is expected to be **linear** (R^2^>95%) in the quantification range of the assay.

## Assay technique description

The ELISA plate is coated with HGF (0.2 µg/ml, 100 microL/well) overnight in the refrigerator. The Glypican-3 binding is established with a calibrator (top Glypican concentration 12.5 μg/ml Glypican-3 (biotinylated), 2 fold dilutions and a negative control in PBS containing 0.1% BSA).

To test the effect of ILB^®^ (or other heparinoids) on Glypican binding the assay has been performed with the same Glypican calibrator with added fixed concentrations of ILB^®^.

All calibrators and samples added to the plate in triplicates (100 μl/well) are added to the wells for 30 minutes. After three washes with PBS, Streptavidin-HRP (SA-HRP) is added to the wells (in PBS with 0.1% BSA) for 1 hour.

After a further 3 washes colour development is achieved using the Substrate reagent pack (R&D systems, DY999) as directed by the manufacturer.

Colour intensity is quantified from measurements at 450nm and 540nm (background).

## Data analysis

All analyses described are performed in Excel.

### Calibrator calculation.

Each plate has a separate calibrator, even if plates are processed in parallel on the same day.

The signal intensity is converted to fractions (expressed in %) of the maximum intensity measured with the calibrator for each plate separately, using the formula:

Fraction (%) Max signal=100*(signal-Signal_MIN_)/( Signal_Max_ - Signal_MIN_)

The triplicate calibrator values are averaged and logged. The Glypican concentration is also logged.

These data are used to set the calibration curve to allow the back-calculation of the Glypican-3 concentration from the reading intensity (Supplementary figure S4).

The resulting formula allows the back calculation of calibrator values, where x is the log(Fraction of Max signal) and y=log(Glypican concentration). The final back-calculated Glypican-3 concentration cc=10^y^. Additionally the formula is used to calculate the Glypican-3 concentration required to elicit 50% of the maximum signal intensity (EC50_C_). These values are used for the QC of the assay for each plate (see QC expectations above – example in Supplementary Table S14).

### Calculation of sample values

Once the plate QC is passed (see later for criteria) the sample Glypican-3 concentrations are calculated with the formulae defined with the calibrator.

The CV% of sample variability is calculated for each sample from the sample triplicates for additional QC.

### Calculation of competitor (ILB) potency

For the calculation of potency of the heparin-like molecules tested with the essay, we rely on the assumption that these chemicals affect HGF-Glypican-3 binding like heparin is known to affect it. Therefore, all calculations assume that these molecules act as competitive antagonists of Glypican-3 in this binding assay.

The addition of fixed concentration of ILB (heparin-like molecule) to the calibrators (and applying the same process as described above for the calibrator) allows the calculation of the Glypican-3 concentration required to elicit 50% of the maximum signal intensity (EC50_ILB_).

The EC50 values obtained from the Glypican-3 calibrator with and without ILB allow the calculation of the dose ratio: DR=EC50_ILB_/EC50_C_;

where EC50_ILB_ is the Glypican required to elicit 50% of the maximum signal intensity with ILB present; and EC50_C_ is the Glypican required to elicit 50% of the maximum signal intensity without ILB (the EC50 of the assay calibrator).

The potency of the ILB as an antagonist of Glypican-3 is calculated by the formula:

pA2= LOG(DR-1)-LOG(CC_ILB_);

where CC_ILB_ is the fixed concentration of the ILB added to the Glypican-3 calibrators expressed in M. Several different fixed concentrations of ILB in the assay will allow the derivation of the Schild plot, that describes competitive antagonism (Figure 2A). The calculated potency value (pA2) also allows the calculation of the dose required to reduce the Glypican-3 binding to 50% (Supplementary Table S15) using the formula:

CC_1/2_ = 10^pA2^ (M).

### Traceability of calibrators and controls

Each new batch of HGF and Glypican-3 should be recalibrated against the previous batch. The same applies for each new batch of antibodies used in assay. Recalibration data to be kept for 10 years after the completion of the study.

### Parameters to be used to determine the acceptability of the analytical performance of the assay

For each run it is acceptable for the Calibrators to have no more than 20% Bias and no more than 10% imprecision (CV) with **Total error not exceeding 36.5%**.

The Sigma metrics of the overall calibrator should not fall below 3 (with individual calibrator never falling below 2).

Additionally, it is expected the Calibrator linearity (correlation of nominal and back-calculated Calibrator values) should be at or above R^2^=95%.

# Analytical performance

## Analytical sensitivity

Sensitivity is defined as the lowest concentration of analyte that can be distinguished from background noise or the smallest amount of a substance that can accurately be measured by a method or test system.

At present (with the settings of the assay) the lowest level of Glypican-3 on the Calibration curve is 195.3125 pg/ml. The readings are significantly above the background. This is the minimum level of Glypican detected by the assay (level of detection). The sensitivity of the assay was not tested further Supplementary Figure S5).

## Analytical specificity

Analytical specificity is the capability of a method to discriminate between the analyte of interest and other components of the sample including matrix components. As described above, this is not applicable and was not determined. Therefore, the assay is not validated for the measurement of HGF-Glypican-3 binding in biofluids.

## Control of known relevant exogenous interference and cross-reactions;

While systematic testing was not performed, the suspected possible interfering elements below were eliminated when performing the validation experiments.

### Assay temperature

The nature of the assay is such (binding assay and antibody based detection system), that it will be temperature sensitive. We have tested variations (not in a systematic manner) from room temperature (18-22^0^C) to 37^0^C for the Glypican binding step of the assay and once for the antibody binding step.

The increase in the temperature does increase the binding (marginally) and the antibody-based detection (significantly). The substrate reaction is accelerated significantly at high temperatures.

Since the assay requires a full range of calibrators and the saturation of the binding it is strongly recommended to carry out all parts of the assay in a temperature-controlled laboratory with temperatures set at 20-22^0^C. It is important to avoid significant temperature variations within the room and on work-bench.

### Water quality

Heparin-like molecules in general are known to bind to the surface of bacteria, fungi and viruses. **The binding is irreversible**. This means that even a mild contamination of any assay component (including wash buffers) will have a significant effect on the free heparinoid in the system and therefore the assay outcome that measures the effect of heparinoids on HGF-Glypican-3 interaction.

The deionised water generated by standard laboratory water purification systems may not be able to produce clean enough water for the assay, not even for the wash buffers.

It is therefore recommended that the water used for the assay, including wash buffers, to be tissue culture grade (sterile) ultrapure water with appropriate quality certificate.

All plasticware used for the assay needs to be sterile. All buffers (even wash solutions) need to be made in sterile containers and used only on the day they were made. No buffer stock solution to be used beyond its best by date or beyond 2 weeks from opening (storage in fridge).

### Plasticware

Heparin binds to different types of plastic and glass. It has been documented that some plasticware may lead to a heparin depletion of up to 15% from the therapeutic concentration heparin solutions. If heparins are used in the assay and their concentrations are far below the therapeutic concentrations, the use of glass or polystyrene assay tubes or dispensing dishes may affect the assay significantly depending on the storage time.

It is therefore recommended that all tubes used for the dilution and dispensing of heparin solutions be polypropylene tubes (sterile!). Additionally, all solutions have to be made just before use.

## Trueness (bias)

Trueness represents the closeness of the measurement to the actual value of the analyte in the essay. The systematic deviation of the measurement from the actual value (due to the shortcoming of the essay procedure and equipment) is called the bias. The measurement of trueness is the recovery rate. This was originally set to be acceptable between 80-120% (accepted bias 20%). The recovery rate of the essay is well within expectations of 80-120% at all measurement levels and all plates (Supplemental Figure S6) with the experimental Bias well below the 20% expected (Supplemental Figure S7).

## Precision (repeatability and reproducibility)

The precision of the assay depends on operator error. The measure of precision is the random error (RE) of the assay or the coefficient of variance (CV=STdev/Mean, expressed as %). The expectation was to achieve a CV≤10% (for lower calibrator values %CV≤20%).

Overall, RE of the assay is below the specified 10%. However, it is clear that some calibrators in some experiments may actually show a random error that is above the specifications (Supplementary figure S8). This seems to get less with the accumulation of experiments (practice). While it is clear that it is possible to keep RE below the specification, operator training and pipetting techniques (with repeat pipetting) is paramount to keep operator error at the minimum possible. Plates with average RE>10% are excluded (repeat experiment required) – see acceptance criteria defined below.

## Accuracy (resulting from trueness and precision)

The total Error (TE) of an assay can be calculated as the (Bias+1.96*CV) of an assay.

Accuracy data should be calculated from at least 40 samples. 95% Confidence intervals (CE) for each measurement were also calculated from the data. Generally, TE can be kept (on average) below 30%. Plates, where the total error exceeds the TAE (Total accepted error – definitions below) of the assay (36.5% - see below) would be rejected (see rejection criteria below). The assay performance overall is very good, and with appropriate rejection criteria the total error rate can be kept below the TAE rate of the assay (Supplementary Figure S9).

## Confidence Intervals.

For 95% CI calculations we used standard procedures and included not just the accepted but the rejected plate data as well to reflect the real confidence in the data produced.

95% Confidence Intervals (on average) are within 90-110% of the nominal expected value of the analyte. The 95% CI for all measurements fall within 85-117% of the nominal values as long as acceptance criteria (see below) are respected (Supplemental table S16).

## Rejection criteria.

To support the analytical validation and the continuous QC of the essay we set up the Sigma Metrics based QC system for the Calibrators. Based on the original settings of the QC expectations (Bias 20%, accepted %CV 10%) the maximum accepted error TEa=(Bias+1.65%CV) would come to 36.5%.

The sigma metrics measures (SM) the relationship between the total error (TE) of the assay and the TAE set as QC expectation. Calibrators below a SM=2 should be eliminated. The essay should be rejected (Supplementary figure S10 – Sigma Metrics of accepted experiments).

The experiments show that it is relatively easy to achieve SM>2 (acceptable calibrator quality) with the TAE of 36.5% set at the beginning.

### Formal rejection criteria set for the assay.

**Assays should also be rejected if:**

1. **average %CV>10% OR**
2. **TE>TAE OR**
3. **SM<2.**

## Limits of detection and quantitation (Measuring range)

The assay relies on the measurement of Glypican-3 bound to HGF. As such it is a quantitative assay with a lower limit of detection (LOD). The LOD is the lowest level of analyte detectable by the assay. In our current tests this is set to 195.31 pg/ml Glypican. The LD is also the lower limit of quantification (LLQ). The upper limit of quantification is 12,500 pg/ml Glypican-3.

## Linearity

It is important to have a strong linear relationship between the actual and calculated value of an analyte. The calculations have been carried out with the average, minimum and maximum value of the calculated calibrators for all assays to have a feel about the linearity, and its variations, of the assay.

## Robustness

No formal robustness analysis was performed to measure the effect of temperature variations, buffer concentration variations (taking into account the precision of the serological pipettes used), effect of Glypican-3, HGF and antibody concentrations variations (taking into account the precision of the pipettes used).

While the binding assay itself is very accurate and reliable, if performed correctly, the error in the assay will be amplified by the calculations of antagonist potency. To illustrate the effect of assay error, a simulation was performed to assess the effect of the assay variability (Total error up to 20%) on the estimated effect of heparin-like molecules on the binding assay and subsequent calculations regarding the competitive antagonism of these molecules (Supplementary figure 12).

The simulation relied on the assay output from experiments with different concentrations of ILB (data in Supplementary table S17). The simulation draws attention to several findings:

1. The lower concentration of ILB^®^ tested (0.02 μg/ml) leads to higher variability within the test results
2. The variability in the binding test results lead to a significantly higher variability in the calculated dose of ILB^®^ required to reduce the binding signal to 50%
3. With lower concentration of ILB^®^ tested the variability of the binding assay may make data interpretation more difficult.

1. Kashyap A, Sampath S, Tripathi P, Sen A. Sigma Metrics: A Valuable Tool for Evaluating the Performance of Internal Quality Control in Laboratory. J Lab Physicians. 2021 Jun 28;13(4):328-331. doi: 10.1055/s-0041-1731145. [↑](#footnote-ref-1)
2. https://www.westgard.com/ [↑](#footnote-ref-2)
